# A System to Automatically Classify and Name Any Individual Genome-Sequenced Organism Independently of Current Biological Classification and Nomenclature

**DOI:** 10.1371/journal.pone.0089142

**Published:** 2014-02-21

**Authors:** Haitham Marakeby, Eman Badr, Hanaa Torkey, Yuhyun Song, Scotland Leman, Caroline L. Monteil, Lenwood S. Heath, Boris A. Vinatzer

**Affiliations:** 1 Department of Computer Science, Virginia Tech, Blacksburg, Virginia, United States of America; 2 Department of Statistics, Virginia Tech, Blacksburg, Virginia, United States of America; 3 Department of Plant Pathology, Physiology and Weed Science, Virginia Tech, Blacksburg, Virginia, United States of America; 4 INRA, UR0407 Pathologie Végétale; Montfavet, France; 5 This Genomic Life Inc., Blacksburg, Virginia, United States of America; University of the West of England, United Kingdom

## Abstract

A broadly accepted and stable biological classification system is a prerequisite for biological sciences. It provides the means to describe and communicate about life without ambiguity. Current biological classification and nomenclature use the species as the basic unit and require lengthy and laborious species descriptions before newly discovered organisms can be assigned to a species and be named. The current system is thus inadequate to classify and name the immense genetic diversity within species that is now being revealed by genome sequencing on a daily basis. To address this lack of a general intra-species classification and naming system adequate for today’s speed of discovery of new diversity, we propose a classification and naming system that is exclusively based on genome similarity and that is suitable for automatic assignment of codes to any genome-sequenced organism without requiring any phenotypic or phylogenetic analysis. We provide examples demonstrating that genome similarity-based codes largely align with current taxonomic groups at many different levels in bacteria, animals, humans, plants, and viruses. Importantly, the proposed approach is only slightly affected by the order of code assignment and can thus provide codes that reflect similarity between organisms and that do not need to be revised upon discovery of new diversity. We envision genome similarity-based codes to complement current biological nomenclature and to provide a universal means to communicate unambiguously about any genome-sequenced organism in fields as diverse as biodiversity research, infectious disease control, human and microbial forensics, animal breed and plant cultivar certification, and human ancestry research.

## Introduction

A classification and naming system for life on earth that is accepted and used by all members of the scientific community is a prerequisite for biological research. This is the reason why Carl Linnaeus’ invention of a hierarchical classification and naming system [1], [2] was instrumental to the development of the life sciences. The Darwinian concept of common descent [3] and the advent of DNA sequencing have substantially changed biology over time and brought concomitant adjustments to the original Linnean classification system. However, today we are facing yet another challenge in biological classification. The revolution in DNA sequencing technology is now allowing us to sequence genomes of any size at low cost and is revealing a level of genetic diversity that cannot be classified and named appropriately within the current biological classification system.

Motivated by these concerns, we propose here the idea for a new exclusively genome-based classification and naming system to complement the current biological classification system. The system we propose consists of codes, which are assigned to each individual genome-sequenced organism. Assignment of the proposed codes is based on the measured similarity of an organism’s genome to the genome of the most similar organism that already has a code at the time. We see the following three advantages of the proposed system: 1. codes could be assigned as soon as an organism’s genome is sequenced independently of any lengthy phylogenetic or phenotypic analysis; 2. codes could be permanent - they would not need to be revised when codes are assigned to additional related organisms; and 3. codes could be assigned to all life forms including viruses, bacteria, fungi, plants, and animals providing a standardized naming system for all life on earth.

Here we first point out three important limitations of the current biological classification and nomenclature system. We then describe in detail the concept behind the genome-based codes we propose, assign provisional codes to different life forms with different degrees of diversity, and provide examples of applications of genome-based codes in biological sciences and beyond.

## Limitations of Current Biological Classification and Nomenclature

### Belonging to the Same Species is Poorly Predictive of Similarity between Individuals

Since the early development of biological classification, the species has been the most important unit and has been extremely useful in describing and communicating about the diversity of life on earth. However, there is still no agreement among biologists about the definition of species, in particular, in regard to bacterial species. Therefore, different species are characterized by very different degrees of similarity of the organisms that they encompass. For example, organisms belonging to one species may all be derived from a very recent ancestor and be genetically and phenotypically extremely similar to one other. On the other hand, organisms belonging to another species may be derived from a more distant ancestor and be genetically and phenotypically much more different from each other. Therefore, belonging to the same species is generally a predictor of common ancestry but not a predictor of how similar organisms are to one other.

Interestingly, bacterial species are the only species whose descriptions actually include a measurement of similarity. In fact, bacterial species are described based on phenotypic characteristics in combination with a well-defined cutoff of DNA similarity corresponding to an experimentally determined value of 70% DNA-DNA hybridization (DDH) [4] or similar cutoffs based on other measures of DNA similarity [5], [6]. However, because 70% is a *maximum* cutoff and some bacterial species are characterized by much lower DDH values, some bacterial species are genetically and phenotypically monomorphic, such as *Bacillus anthracis*, the causative agent of anthrax [7], while other bacterial species are genetically and phenotypically much more diverse, such as *Escherichia coli* [8]. Therefore, even though the degree of genetic similarity between organisms is taken into account in bacterial species descriptions, bacterial species do not uniformly encompass organisms with comparable degrees of similarity.

In “phylogenetic nomenclature” [9], names are not given to taxonomic ranks but to clades. This avoids the subjectivity associated with naming taxonomic ranks. Phylogenetic nomenclature also provides rules for unambiguous naming of clades. However, since organisms that belong to the same clade may still be very similar or different from each other, phylogenetic nomenclature does not address the problem of names being non-predictive of the diversity of the organisms that are associated with them either.

In summary, current biological classification and nomenclature do not provide any means to classify and name groups of organisms that are characterized by the same degree of similarity resulting in taxa that do not show comparable genetic diversity leading to a system that is not strongly predictive of genetic relatedness.

### There is No General System for Intraspecific Classification

The second issue with current biological classification is that today almost any individual bacterial or fungal isolate or plant or animal can be distinguished from any other individual using DNA sequencing. Based on partial or complete genome sequences, organisms can then be assigned to intraspecific classes. However, there is no general system to define intraspecific classes based on DNA similarity and there are no general rules to name such classes making it impossible to take full advantage of genome sequencing for intraspecies classification.

Multilocus sequence typing (MLST) has emerged as one promising approach to solve this problem by assigning bacteria to genetic lineages, called sequence types (STs), which have identical alleles at a small number of genomic loci [10]. However, MLST presents several limitations: (i) since only six to eight genomic loci are typically used, each ST still includes isolates with a considerable amount of genetic diversity that is not classified; (ii) since different MLST schemes use different loci, MLST schemes have different resolutions leading to STs of different genetic diversity; (iii) ST names do not provide any information about the relationship between STs (bacteria belonging to two different STs may be very closely related or only distantly related); and (iv) MLST is not hierarchical, providing only one level of resolution (diversity within a single ST or similarity between STs is not considered). Ribosomal MLST (rMLST) is based on 53 genes coding for the same ribosomal proteins present in almost all bacteria [11] and alleviates some of these problems. However, even rMLST has still three fundamental shortcomings: (i) it is not hierarchical; (ii) resolution is limited by using a restricted set of loci instead of whole genomes; and (iii) rMLST ST numbers are not informative of the relationships between different STs.

Besides MLST, other classification systems have been developed for other specific groups of organisms. For example, for many viral species, numbers are assigned to different intraspecific sub-groups, and, in human genetics, a system for classification of mitochondrial genomes has been devised that assigns individuals to mitochondrial haplogroups based on polymorphic regions in mitochondrial genomes [12]. Although these different intraspecific classification systems are relatively useful for scientists working with specific species, they present a series of weaknesses: they each have a different resolution, they each use different methods to assign individuals to classes, and they each use different naming conventions. Therefore, today’s intraspecies classification systems represent high barriers to communication about intraspecific diversity and hinder understanding of intraspecific diversity by the general public.

### Species Descriptions and Names are Unstable

Lastly, species descriptions change with discovery of new diversity and/or identification of additional genetic or phenotypic characterization of organisms belonging to a species. This leads to recurrent revisions of species descriptions, which may cause individual taxa to be assigned to different species changing the species name that is used to refer to them. This is especially true for bacteria, but also for animals and plants for which revisions are regularly published in systematics journals. Moreover, an extensive revision of fungal species names is currently under way, transitioning from naming pleomorphic fungi with two separate names to using single names [13]. Although the end result of this revision can be expected to significantly reduce confusion in fungal taxonomy, in the short term these changes will create more confusion. Importantly, changes in species descriptions and/or names not only represent a challenge for researchers, they can have dangerous implications for medical diagnostics when they concern pathogenic organisms. Such changes in species descriptions can lead to miscommunication between medical personnel about the identity of pathogens, thereby compromising the application of the most appropriate treatment.

To address these challenges in today’s world where hundreds or thousands of new genome sequences are obtained daily but in the absence of any means to classify and name these organisms at a similar speed, we propose the introduction of informative genome similarity-based codes that can be assigned automatically to every single genome-sequenced organism completely independently of current classification and nomenclature. Importantly, we do not claim that the proposed classification and naming system is the only possible solution to the described challenges and we do not expect that the described approach will be applied precisely the way we used it in the examples below. Our goal here is simply to show that a classification and naming system of individual organisms based exclusively on genome similarity is feasible and would be extremely useful in many fields of biological sciences and for society at large. On the other hand, we show that a system based on phylogenetic inference would be impossible to use to automatically classify and stably name individual organisms.

### The Key Principle behind Genome Similarity-Based Codes

The key principle of the system of genome similarity-based codes (simply referred to as “genome codes” or “codes” from here on) described herein consists in assigning to each individual organism (or viral or bacterial isolate) a unique code that expresses the similarity of its genome to all related organisms, i.e., all organisms that have genomes similar enough to be aligned with each other. Similar to Linnaean and phylogenetic classification, the proposed codes are hierarchical: codes consist of 24 positions–but additional positions could be added–whereby every position in the code reflects a different level of similarity between organisms–measured as percentage of DNA identity. The first code position (left-most, called A) reflects the lowest level of similarity and the last code position (right-most, called X) reflects the highest level of similarity. In other words, each position in the code indicates a “bin” similar to an “operational taxonomic unit” [14], whereby the bin size decreases moving from the left to the right of the code. Therefore, (i) two organisms with very similar genomes only differ at position X in their codes, (ii) very different genomes differ already at position A of their codes, while (iii) two organisms with intermediate similarity will be identical to each other at several left-most positions and be different at one of the central positions of the code. Importantly, the actual numeric value at a position does not express similarity. For example, two organisms with a “3” and “4” at one position are not necessarily more similar to each other than two organisms with a “10” and “100” at that position. The information content of genome codes consists exclusively in the extent of shared code positions: the more similar the genomes of two organisms are, the further to the right the values at their code positions will be identical.

Since eukaryotes also have a separate mitochondrial genome, eukaryotes could also be assigned a mitochondrial code. Additionally, male animals could be assigned a Y-chromosome code and plants a chloroplast code.

### Assignment of Genome Codes

**Figure 1:**
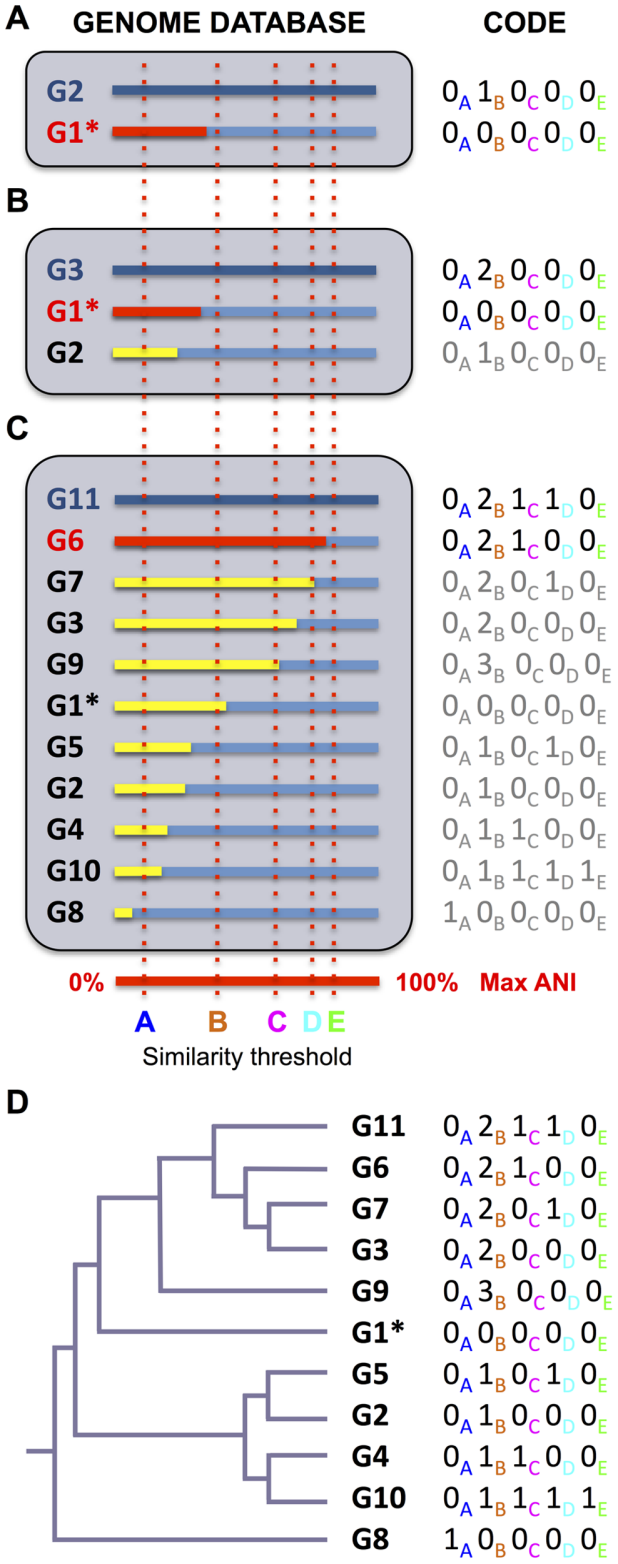
Overview of genome similarity-based code assignment. (**A**) The genome of one organism is chosen as first genome (G1), added to the genome database, and “0” is assigned to all positions in the code (only five positions are shown here for simplicity while codes with 20 positions were used in the examples in Tables 2 to 5). A second genome (G2) is then added to the database and compared to G1. A code is assigned to the organism with genome G2 based on the genome similarity to G1 measured as percentage of average nucleotide identity (ANI). (**B**) The genome of a third organism (G3) is compared to G1 and G2. Since G3 is more similar to G1 than G2, G3 is assigned its code based on its ANI with G1. (**C**) Every new genome that is added to the database will be compared to all genomes already in the database and codes will always be assigned based on the ANI with the most similar genome. (**D**) Since every organism in the database was assigned a code based on genome similarity with the most similar organism already in the database at the time of its addition, all codes reflect the similarity of organisms with each other (as long as their genomes aligned) and thus are an approximation of their phylogenetic relationships (represented by the tree in the figure).

We propose to assign codes as follows (see also Figure 1): (A) The first organism that is submitted for code assignment will be assigned “0” at all positions of its code. (B) The genome of the second organism that is submitted for code assignment is then compared to the genome of the first organism and assigned its code based on its calculated percentage of DNA identity compared to the first organism. (C) The genome of the third organism submitted for code assignment is compared to the genomes of the first two organisms and the organism most similar to the third organism is identified. A code is assigned to the third organism based on its similarity to the organism identified to have the most similar genome to its own. (D) Step (C) is repeated for each additional organism. (E) Because codes are always assigned based on the code of the most similar organism that already has a code, codes will reflect the similarity among all related organisms, i.e., all organisms whose genomes can be aligned to each other.

### Choice of Code Similarity Thresholds

The first important decision to make in the development of the described code system is the choice of similarity thresholds to use at each position of the code in order for codes to reflect biologically relevant relationships between organisms at different levels of similarity: from the family to the genus and species level all the way to relationships between individual organisms. The challenge is that the range of genome similarity values among organisms is very different depending on their evolutionary history. Therefore, codes need to be composed of a large number of positions that reflect many different similarity thresholds. This leads to impractically long codes. However, a simple solution to this problem could be to assign codes with a large number of positions but to use in common parlance only a subset of these positions depending on the group of organisms that is being described. We propose to do this by labeling each position in the code with a different subscript. Table 1 lists the similarity thresholds used for each position in the provisional codes assigned to organisms in the examples shown below and the respective subscript-identifiers. As can be seen from Table 1, intervals between thresholds of adjacent positions decrease from the left to the right of the code. The reason is that the main goal of the proposed codes is to provide a very high-resolution classification and naming system for organisms that are very similar to each other.

**Table 1:** Thresholds of Average Nucleotide Identity (ANI) used for assignment of provisional codes in Tables 2 through 5.

Position label																								
	A	B	C	D	E	F	G	H	I	J	K	L	M	N	O	P	Q	R	S	T	U	V	W	X
ANI %																								
	60	70	80	85	90	95^1^	98	99	99.5	99.6	99.7	99.8	99.9	99.91	99.92	99.93	99.94	99.95	99.96	99.97	99.98	99.99	99.999	99.9999

1 ANI value that approximately corresponds to 70% DDH [15].

### Measurement of Genome Similarities for Genome Code Assignment

To implement genome codes, a method to accurately measure the difference between two genomes as a similarity percentage is needed. Such methods have already been developed and are being used to calculate average nucleotide identity (ANI) values [6], [15], [16] to assign bacteria to named species, thereby replacing experimentally determined DNA-DNA hybridization (DDH) values [4]. ANI calculation is most often based on BLAST [17] and an ANI value of 94% was found to approximately correspond to 70% DDH [15]. Other algorithms that are faster than BLAST have also been used, but they are not suitable for comparing distantly related genomes ([16] and our own experience). Therefore, ANIb (ANI calculated with BLAST) is in our opinion the currently best method to measure the similarity of genomes over a wide range of similarity and was chosen for validating the here described code system. Importantly, when a new genome needs to be assigned a code, ANI will not need to be calculated against all genomes that already have a code. Instead, the group of genomes that is most similar to the new genome could be identified using only a few genes, and then ANI is calculated only against the most similar genomes to precisely identify the most similar genome and the corresponding ANI value.

### Validation of Genome Codes

We validated the here proposed code system using both chromosomal and mitochondrial DNA for different groups of organisms including bacteria, animals, humans, and viruses.

### Bacterial Genome Codes

We first assigned provisional codes to a group of γ proteobacteria and a small group of non-γ proteobacteria for which a tree based on 356 core proteins had been published [18]. Table 2 lists the assigned codes for a selection of taxa (see Table S1 in File S1 for additional taxa, assigned codes, and ANIb values). In this example, code assignment was done in alphabetical order. Table 2 shows that the assigned codes correlate well with known taxonomic groups: (i) all *Enterobacteriaceae* share the same code up to position B (corresponding to the 70% threshold) besides the divergent *Buchnera* species characterized by a very reduced genome size [19]; (ii) the closely related genera *Escherichia* and *Salmonella* share the same code up to position C (corresponding to the 80% threshold); and (iii) the two *Escherichia coli* strains share the same code up to position M (corresponding to the 99.9% threshold). Therefore, not only do the assigned codes correlate well with the named genera and species within the *Enterobacteriaceae*, but they also provide additional information about similarity that is not obvious from the named taxonomic groups. For example, the codes show that bacteria belonging to the genera *Salmonella* and *Escherichia* are closely related, while the genus names do not. However, species belonging to different families within the γ proteobacteria do not share any position in their codes since their genome sequences have diverged to a point that they do not align sufficiently for meaningful code assignment using ANIb.

**Table 2:** Provisional codes assigned to a selection of γ proteobacteria and a small number of non-γ proteobacteria.

Order or family	Species and strain name	Code
Non-gamma	*Acidithiobacillus ferrooxidans* ATCC 23270	0_A_0_B_0_C_0_D_0_E_0_F_0_G_0_H_0_K_0_L_0_M_0_P_0_Q_0_R_
	*Acidithiobacillus ferrooxidans* ATCC 53993	0_A_0_B_0_C_0_D_0_E_0_F_0_G_0_H_0_K_0_L_1_M_0_P_0_Q_0_R_
*Moraxellaceae*	*Acinetobacter* ADP1	1_A_0_B_0_C_0_D_0_E_0_F_0_G_0_H_0_K_0_L_0_M_0_P_0_Q_0_R_
	*Acinetobacter baumannii* ATCC 17978	1_A_0_B_1_C_0_D_0_E_0_F_0_G_0_H_0_K_0_L_0_M_0_P_0_Q_0_R_
Pasteurellales	*Actinobacillus pleuropneumoniae* L20	2_A_0_B_0_C_0_D_0_E_0_F_0_G_0_H_0_K_0_L_0_M_0_P_0_Q_0_R_
	*Actinobacillus succinogenes* 130Z	2_A_0_B_1_C_0_D_0_E_0_F_0_G_0_H_0_K_0_L_0_M_0_P_0_Q_0_R_
	*Haemophilus ducreyi* 35000HP	2_A_0_B_2_C_0_D_0_E_0_F_0_G_0_H_0_K_0_L_0_M_0_P_0_Q_0_R_
	*Haemophilus influenzae* Rd KW20	2_A_0_B_3_C_0_D_0_E_0_F_0_G_0_H_0_K_0_L_0_M_0_P_0_Q_0_R_
	*Haemophilus somnus* 129PT	2_A_0_B_4_C_0_D_0_E_0_F_0_G_0_H_0_K_0_L_0_M_0_P_0_Q_0_R_
	*Mannheimia succiniciproducens* MBEL55E	2_A_0_B_5_C_0_D_0_E_0_F_0_G_0_H_0_K_0_L_0_M_0_P_0_Q_0_R_
	*Pasteurella multocida* Pm70	2_A_0_B_6_C_0_D_0_E_0_F_0_G_0_H_0_K_0_L_0_M_0_P_0_Q_0_R_
*Enterobacteriaceae*	*Buchnera aphidicola* APS	6_A_0_B_0_C_0_D_0_E_0_F_0_G_0_H_0_K_0_L_0_M_0_P_0_Q_0_R_
	*Buchnera aphidicola* Sg	6_A_0_B_1_C_0_D_0_E_0_F_0_G_0_H_0_K_0_L_0_M_0_P_0_Q_0_R_
	*Buchnera aphidicola* Bp	6_A_1_B_0_C_0_D_0_E_0_F_0_G_0_H_0_K_0_L_0_M_0_P_0_Q_0_R_
	*Enterobacter* 638	12_A_0_B_0_C_0_D_0_E_0_F_0_G_0_H_0_K_0_L_0_M_0_P_0_Q_0_R_
	*Escherichia coli* K 12 substr DH10B	12_A_0_B_1_C_0_D_0_E_0_F_0_G_0_H_0_K_0_L_0_M_0_P_0_Q_0_R_
	*Escherichia coli* K 12 substr MG1655	12_A_0_B_1_C_0_D_0_E_0_F_0_G_0_H_0_K_0_L_0_M_0_P_1_Q_0_R_
	*Salmonella enterica* serovar Typhimurium	12_A_0_B_1_C_1_D_0_E_0_F_0_G_0_H_0_I_0_J_0_K_0_L_0_M_0_N_
	*Salmonella enterica* serovar Typhi CT18	12_A_0_B_1_C_1_D_0_E_0_F_0_G_1_H_0_K_0_L_0_M_0_P_0_Q_0_R_
	*Pectobacterium atrosepticum* SCRI1043	12_A_0_B_2_C_0_D_0_E_0_F_0_G_0_H_0_K_0_L_0_M_0_P_0_Q_0_R_
	*Photorhabdus luminescens* laumondii TTO1	12_A_0_B_3_C_0_D_0_E_0_F_0_G_0_H_0_K_0_L_0_M_0_P_0_Q_0_R_
	*Serratia proteamaculans* 568	12_A_0_B_4_C_0_D_0_E_0_F_0_G_0_H_0_K_0_L_0_M_0_P_0_Q_0_R_
	*Sodalis glossinidius* morsitans	12_A_0_B_5_C_0_D_0_E_0_F_0_G_0_H_0_K_0_L_0_M_0_P_0_Q_0_R_
	*Yersinia pestis* CO92	12_A_0_B_6_C_0_D_0_E_0_F_0_G_0_H_0_K_0_L_0_M_0_P_0_Q_0_R_
	*Yersinia pestis* KIM 10	12_A_0_B_6_C_0_D_0_E_0_F_0_G_0_H_0_K_0_L_0_M_0_P_0_Q_1_R_
*Francisellaceae*	*Francisella tularensis* SCHU S4	13_A_0_B_0_C_0_D_0_E_0_F_0_G_0_H_0_K_0_L_0_M_0_P_0_Q_0_R_
Vibrionales	*Photobacterium profundum* SS9	20_A_0_B_0_C_0_D_0_E_0_F_0_G_0_H_0_K_0_L_0_M_0_P_0_Q_0_R_
	*Vibrio fischeri* ES114 58163	20_A_0_B_1_C_0_D_0_E_0_F_0_G_0_H_0_K_0_L_0_M_0_P_0_Q_0_R_
	*Vibrio cholerae* O1 biovar El Tor N16961	20_A_1_B_0_C_0_D_0_E_0_F_0_G_0_H_0_K_0_L_0_M_0_P_0_Q_0_R_
	*Vibrio parahaemolyticus* RIMD 2210633	20_A_1_B_1_C_0_D_0_E_0_F_0_G_0_H_0_K_0_L_0_M_0_P_0_Q_0_R_
	*Vibrio vulnificus* YJ016	20_A_1_B_2_C_0_D_0_E_0_F_0_G_0_H_0_K_0_L_0_M_0_P_0_Q_0_R_
*Pseudomonadaceae*	*Pseudomonas aeruginosa* PAO1	22_A_0_B_0_C_0_D_0_E_0_F_0_G_0_H_0_K_0_L_0_M_0_P_0_Q_0_R_
	*Pseudomonas entomophila* L48	22_A_0_B_1_C_0_D_0_E_0_F_0_G_0_H_0_K_0_L_0_M_0_P_0_Q_0_R_
	*Pseudomonas putida* KT2440	22_A_0_B_1_C_1_D_0_E_0_F_0_G_0_H_0_K_0_L_0_M_0_P_0_Q_0_R_
	*Pseudomonas fluorescens* Pf0 1	22_A_0_B_2_C_0_D_0_E_0_F_0_G_0_H_0_K_0_L_0_M_0_P_0_Q_0_R_
	*Pseudomonas fluorescens* Pf 5	22_A_0_B_2_C_1_D_0_E_0_F_0_G_0_H_0_K_0_L_0_M_0_P_0_Q_0_R_
	*Pseudomonas mendocina* ymp	22_A_0_B_3_C_0_D_0_E_0_F_0_G_0_H_0_K_0_L_0_M_0_P_0_Q_0_R_
	*Pseudomonas stutzeri* A1501	22_A_0_B_4_C_0_D_0_E_0_F_0_G_0_H_0_K_0_L_0_M_0_P_0_Q_0_R_
	*Pseudomonas syringae* pv. tomato DC3000	22_A_0_B_5_C_0_D_0_E_0_F_0_G_0_H_0_K_0_L_0_M_0_P_0_Q_0_R_
*Shewanellaceae*	*Shewanella amazonensis* SB2B	29_A_0_B_0_C_0_D_0_E_0_F_0_G_0_H_0_K_0_L_0_M_0_P_0_Q_0_R_
	*Shewanella baltica* OS155	29_A_0_B_1_C_0_D_0_E_0_F_0_G_0_H_0_K_0_L_0_M_0_P_0_Q_0_R_
	*Shewanella putrefaciens* CN 32	29_A_0_B_1_C_1_D_0_E_0_F_0_G_0_H_0_K_0_L_0_M_0_P_0_Q_0_R_
	*Shewanella frigidimarina* NCIMB 400	29_A_0_B_2_C_0_D_0_E_0_F_0_G_0_H_0_K_0_L_0_M_0_P_0_Q_0_R_
	*Shewanella loihica* PV 4	29_A_0_B_3_C_0_D_0_E_0_F_0_G_0_H_0_K_0_L_0_M_0_P_0_Q_0_R_
	*Shewanella oneidensis* MR 1	29_A_0_B_4_C_0_D_0_E_0_F_0_G_0_H_0_K_0_L_0_M_0_P_0_Q_0_R_
	*Shewanella pealeana* ATCC 700345	29_A_0_B_5_C_0_D_0_E_0_F_0_G_0_H_0_K_0_L_0_M_0_P_0_Q_0_R_
	*Shewanella woodyi* ATCC 51908	29_A_0_B_6_C_0_D_0_E_0_F_0_G_0_H_0_K_0_L_0_M_0_P_0_Q_0_R_
Xanthomonadales	*Stenotrophomonas maltophilia* R551 3	31_A_0_B_0_C_0_D_0_E_0_F_0_G_0_H_0_K_0_L_0_M_0_P_0_Q_0_R_
	*Xanthomonas axonopodis* citrumelo F1	31_A_0_B_1_C_0_D_0_E_0_F_0_G_0_H_0_K_0_L_0_M_0_P_0_Q_0_R_
	*Xanthomonas campestris* ATCC 33913	31_A_0_B_1_C_1_D_0_E_0_F_0_G_0_H_0_K_0_L_0_M_0_P_0_Q_0_R_
	*Xylella fastidiosa* 9a5c	31_A_0_B_2_C_0_D_0_E_0_F_0_G_0_H_0_K_0_L_0_M_0_P_0_Q_0_R_

Code positions from A (60% ANI) to R (99.95% ANI) are shown. See Table S1 in File S1 for codes that were assigned to additional taxa, for ANIb values, and for the percentage of fragments that aligned with the genomes used for code assignment.

Note that in all tables the first organism is always assigned “0” at all positions. However, for permanent code assignment the genomes of all organisms would be submitted to the same database and assigned the next available code independently of their current classification.

The limits of the herein proposed genome code system for bacterial isolates belonging to the same named species were explored next. *Bacillus anthracis* was chosen, because it is a typical example of a species characterized by very little sequence variation [7] and genome sequences of many strains belonging to this species are publicly available. Since horizontally acquired genomic regions were found to distort code assignment for *B. anthracis* (data not shown), predicted horizontally acquired genomic regions were excluded during the calculation of ANIb (see methods section below). Using this modification, we were able to assign codes to *B. anthracis* isolates (Table 3 and Table S2 in File S1) that reveal meaningful subgroups within this species; for example, one subgroup comprises most isolates of the Ames strain used in the 2001 bioterrorist attacks [20]. Therefore, the here described code system could provide the means to systematically name strains within *B. anthracis*, for which no systematic intra-species classification and naming system currently exists. Of course, we would expect further improvements and modifications to the calculation of genome similarity and code assignment before assigning permanent genome codes widely. The purpose of this example is simply to show the potential of genome codes but not to assign final permanent codes.

**Table 3:** Provisional codes assigned to *Bacillus anthracis* strains.

*Bacillus anthracis* strains	Code
A0174	0_V_0_W_0_X_
A0193	0_V_1_W_0_X_
Western North America USA6153	0_V_2_W_0_X_
Tsiankovskii I	0_V_3_W_0_X_
A0389	1_V_0_W_0_X_
Ames	1_V_1_W_0_X_
Ames Ancestor	1_V_1_W_1_X_
A0248	1_V_1_W_1_X_
Australia 94	1_V_2_W_0_X_
Sterne	1_V_3_W_0_X_
A0442	2_V_0_W_0_X_
Kruger B	2_V_1_W_0_X_
A0465	3_V_0_W_0_X_
CNEVA 9066	3_V_1_W_0_X_
A0488	4_V_0_W_0_X_
CDC 684	4_V_1_W_0_X_
Vollum	4_V_2_W_0_X_
A1055	5_V_0_W_0_X_
A2012	6_V_0_W_0_X_
H9401	7_V_0_W_0_X_

Code positions from V (99.99% ANI) to X (99.9999% ANI) are shown. See Table S2 in File S1 for ANIb values and for the percentage of fragments that aligned with the genomes used for code assignment.

### Mitochondrial Codes for Animal Species and Human Populations

Phylogeny based on mitochondrial genomes of sexually reproducing eukaryotes is a good proxy of phylogenetic relationships based on the maternal lineage [21]. We thus used mitochondrial genomes of a wide range of eukaryotes to determine if the proposed genome code system could reflect known phylogenetic relationships within eukaryotes (examples of assigned codes are shown in Table 4 and a complete list of assigned codes including ANIb values are listed in Table S3 in File S1). It can be seen that, for example, members of the phylum chordata share the same code at position A, mammals share the same code up to position B, and primates share the same code up to position C. Therefore, there is a good correspondence between mitochondrial genome codes and taxonomic classes within the animal kingdom.

**Table 4:** Examples of provisional mitochondrial codes assigned to members of the phylum chordata.

Class/order/family, Species		Common name	Code
Amphibia/Anura/Ranidae			
	*Pelophylax nigromaculatus*	Dark-spotted frog	1_A_1_B_76_C_0_D_0_E_0_F_0_G_0_H_
Mammalia/Rodentia/Muridae			
	*Mus musculus*	House mouse	1_A_0_B_28_C_0_D_0_E_0_F_0_G_0_H_
	*Rattus norvegicus*	Brown rat	1_A_0_B_28_C_1_D_0_E_0_F_0_G_0_H_
Mammalia/Primates/Hominidae			
	*Gorilla gorilla*	Gorilla	1_A_0_B_18_C_0_D_0_E_0_F_0_G_0_H_
	*Homo sapiens*	Human	1_A_0_B_18_C_0_D_1_E_0_F_0_G_0_H_
	*Pan paniscus*	Bonobo	1_A_0_B_18_C_0_D_1_E_1_F_0_G_0_H_
	*Pan troglodytes*	Common Chimpanzee	1_A_0_B_18_C_0_D_1_E_1_F_1_G_0_H_
	*Pongo abelii*	Sumatran Orangutan	1_A_0_B_18_C_0_D_2_E_0_F_0_G_0_H_
	*Pongo pygmaeus*	Bornean orangutan	1_A_0_B_18_C_0_D_2_E_1_F_0_G_0_H_
Mammalia/Primates/Hylobatidae			
	*Hylobates lar*	Lar gibbon	1_A_0_B_18_C_1_D_0_E_0_F_0_G_0_H_

Code positions from A (60% ANI) to H (99% ANI) are shown. See Table S3 in File S1 for codes, ANIb values, and percentage of fragments that aligned with the genomes used for code assignment for 466 mitochondria.

We then assigned provisional codes to 902 individual mitochondrial human genomes [22] (Table S4 in File S1) revealing that mitochondrial codes can distinguish between human populations and reflect groupings similar to currently used haplogroups. Mitochondrial codes could thus be part of unique identifiers assigned to individual human beings, whereby mitochondrial codes would largely reflect ancestry based on the maternal lineage. Y-chromosome codes could provide additional resolution and information about the paternal lineage for males. Autosomal codes would need to be adapted to reflect similarity between diploid genomes. Although we do not expect that autosomal codes would reflect ancestry, highly similar autosomal codes could still be informative of close family ties and could provide informative unique identifiers for individual human beings.

### Viral Genome Codes

Finally, we validated the proposed code system for viruses using as example isolates of the Foot and Mouth Disease virus (FMDV) from the 2001 UK outbreak [23] and from India [24]. Codes assigned to isolates from the UK and from India are clearly distinct (Table 5 and Table S5 in File S1). Moreover, comparison of codes among the UK isolates with the phylogeography of FMDV during the 2001 UK outbreak [23] reveals that codes are informative of transmission events and can thus provide meaningful labels for individual viral isolates during an epidemic.

**Table 5:** Examples of provisional mitochondrial codes assigned to Foot and Mouth Disease Viruses.

Country of isolation			
Accession #		Code	
UK			
	DQ404158		0_C_ 0_E_ 0_F_ 0_G_ 0_H_ 0_I_ 0_J_ 0_K_ 0_L_ 0_M_ 0_R_ 0_X_
	DQ404159		0_C_ 0_E_ 0_F_ 0_G_ 0_H_ 0_I_ 0_J_ 0_K_ 1_L_ 0_M_ 0_R_ 0_X_
	DQ404160		0_C_ 0_E_ 0_F_ 0_G_ 0_H_ 0_I_ 0_J_ 0_K_ 1_L_ 1_M_ 0_R_ 0_X_
	DQ404161		0_C_ 0_E_ 0_F_ 0_G_ 0_H_ 0_I_ 0_J_ 1_K_ 0_L_ 0_M_ 0_R_ 0_X_
	DQ404162		0_C_ 0_E_ 0_F_ 0_G_ 0_H_ 1_I_ 0_J_ 0_K_ 0_L_ 0_M_ 0_R_ 0_X_
	DQ404163		0_C_ 0_E_ 0_F_ 0_G_ 0_H_ 2_I_ 0_J_ 0_K_ 0_L_ 0_M_ 0_R_ 0_X_
	DQ404164		0_C_ 0_E_ 0_F_ 0_G_ 0_H_ 3_I_ 0_J_ 0_K_ 0_L_ 0_M_ 0_R_ 0_X_
	DQ404165		0_C_ 0_E_ 0_F_ 0_G_ 0_H_ 3_I_ 1_J_ 0_K_ 0_L_ 0_M_ 0_R_ 0_X_
	DQ404166		0_C_ 0_E_ 0_F_ 0_G_ 0_H_ 3_I_ 1_J_ 0_K_ 0_L_ 0_M_ 0_R_ 1_X_
	DQ404167		0_C_ 0_E_ 0_F_ 0_G_ 0_H_ 3_I_ 1_J_ 0_K_ 0_L_ 0_M_ 1_R_ 0_X_
	DQ404168		0_C_ 0_E_ 0_F_ 0_G_ 0_H_ 3_I_ 1_J_ 0_K_ 2_L_ 0_M_ 0_R_ 0_X_
	DQ404169		0_C_ 0_E_ 0_F_ 0_G_ 0_H_ 3_I_ 1_J_ 0_K_ 3_L_ 0_M_ 0_R_ 0_X_
	DQ404170		0_C_ 0_E_ 0_F_ 0_G_ 0_H_ 3_I_ 1_J_ 0_K_ 0_L_ 1_M_ 0_R_ 0_X_
	DQ404171		0_C_ 0_E_ 0_F_ 0_G_ 0_H_ 3_I_ 1_J_ 0_K_ 0_L_ 2_M_ 0_R_ 0_X_
	DQ404172		0_C_ 0_E_ 0_F_ 0_G_ 0_H_ 3_I_ 1_J_ 0_K_ 0_L_ 3_M_ 0_R_ 0_X_
	DQ404173		0_C_ 0_E_ 0_F_ 0_G_ 0_H_ 3_I_ 1_J_ 0_K_ 0_L_ 3_M_ 0_R_ 1_X_
	DQ404174		0_C_ 0_E_ 0_F_ 0_G_ 0_H_ 3_I_ 1_J_ 0_K_ 0_L_ 3_M_ 1_R_ 0_X_
	DQ404175		0_C_ 0_E_ 0_F_ 0_G_ 0_H_ 3_I_ 1_J_ 0_K_ 0_L_ 3_M_ 0_R_ 2_X_
	DQ404176		0_C_ 0_E_ 0_F_ 0_G_ 0_H_ 3_I_ 1_J_ 0_K_ 0_L_ 3_M_ 2_R_ 0_X_
	DQ404177		0_C_ 0_E_ 0_F_ 0_G_ 0_H_ 3_I_ 1_J_ 0_K_ 0_L_ 3_M_ 2_R_ 1_X_
	DQ404178		0_C_ 0_E_ 0_F_ 0_G_ 0_H_ 3_I_ 1_J_ 0_K_ 0_L_ 3_M_ 2_R_ 2_X_
	DQ404179		0_C_ 0_E_ 0_F_ 0_G_ 0_H_ 3_I_ 1_J_ 0_K_ 0_L_ 3_M_ 2_R_ 3_X_
	DQ404180		0_C_ 0_E_ 0_F_ 0_G_ 0_H_ 3_I_ 1_J_ 0_K_ 0_L_ 3_M_ 3_R_ 0_X_
India			
	HQ832576		0_C_ 1_E_ 0_F_ 0_G_ 0_H_ 0_I_ 0_J_ 0_K_ 0_L_ 0_M_ 0_R_ 0_X_
	HQ832577		0_C_ 1_E_ 1_F_ 0_G_ 0_H_ 0_I_ 0_J_ 0_K_ 0_L_ 0_M_ 0_R_ 0_X_
	HQ832578		0_C_ 1_E_ 2_F_ 0_G_ 0_H_ 0_I_ 0_J_ 0_K_ 0_L_ 0_M_ 0_R_ 0_X_
	HQ832579		0_C_ 1_E_ 2_F_ 0_G_ 1_H_ 0_I_ 0_J_ 0_K_ 0_L_ 0_M_ 0_R_ 0_X_
	HQ832580		0_C_ 1_E_ 2_F_ 0_G_ 2_H_ 0_I_ 0_J_ 0_K_ 0_L_ 0_M_ 0_R_ 0_X_
	HQ832581		0_C_ 1_E_ 2_F_ 0_G_ 3_H_ 0_I_ 0_J_ 0_K_ 0_L_ 0_M_ 0_R_ 0_X_
	HQ832582		0_C_ 1_E_ 2_F_ 1_G_ 0_H_ 0_I_ 0_J_ 0_K_ 0_L_ 0_M_ 0_R_ 0_X_
	HQ832583		0_C_ 1_E_ 2_F_ 0_G_ 4_H_ 0_I_ 0_J_ 0_K_ 0_L_ 0_M_ 0_R_ 0_X_
	HQ832584		0_C_ 1_E_ 3_F_ 0_G_ 0_H_ 0_I_ 0_J_ 0_K_ 0_L_ 0_M_ 0_R_ 0_X_
	HQ832585		0_C_ 1_E_ 4_F_ 0_G_ 0_H_ 0_I_ 0_J_ 0_K_ 0_L_ 0_M_ 0_R_ 0_X_
	HQ832586		0_C_ 1_E_ 5_F_ 0_G_ 0_H_ 0_I_ 0_J_ 0_K_ 0_L_ 0_M_ 0_R_ 0_X_
	HQ832587		0_C_ 1_E_ 6_F_ 0_G_ 0_H_ 0_I_ 0_J_ 0_K_ 0_L_ 0_M_ 0_R_ 0_X_
	HQ832588		0_C_ 1_E_ 7_F_ 0_G_ 0_H_ 0_I_ 0_J_ 0_K_ 0_L_ 0_M_ 0_R_ 0_X_
	HQ832589		0_C_ 1_E_ 8_F_ 0_G_ 0_H_ 0_I_ 0_J_ 0_K_ 0_L_ 0_M_ 0_R_ 0_X_
	HQ832590		0_C_ 1_E_ 9_F_ 0_G_ 0_H_ 0_I_ 0_J_ 0_K_ 0_L_ 0_M_ 0_R_ 0_X_
	HQ832591		0_C_ 1_E_ 9_F_ 1_G_ 0_H_ 0_I_ 0_J_ 0_K_ 0_L_ 0_M_ 0_R_ 0_X_
	HQ832592		0_C_ 1_E_ 9_F_ 2_G_ 0_H_ 0_I_ 0_J_ 0_K_ 0_L_ 0_M_ 0_R_ 0_X_

Code positions ranging from C (80% ANI) to X (99.9999% ANI) are shown. See Table S5 in File S1 for codes, ANIb values, and percentage of fragments that aligned with the genomes used for code assignment.

### Influence of the Order of Code Assignment on Similarity of Codes between Organisms

Since we propose to assign codes to organisms sequentially in the order in which their genomes are submitted for code assignment, it was important to determine the effect of the order of code assignment on the similarity of codes between organisms. This was done by assigning codes to the γ proteobacteria from Table 2 in 100 random orders. We found that on average the last common position shared between pairs of organisms only changed in 3.02 runs out of 100 runs and never changed by more than one code position. Therefore, the order of code assignment can slightly change the similarity of codes between organisms, but, because the result is only a shift of the last shared position, codes can be expected to reflect similarity between organisms independently of the order in which they are assigned.

## Genome Codes could Complement Current Biological Classification

### Genome Codes could Provide a General Intraspecies Classification and Naming System

We have shown with the provided examples that genome codes can reflect known similarity and relationships between organisms from the family level all the way to the single genetic lineage or organism. Therefore, genome codes could provide a new approach to classify and name life beyond the species with the single organism as ultimate unit. Genome codes could thus finally provide one general intraspecies classification and naming system for all life, addressing one of the main limitations of current biological classification: the use of the species as basic unit.

### Species are Predictive of Phenotype and Ancestry; Genome Codes are Predictive of Genome Similarity

Genome codes should be considered a classification and naming system that complements and extends - but does not replace - existing biological classification.

In fact, the first important difference between named species and genome codes is that named species are associated with phenotypical descriptions. Therefore, species names are predictive of at least some of the phenotypic characteristics of the organisms that are assigned to a particular species. On the other hand, as we pointed out above, species are not predictive of the genetic diversity of organisms they encompass: two organisms that belong to the same species may be very similar or quite different from each other. The proposed genome codes, however, are not associated with phenotypic descriptions of organisms but are highly predictive of the similarity between organisms; independently of the species to which two organisms belong, codes will express their genome sequence similarity to each other.

Secondly, current biological taxonomy and nomenclature, in particular phylogenetic nomenclature [9], is based on phylogeny. However, phylogenetic relationships between individual organisms belonging to the same species are ambiguous and heavily depend on the organisms that are sampled and the algorithms and genetic markers that are employed. Also, recombination makes it sometimes impossible to decide which phylogeny represents the true evolutionary history of closely related taxa [25]. Also, codes based on phylogeny would need to be revised when new related genomes are added and would need to be assigned based on many genomes instead of only the most similar genome requiring much higher computing power. In contrast, genome codes would not require calculation of ANI compared to all genomes in a database. The group of most similar genomes could be easily determined based on one - or a small number of – genes. ANI would then only be calculated for the most similar genomes to identify precisely the most similar genome based on which the code would be assigned to the new genome.

Therefore, a phylogenetic approach is not advantageous over a simple genome similarity-based approach and could not provide unique and stable identifiers for individual organisms that can be assigned as soon as a new genome sequence becomes available. This is instead the case with the genome codes proposed herein, which can be immediately assigned to each new genome sequence simply based on similarity to the most similar organism with a previously assigned code.

In conclusion, genome codes would not replace - but would complement - Linnean and phylogenetic classification and nomenclature and genome codes would be suited for all situations when fast and precise classification, identification, and naming of individual organisms are important.

### Species Description and Delimitations Change Over Time While Genome-codes are Stable

Finally, because species are expected to be predictive of the phenotypical characteristics of the organisms that belong to them and should reflect to our best knowledge phylogenetic relationships, species are necessarily subject to change. Species need to be revised upon additional characterization of the organisms belonging to a species or after discovery of new diversity within a described species or close to a described species. As pointed out above, this can create dangerous confusion. Since genome codes would be assigned to individual organisms instead of species and would not be expected to be predictive of anything besides genome similarity, they would not need to be revised. Therefore, codes would not change when new diversity is discovered providing a third essential advantage over current biological classification (at the expense of course of not being predictive of anything besides genome similarity).

## Inherent Properties of Genome Codes

### Link between Accuracy of Codes and Genome Sequence Quality

Because code assignment would be based on genome sequences, errors in genome sequences would be reflected in assigned genome codes. For example, if a genome sequence contains many errors, the code of the organism would be more different from the most similar genome that already has a code than it should. Therefore, it would be important that permanent codes would only be assigned based on complete and high quality genome sequences. Alternatively, organisms with low quality genome sequences or only partial genome sequences could simply be assigned codes up to a position with a relatively low similarity threshold. The remaining code positions would be assigned only after high quality genome sequences become available for these organisms.

### Correlation between Phylogeny, Genome Similarity, and Code Similarity

The percentage DNA identity threshold of the last position shared between genome codes of two organisms would not correspond exactly to the percentage of DNA identity between the two organisms’ genomes. In fact, two organisms that share the same code up to a certain position, for example position H corresponding to 99% similarity, might actually be slightly less identical to each other than 99%. The reason is that sharing the same code up to position H in the proposed system would mean that for each of the two organisms there is at least one other organism that is at least 99% identical and that has the same code at position H. For example, if two organisms are between 98% and 99% identical to each other but more than 99% identical to a third organism, then they would have the same code up to position H if they were assigned their codes after the third organism was assigned its code. However, they would have the same code up to position G if they were assigned codes before the third organism was assigned its code. Thus, the order of code assignment can slightly change the similarity of codes between organisms (for example, on average in 3 runs out of 100 runs for the γ proteobacteria listed in Table 2 as explained above). Therefore, two organisms that have the same code up to a certain position would have genomes with percentage DNA identity similar (but not identical) to the threshold of that position.

While we found that codes based on genome similarity largely correspond to known taxonomic classes and reflect known phylogenetic relationships in our examples, we do not claim that codes generally reflect evolutionary relationships. Obviously, phylogeny-based codes would better reflect evolutionary relationships than genome similarity-based codes. However, it would be impossible to assign phylogeny-based codes one genome at the time and such codes would need to be revised whenever the addition of a new genome sequence changes the reconstructed evolutionary history of a group of organisms. Therefore, phylogeny-based codes could not be assigned to an organism automatically as soon as its genome becomes available and they would not be stable. Phylogeny-based codes would thus not be adequate for the applications we envision for genome codes (see below).

### Recombination and Genome Codes

Horizontal transfer of DNA (or recombination) between bacterial or viral strains and acquisition or loss of a plasmid in the case of bacteria will affect the overall percentage of DNA identity between genomes, in particular, if the strains have an overall high similarity. Therefore, using whole genomes for code assignment for *B. anthracis* gave rise to codes that did not reflect the relationship between strains based on their core genome. For example, we found that codes assigned to isolates derived from the Ames strain and codes assigned to more distantly related isolates did not reflect known relationships. By eliminating all regions of the *B. anthracis* genome that deviated significantly from overall genome similarity, we obtained codes that closely reflected the phylogeny of strains. Therefore, for applications in molecular disease epidemiology we think that it will be important to assign codes based only on vertically inherited core genomes so that isolates connected epidemiologically have codes that are more similar to each other than isolates that belong to separate outbreaks. However, one could argue that it is important to include the most variable genomic regions in code assignment since they are important to distinguish between outbreak strains with different antibiotic resistance genes for example.

In the case of highly recombining viruses, bacteria, and sexually reproducing organisms, it will usually not be possible to eliminate recombining regions before calculation of DNA identity because recombination is too widespread. In this case, genome codes will necessarily be strongly affected by recombination. However, in such cases the relationships between organisms are in fact ambiguous, and codes would simply reflect this ambiguity. But even in the cases when codes were not to clearly reflect genome similarity, codes would still be useful as unique identifiers to name individual isolates or organisms in a systematic way.

### Distantly Related Organisms have Completely Different Codes

Because animals are much more closely related to each other than bacteria, mitochondrial genomes of all members of the chordata can be aligned with each other using BLAST and thus all chordata mitochondria share the same code at position A. On the other hand, genomes of bacteria belonging to different families within the γ proteobacteria are only distantly related, cannot be significantly aligned, and thus do not share any code positions. However, future improvements to the measurement of genome similarity may make it possible to assign codes at additional positions with lower similarity thresholds to label, for example, all members of the γ proteobacteria with a shared code at the left-most position. This could, for example, be done employing average amino acid identity (AAI) [26] for the left-most positions in the code.

### Applications of Genome Codes in Biological Sciences and Beyond

**Figure 2:**
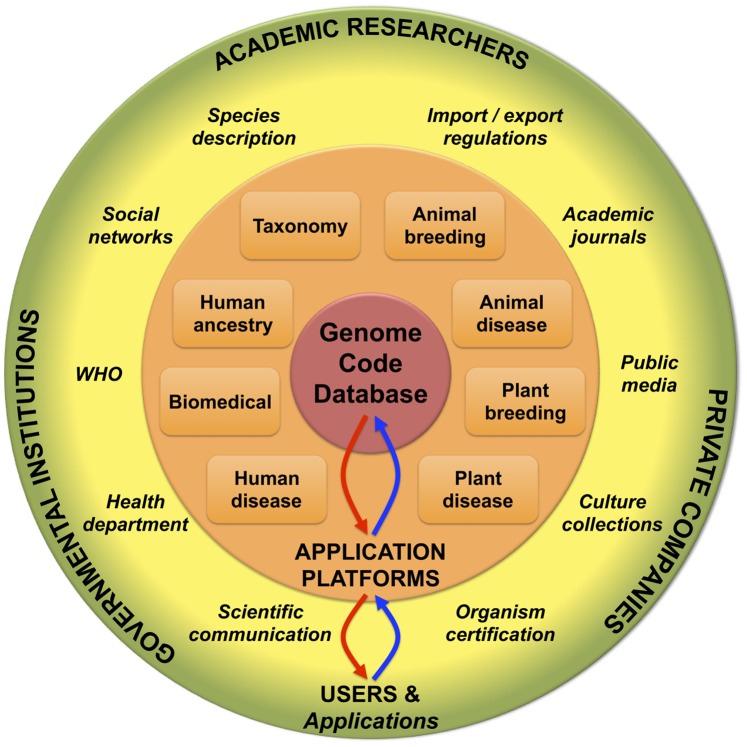
Applications of genome similarity-based codes in Science and Society. Each user who wanted to obtain a code for an organism would submit a genome sequence to a platform associated with a specific application. Each application platform could submit genomes to a central code database for unique code assignment. Codes would then be returned to the application platform, in which codes could be stored instead of entire genome sequences. Each platform would also store application-specific metadata associated with each code while the central code database would mainly store genomes and associated codes. Genome submissions are symbolized by blue arrows; code assignments are symbolized by red arrows.

Genome codes could provide the means for academic researchers to communicate about any individual organism without ambiguity, but codes could also play a central role in many applications that go beyond basic research and that have social benefits as well. Figure 2 summarizes the central role that we predict for genome codes in biological sciences and beyond.

### Genome Codes for Communication about Individual Organisms without Ambiguity

In all academic journals, each species is referred to by its common name and by its scientific binomial in order to clearly identify it. Similarly, genome codes could be used when describing any individual organism or virus in a journal article. Genome sequencing has already become so common that many organisms described in journal articles have already been sequenced. Therefore, with the introduction of genome codes, these organisms could be precisely identified in each journal article with their code instead of reporting the species name only.

### Genome Codes for Species Descriptions and Species Revisions

As pointed out above, different species can be of very different diversity, and species names are thus not predictive of the diversity of organisms that belong to a certain species. Including genome codes in species descriptions could alleviate this problem. For example, the species description of *B. antracis* and *E. coli* could be augmented with the genome code positions shared by all *B. anthracis* and all *E. coli* strains, respectively. Since *B. anthracis* strains are much more similar to each other than *E. coli* strains, the code positions describing the two species would reflect that. Also, the number of different values at each position of the codes associated with a certain species at the time of its description could be included in the species description as a measure of its known diversity.

Moreover, if species descriptions are revised because of the discovery of new diversity or identification of differences between organisms previously lumped into the same species, genome codes could provide the stability and continuity to alleviate the unavoidable confusion whenever species revisions and/or name changes are made. For example, if a species is divided into two newly described and named species, the codes of the new species would fall within the range of codes associated with the previous species, making it easy to immediately see that the two new species correspond to two groups contained within the previous species. Therefore, the stability of codes could become instrumental in species description and revisions.

### Genome Codes as Unique Identifiers to Communicate about Emerging Pathogens and any other Newly Discovered Organisms

Since genome codes could be assigned automatically to any genome without having to make a decision about species assignment and/or without describing and naming new species, codes could be used to name organisms as soon as they are isolated for the first time and their genomes have been sequenced. This is particularly important when a new pathogen emerges. It may take time to describe a new pathogen and decide if it is a new species or if it is simply a new epidemic clone of an already named species. Also, different scientists or health officials in different countries may give the same pathogen strain different names. However, if genome sequences of all isolates were submitted to the same database for code assignment, everyone could refer to the new pathogen with the code positions that are shared among all isolates. This would make it possible to communicate globally about a new pathogen with no confusion. The same is true for non-pathogenic organisms identified in biodiversity surveys. Therefore, genome codes could provide the means to name any newly identified organism immediately after its genome is sequenced, long before it is described as a named species.

But genome codes would also be extremely useful when communicating about any strain of an already described pathogen in the case of natural disease outbreaks or bioterrorist attacks. For example, the *B. anthracis* strain used in the bioterrorist attacks of 2001 is called the “Ames” strain based on the return address on an envelope in which it was originally sent from Texas to USAMRIID. Other *B. anthracis* strains have other colloquial names that do not reflect their relationship with the Ames strain. However, after assigning genome codes to each strain, the strains could be referred to by the code positions that distinguish them from each other as shown in Table 3. The code of each strain would immediately reveal its similarity to all other strains, greatly facilitating the communication about outbreak strains in disease control and prevention and microbial forensics.

### Genome Codes for Certification of animal breeds and plant cultivars

The ability of genome codes to provide the means to systematically name organisms within species would also be of great utility for eukaryotes, for example, when describing the immense diversity of insects or when discriminating cryptic species. Additionally, codes could also be useful in more practical applications that go beyond basic scientific research. For example, animal breeds or plant cultivars could be identified with a genome code (or a range of codes) creating the means to certify individual animals or plants as belonging to a certain breed or cultivar. For example, a specific dog breed could be associated with a certain range of genome codes and a particular dog could be certified as belonging to a breed because its individual code falls within the code range of the breed.

### Reconstruction of Human Ancestry with Genome Codes

Genome codes could also be used in human ancestry to reflect relationships between individual human beings. Each person who has his or her genome sequenced could get an autosomal genome code and a mitochondrial code, and males could obtain a Y-chromosome code as well. Since mitochondrial and Y chromosomes are not subject to recombination, the respective codes would accurately reflect the similarity to everybody else whose genome was sequenced and obtained a code. Comparing codes could thus make it very easy for people to determine how closely related they are to each other and compare each other’s ancestry.

## Conclusions

Genome sequencing offers us the opportunity today to precisely identify any individual bacterial clone or virus or individual plant, animal, or human. However, so far we have not been able to take full advantage of the precision of genome sequencing for classification and naming because the current biological classification and naming system is based on the species as the basic unit. A genome code system like the one proposed herein could fill that need; it would provide the means to use genome sequencing to identify and systematically name any individual life form. Therefore, applying genome codes would not only be advantageous in basic research but it would be instrumental in all areas where precise identification and naming of organisms is important, from public health to animal and plant breeding to biodiversity surveys, forensics, and ancestry research.

## Materials and Methods

All genomes were downloaded from NCBI. After the graphical user interface was removed from JSpecies [16] the core of this program was integrated into a custom pipeline programmed in Java to (i) perform “all against all” pairwise genome similarity calculations, (ii) sequentially determine the most similar genome for each genome, and (iii) assign codes.

### “All Against All” Genome Similarity Calculations

The first step performed by JSpecies [16] is to divide a genome into 1020bp-long consecutive fragments. For any two genomes, the fragments of these genomes are compared to each other using BLASTn and their DNA similarity is reported. JSpecies then selects those fragments of the query genome that align with the subject genome over 70% of their length and with 30% overall sequence identity. The number of fragments that satisfy these two criteria divided by the total number of fragments of the query genome is called the “percentage of aligned fragments” from here on. Percentage DNA identity values of the selected fragments are then averaged to calculate the Average Nucleotide Identity (ANI) between the corresponding genomes. For the first step of our pipeline, we wrote a script that ran JSpecies [16] in sequence using as input all pairwise combinations of genomes in a selected group, for example the γ proteobacteria. The “percentage of aligned fragments” and ANI values from all runs were automatically saved in a single file.

### Sequential Determination of the Most Similar Genome

The ANI and “percentage of aligned fragments” values from the obtained file were then used as input for sequential identification of the most similar genome for each genome in the group using a custom script. For example, for code assignment in alphabetical order, the first genome of a group was compared to itself, the second genome was compared to the first genome, and the third genome was compared to the first and the second genome, etc. If 20% or more of the query genome fragments aligned with one or more of the subject genomes, the genome with the highest ANI was selected among these genomes as the most similar genome. We chose 20% as the cut-off because we found that ANIb based on less than 20% of the aligned fragments had no correlation with phylogeny. If there was not a single genome with which more than 20% of the query genome fragments aligned, the genome with the highest ANI was selected as the most similar genome independently of the “percentage of aligned fragments” value. However, in this case, the genome was not used as the basis for code assignment in the next step (see below). A table listing for each genome the most similar genome and the associated ANI and “percentage of aligned fragments” values was saved in a single file.

### Code Assignment

The above file was then used as input for code assignment. The value “0” was assigned to the first genome in alphabetical order at all positions of the code (y_A_,y_B_,y_C_,y_C_, …,y_X_; where each “y” stands for “position” and each subscript corresponds to one of the 24 levels of similarity). To all other genomes, a code was assigned one by one based on the most similar genome of all the genomes that were already assigned a code (as exemplified in Figure 1). If the percentage of aligned fragments was higher than 20, the following *if* statement was executed for each threshold (x_A_, x_B_, x_C_, x_D_, …, x_X_) and position in the code (y_A_,y_B_,y_C_,y_D_, …,y_X_): *if* ANI is higher than cutoff x at position y, *then* assign the same number as the most similar genome in position y, *else* assign next higher number to position y and 0 to all following positions. On the other hand, if the “percentage of aligned fragments” value was lower than 20, the genome was simply assigned the next higher number at the first position and 0 at all consecutive positions.

### Modification of JSpecies to Limit ANI Calculation to Predicted Core Genome

To limit calculation of ANI for *B. anthracis* as much as possible to the vertically inherited core genome (i.e., excluding predicted horizontally transferred regions), a second filtration step was applied to the fragments that had passed the filtration step already implemented in JSpecies (i.e., alignment over 70% of fragment length and with 30 % overall sequence identity with subject genome). To implement this second filtration step, the median % DNA identity was determined for all fragments that had passed the first filtration step and only those fragments with a % DNA identity within a 0.1 interval of the median of these fragments were used for calculation of ANI.

## Supporting Information

File S1 **Tables S1–S5, Report for each genome used in this article the most similar genome based on which the provisional genome code was assigned, the ANIb% value, the % of aligned fragments, and the assigned genome code.**
(PDF)
