# Supplementary material for: A System to Automatically Classify and Name Any Individual Genome-Sequenced Organism Independently of Current Biological Classification and Nomenclature
Source: PLoS One. 2014 Feb 21;9(2):e89142. doi: 10.1371/journal.pone.0089142 (PMC3931686; doi:10.1371/journal.pone.0089142)
Supplement: File S1 — Tables S1–S5, Report for each genome used in this article the most similar genome based on which the provisional genome code was assigned, the ANIb% value, the % of aligned fragments, and the assigned genome code. (PDF) [file pone.0089142.s001.pdf]

Table S1. Gammaproteobacteria (plus some non-gamma proteobacteria)				code													
query	subject (most similar genome to query genome considering only genomes in rows above the query genome)	ANIb %	% of aligned fragments	A 60%	B 70%	C 80%	D 85%	E 90%	F 95%	G 98%	H 99%	K 99.7%	L 99.8%	M 99.9%	P 99.93%	Q 99.94%	R 99.95%
Acidithiobacillus_ferrooxidans_ATCC_232	Acidithiobacillus_ferrooxidans_ATCC_	100	99.97	0	0	0	0	0	0	0	0	0	0	0	0	0	0
Acidithiobacillus_ferrooxidans_ATCC_539	Acidithiobacillus_ferrooxidans_ATCC_	99.75	92.93	0	0	0	0	0	0	0	0	0	0	1	0	0	0
Acinetobacter_ADP1_uid61597	Acidithiobacillus_ferrooxidans_ATCC_	65.83	3.43	1	0	0	0	0	0	0	0	0	0	0	0	0	0
Acinetobacter_baumannii_ATCC_17978_	Acinetobacter_ADP1_uid61597	74.23	52.36	1	0	1	0	0	0	0	0	0	0	0	0	0	0
Coxiella_burnetii_RSA_493_uid57631	Colwellia_psychrerythraea_34H_uid5	63.47	7.97	10	0	0	0	0	0	0	0	0	0	0	0	0	0
Dichelobacter_nodosus_VCS1703A_uid57	Buchnera_aphidicola_Sg__Schizaphis_	65.24	5.8	11	0	0	0	0	0	0	0	0	0	0	0	0	0
Enterobacter_638_uid58727	Aeromonas_hydrophila_ATCC_7966_	68.37	19.93	12	0	0	0	0	0	0	0	0	0	0	0	0	0
Escherichia_coli_K_12_substr__DH10B_u	Enterobacter_638_uid58727	78.09	58.39	12	0	1	0	0	0	0	0	0	0	0	0	0	0
Escherichia_coli_K_12_substr__MG1655_	Escherichia_coli_K_12_substr__DH10	99.93	97.16	12	0	1	0	0	0	0	0	0	0	0	0	1	0
Salmonella_enterica_serovar_Typhimuri	Escherichia_coli_K_12_substr__MG1	80.44	60.45	12	0	1	1	0	0	0	0	0	0	0	0	0	0
Salmonella_enterica_serovar_Typhi_CT1	Salmonella_enterica_serovar_Typhim	98.22	82.94	12	0	1	1	0	0	0	1	0	0	0	0	0	0
Pectobacterium_atrosepticum_SCRI1043_	Escherichia_coli_K_12_substr__DH10	72.62	32.45	12	0	2	0	0	0	0	0	0	0	0	0	0	0
Photorhabdus_luminescens_laumondii_T	Escherichia_coli_K_12_substr__DH10	70.45	22.75	12	0	3	0	0	0	0	0	0	0	0	0	0	0
Serratia_proteamaculans_568_uid58725	Pectobacterium_atrosepticum_SCRI1	73.72	36.8	12	0	4	0	0	0	0	0	0	0	0	0	0	0
Sodalis_glossinidius__morsitans__uid585	Serratia_proteamaculans_568_uid58	73.15	32.04	12	0	5	0	0	0	0	0	0	0	0	0	0	0
Yersinia_pestis_CO92_uid57621	Serratia_proteamaculans_568_uid58	74.83	40.87	12	0	6	0	0	0	0	0	0	0	0	0	0	0
Yersinia_pestis_KIM_10_uid57875	Yersinia_pestis_CO92_uid57621	99.94	99.02	12	0	6	0	0	0	0	0	0	0	0	0	0	1
Francisella_tularensis_SCHU_S4_uid5758	Chromohalobacter_salexigens_DSM_	65.97	2.21	13	0	0	0	0	0	0	0	0	0	0	0	0	0
Hahella_chejuensis_KCTC_2396_uid5848	Alcanivorax_borkumensis_SK2_uid58	66.34	7.75	14	0	0	0	0	0	0	0	0	0	0	0	0	0
Idiomarina_loihiensis_L2TR_uid58087	Colwellia_psychrerythraea_34H_uid5	66.47	17.82	15	0	0	0	0	0	0	0	0	0	0	0	0	0
Marinobacter_aquaeolei_VT8_uid59419	Alcanivorax_borkumensis_SK2_uid58	68.15	14.38	16	0	0	0	0	0	0	0	0	0	0	0	0	0
Marinomonas_MWYL1_uid58715	Buchnera_aphidicola_Bp__Baizongia_	66.69	2.22	17	0	0	0	0	0	0	0	0	0	0	0	0	0
Methylococcus_capsulatus_Bath_uid576	Buchnera_aphidicola_Bp__Baizongia_	70.76	0.37	18	0	0	0	0	0	0	0	0	0	0	0	0	0
Nitrosococcus_oceani_ATCC_19707_uid5	Dichelobacter_nodosus_VCS1703A_u	64.76	4.11	19	0	0	0	0	0	0	0	0	0	0	0	0	0
Actinobacillus_pleuropneumoniae_serov	Acinetobacter_baumannii_ATCC_179	66.94	10.04	2	0	0	0	0	0	0	0	0	0	0	0	0	0
Actinobacillus_succinogenes_130Z_uid58	Actinobacillus_pleuropneumoniae_se	71.86	37.05	2	0	1	0	0	0	0	0	0	0	0	0	0	0
Haemophilus_ducreyi_35000HP_uid5762	Actinobacillus_pleuropneumoniae_se	75.31	64.47	2	0	2	0	0	0	0	0	0	0	0	0	0	0
Haemophilus_influenzae_Rd_KW20_uid5	Actinobacillus_pleuropneumoniae_se	72.24	41.84	2	0	3	0	0	0	0	0	0	0	0	0	0	0
Haemophilus_somnus_129PT_uid57929	Haemophilus_influenzae_Rd_KW20_	73.13	43.75	2	0	4	0	0	0	0	0	0	0	0	0	0	0
Mannheimia_succiniciproducens_MBEL5	Actinobacillus_succinogenes_130Z_ui	75.7	54.61	2	0	5	0	0	0	0	0	0	0	0	0	0	0
Pasteurella_multocida_Pm70_uid57627	Haemophilus_influenzae_Rd_KW20_	73.52	43.95	2	0	6	0	0	0	0	0	0	0	0	0	0	0
Photobacterium_profundum_SS9_uid62	Haemophilus_ducreyi_35000HP_uid5	68.93	6.74	20	0	0	0	0	0	0	0	0	0	0	0	0	0
Vibrio_fischeri_ES114_uid58163	Photobacterium_profundum_SS9_uid	71.53	37.27	20	0	1	0	0	0	0	0	0	0	0	0	0	0
Vibrio_cholerae_O1_biovar_El_Tor_N16	Photobacterium_profundum_SS9_uid	69.64	34.25	20	1	0	0	0	0	0	0	0	0	0	0	0	0
Vibrio_parahaemolyticus_RIMD_221063	Vibrio_cholerae_O1_biovar_El_Tor_N	72.65	39.81	20	1	1	0	0	0	0	0	0	0	0	0	0	0
Vibrio_vulnificus_YJ016_uid58007	Vibrio_parahaemolyticus_RIMD_221	74.6	52.35	20	1	2	0	0	0	0	0	0	0	0	0	0	0
Pseudoalteromonas_atlantica_T6c_uid58	Colwellia_psychrerythraea_34H_uid5	66.95	13.8	21	0	0	0	0	0	0	0	0	0	0	0	0	0

Pseudomonas_aeruginosa_PAO1_uid579	Buchnera_aphidicola_Sg__Schizaphis_	72.72	0.49	22	0	0	0	0	0	0	0	0	0	0	0	0	0
Pseudomonas_entomophila_L48_uid586	Pseudomonas_aeruginosa_PAO1_uid	76.74	43.83	22	0	1	0	0	0	0	0	0	0	0	0	0	0
Pseudomonas_putida_KT2440_uid57843	Pseudomonas_entomophila_L48_uid	84.05	59.66	22	0	1	1	0	0	0	0	0	0	0	0	0	0
Pseudomonas_fluorescens_Pf0_1_uid575	Pseudomonas_entomophila_L48_uid	77.46	50.67	22	0	2	0	0	0	0	0	0	0	0	0	0	0
Pseudomonas_fluorescens_Pf_5_uid5793	Pseudomonas_fluorescens_Pf0_1_uic	81	57.29	22	0	2	1	0	0	0	0	0	0	0	0	0	0
Pseudomonas_mendocina_ymp_uid5872	Pseudomonas_aeruginosa_PAO1_uid	77.79	48.75	22	0	3	0	0	0	0	0	0	0	0	0	0	0
Pseudomonas_stutzeri_A1501_uid58641	Pseudomonas_mendocina_ymp_uid5	78.01	47.61	22	0	4	0	0	0	0	0	0	0	0	0	0	0
Pseudomonas_syringae_tomato_DC3000	Pseudomonas_fluorescens_Pf0_1_uic	77.42	45.1	22	0	5	0	0	0	0	0	0	0	0	0	0	0
Psychrobacter_cryohalolentis_K5_uid583	Acinetobacter_baumannii_ATCC_179	67.86	14.14	23	0	0	0	0	0	0	0	0	0	0	0	0	0
Psychrobacter_PRwf_1_uid58459	Psychrobacter_cryohalolentis_K5_uid	72.79	30.02	23	0	1	0	0	0	0	0	0	0	0	0	0	0
Psychromonas_ingrahamii_37_uid58521	Colwellia_psychrerythraea_34H_uid5	67.75	14.45	24	0	0	0	0	0	0	0	0	0	0	0	0	0
Ralstonia_solanacearum_GMI1000_uid57	Buchnera_aphidicola_Sg__Schizaphis_	71.03	0.42	25	0	0	0	0	0	0	0	0	0	0	0	0	0
Rhodospirillum_rubrum_ATCC_11170_ui	Buchnera_aphidicola_APS__Acyrthosi	74.83	0.3	26	0	0	0	0	0	0	0	0	0	0	0	0	0
Rothia_mucilaginosa_uid43093	Buchnera_aphidicola_Bp__Baizongia_	73.35	0.41	27	0	0	0	0	0	0	0	0	0	0	0	0	0
Saccharophagus_degradans_2_40_uid57	Marinomonas_MWYL1_uid58715	65.59	8.65	28	0	0	0	0	0	0	0	0	0	0	0	0	0
Shewanella_amazonensis_SB2B_uid5825	Aeromonas_hydrophila_ATCC_7966_	68.78	19.8	29	0	0	0	0	0	0	0	0	0	0	0	0	0
Shewanella_baltica_OS155_uid58259	Shewanella_amazonensis_SB2B_uid5	70.95	36.24	29	0	1	0	0	0	0	0	0	0	0	0	0	0
Shewanella_putrefaciens_CN_32_uid582	Shewanella_baltica_OS155_uid58259	82.6	67.78	29	0	1	1	0	0	0	0	0	0	0	0	0	0
Shewanella_frigidimarina_NCIMB_400_u	Shewanella_baltica_OS155_uid58259	72.56	40.85	29	0	2	0	0	0	0	0	0	0	0	0	0	0
Shewanella_loihica_PV_4_uid58349	Shewanella_amazonensis_SB2B_uid5	71.46	43.12	29	0	3	0	0	0	0	0	0	0	0	0	0	0
Shewanella_oneidensis_MR_1_uid57949	Shewanella_baltica_OS155_uid58259	79.26	60.21	29	0	4	0	0	0	0	0	0	0	0	0	0	0
Shewanella_pealeana_ATCC_700345_uid	Shewanella_loihica_PV_4_uid58349	71.9	40.15	29	0	5	0	0	0	0	0	0	0	0	0	0	0
Shewanella_woodyi_ATCC_51908_uid58	Shewanella_pealeana_ATCC_700345_	73.43	36.67	29	0	6	0	0	0	0	0	0	0	0	0	0	0
Aeromonas_hydrophila_ATCC_7966_uid	Actinobacillus_pleuropneumoniae_se	66.89	7.55	3	0	0	0	0	0	0	0	0	0	0	0	0	0
Sinorhizobium_meliloti_1021_uid57603	Buchnera_aphidicola_APS__Acyrthosi	72.01	0.18	30	0	0	0	0	0	0	0	0	0	0	0	0	0
Stenotrophomonas_maltophilia_R551_3_	Buchnera_aphidicola_Sg__Schizaphis_	73.78	0.45	31	0	0	0	0	0	0	0	0	0	0	0	0	0
Xanthomonas_axonopodis_citrumelo_F1	Stenotrophomonas_maltophilia_R551	76.14	36.4	31	0	1	0	0	0	0	0	0	0	0	0	0	0
Xanthomonas_campestris_ATCC_33913_	Xanthomonas_axonopodis_citrumelo	84.69	65.02	31	0	1	1	0	0	0	0	0	0	0	0	0	0
Xylella_fastidiosa_9a5c_uid57849	Xanthomonas_axonopodis_citrumelo	70.76	37.87	31	0	2	0	0	0	0	0	0	0	0	0	0	0
Thiomicrospira_crunogena_XCL_2_uid58	Haemophilus_ducreyi_35000HP_uid5	65.34	6.85	32	0	0	0	0	0	0	0	0	0	0	0	0	0
Alcanivorax_borkumensis_SK2_uid58169	Aeromonas_hydrophila_ATCC_7966_	65.9	12.88	4	0	0	0	0	0	0	0	0	0	0	0	0	0
Alkalilimnicola_ehrlichii_MLHE_1_uid584	Aeromonas_hydrophila_ATCC_7966_	67.1	14.07	5	0	0	0	0	0	0	0	0	0	0	0	0	0
Halorhodospira_halophila_SL1_uid58473	Alkalilimnicola_ehrlichii_MLHE_1_uid	70.55	39.64	5	0	1	0	0	0	0	0	0	0	0	0	0	0
Buchnera_aphidicola_APS__Acyrthosipho	Alkalilimnicola_ehrlichii_MLHE_1_uid	64.64	2.33	6	0	0	0	0	0	0	0	0	0	0	0	0	0
Buchnera_aphidicola_Sg__Schizaphis_gra	Buchnera_aphidicola_APS__Acyrthosi	77.02	88.71	6	0	1	0	0	0	0	0	0	0	0	0	0	0
Buchnera_aphidicola_Bp__Baizongia_pist	Buchnera_aphidicola_APS__Acyrthosi	68.42	56.67	6	1	0	0	0	0	0	0	0	0	0	0	0	0
Chromobacterium_violaceum_ATCC_124	Buchnera_aphidicola_Bp__Baizongia_	74.82	0.69	7	0	0	0	0	0	0	0	0	0	0	0	0	0
Chromohalobacter_salexigens_DSM_304	Buchnera_aphidicola_Bp__Baizongia_	73.14	0.77	8	0	0	0	0	0	0	0	0	0	0	0	0	0
Colwellia_psychrerythraea_34H_uid5785	Buchnera_aphidicola_Bp__Baizongia_	68.02	3.08	9	0	0	0	0	0	0	0	0	0	0	0	0	0
Pseudoalteromonas_haloplanktis_TAC12	Colwellia_psychrerythraea_34H_uid5	68.43	21.17	9	1	0	0	0	0	0	0	0	0	0	0	0	0

**Table S2**. *Bacillus anthracis*				code		
**query**	**subject (most similar genome to query genome considering only genomes in rows above the query genome)**	**ANIb %**	**% of aligned fragments**	**V 99.99%**	**W 99.999%**	**X 99.9999%**
A0174	na	na	na	0	0	0
A0193	A0174	99.99679	95.86	0	1	0
Western North America USA61	A0193	99.99571	96.81	0	2	0
Tsiankovskii I	A0193	99.99127	96.89	0	3	0
A0389	A0174	99.98621	95.70	1	0	0
Ames	A0389	99.99343	97.50	1	1	0
Ames_Ancestor	Ames	99.99966	94.94	1	1	1
A0248	Ames_Ancestor	100.00000	99.93	1	1	1
Australia_94	Ames_Ancestor	99.99074	98.95	1	2	0
Sterne	A0389	99.99328	97.66	1	3	0
A0442	A0174	99.96443	96.01	2	0	0
Kruger_B	A0442	99.99637	96.61	2	1	0
A0465	A0442	99.98154	97.41	3	0	0
CNEVA_9066	A0465	99.99547	95.98	3	1	0
A0488	A0174	99.98841	96.05	4	0	0
CDC_684	A0488	99.99485	97.35	4	1	0
Vollum	A0488	99.99895	96.82	4	2	0
A1055	A0174	99.95760	94.40	5	0	0
A2012	A0389	99.92975	92.21	6	0	0
H9401	A0174	99.98821	95.66	7	0	0

**Table S3**. Eukaryotes				Code							
**query**	**subject (most similar genome to query genome considering only genomes in rows above the query genome)**	**ANIb %**	**% of aligned fragments**	A 60%	B 70%	C 80%	D 85%	E 90%	F 95%	G 98%	H 99%
Acanthamoeba castellanii	na	na	na	0	0	0	0	0	0	0	0
Candida parapsilosis	Candida albicans SC5314	75.20	39.39	0	0	1	0	0	0	0	0
Acinonyx jubatus	Acanthamoeba castellanii	67.75	5.88	1	0	0	0	0	0	0	0
Felis catus	Acinonyx jubatus	89.67	94.12	1	0	0	0	1	0	0	0
Arctocephalus forsteri	Acinonyx jubatus	80.23	93.75	1	0	0	1	0	0	0	0
Eumetopias jubatus	Arctocephalus forsteri	92.40	88.24	1	0	0	1	0	1	0	0
Canis lupus familiaris	Acinonyx jubatus	80.69	88.24	1	0	0	2	0	0	0	0
Ceratotherium simum	Acinonyx jubatus	80.41	88.24	1	0	0	3	0	0	0	0
Rhinoceros unicornis	Ceratotherium simum	88.53	94.12	1	0	0	3	1	0	0	0
Equus asinus	Ceratotherium simum	84.24	88.24	1	0	0	4	0	0	0	0
Equus caballus	Equus asinus	93.00	94.12	1	0	0	4	0	1	0	0
Halichoerus grypus	Eumetopias jubatus	84.88	88.24	1	0	0	5	0	0	0	0
Phoca vitulina	Halichoerus grypus	96.15	94.12	1	0	0	5	0	0	1	0
Odobenus rosmarus rosmarus	Eumetopias jubatus	83.97	94.12	1	0	0	6	0	0	0	0
Tapirus terrestris	Ceratotherium simum	83.77	94.12	1	0	0	7	0	0	0	0
Ursus americanus	Halichoerus grypus	82.01	88.24	1	0	0	8	0	0	0	0
Ursus arctos	Ursus americanus	91.37	88.24	1	0	0	8	0	1	0	0
Ursus maritimus	Ursus arctos	97.90	94.12	1	0	0	8	0	1	1	0
Artibeus jamaicensis	Acinonyx jubatus	77.46	88.24	1	0	1	0	0	0	0	0
Echinops telfairi	Chrysochloris asiatica	74.26	94.12	1	0	10	0	0	0	0	0
Echinosorex gymnura	Chrysochloris asiatica	74.14	88.24	1	0	11	0	0	0	0	0
Elephantulus sp VB001	Chrysochloris asiatica	75.91	88.24	1	0	12	0	0	0	0	0
Elephas maximus	Dugong dugon	75.66	94.12	1	0	13	0	0	0	0	0
Loxodonta africana	Elephas maximus	94.91	94.12	1	0	13	0	0	1	0	0
Episoriculus fumidus	Ceratotherium simum	77.95	83.33	1	0	14	0	0	0	0	0
Erinaceus europaeus	Capra hircus	73.01	77.78	1	0	15	0	0	0	0	0
Hemiechinus auritus	Erinaceus europaeus	83.23	88.24	1	0	15	1	0	0	0	0
Galeopterus variegatus	Equus asinus	76.28	88.24	1	0	16	0	0	0	0	0
Glis glis	Equus asinus	74.87	88.24	1	0	17	0	0	0	0	0
Gorilla gorilla	Ceratotherium simum	75.12	88.24	1	0	18	0	0	0	0	0
Homo sapiens	Gorilla gorilla	89.32	88.24	1	0	18	0	1	0	0	0
Pan paniscus	Homo sapiens	91.59	88.24	1	0	18	0	1	1	0	0
Pan troglodytes	Pan paniscus	95.91	94.12	1	0	18	0	1	1	1	0
Pongo abelii	Homo sapiens	85.31	94.12	1	0	18	0	2	0	0	0
Pongo pygmaeus	Pongo abelii	93.65	88.24	1	0	18	0	2	1	0	0
Hylobates lar-mitochondrion	Homo sapiens	84.29	88.24	1	0	18	1	0	0	0	0
Isoodon macrourus	Didelphis virginiana	79.06	88.24	1	0	19	0	0	0	0	0

Macropus robustus	Isoodon macrourus	80.26	88.24	1	0	19	1	0	0	0	0
Trichosurus vulpecula	Macropus robustus	82.77	88.24	1	0	19	2	0	0	0	0
Vombatus ursinus	Trichosurus vulpecula	82.75	88.24	1	0	19	3	0	0	0	0
Balaenoptera acutorostrata	Acinonyx jubatus	78.25	88.24	1	0	2	0	0	0	0	0
Balaenoptera musculus	Balaenoptera acutorostrata	91.48	88.24	1	0	2	0	0	1	0	0
Balaenoptera physalus	Balaenoptera musculus	92.51	94.12	1	0	2	0	0	2	0	0
Eschrichtius robustus	Balaenoptera musculus	92.55	88.24	1	0	2	0	0	3	0	0
Berardius bairdii	Balaenoptera musculus	86.27	88.24	1	0	2	0	1	0	0	0
Caperea marginata	Balaenoptera musculus	89.36	88.24	1	0	2	0	2	0	0	0
Hyperoodon ampullatus	Berardius bairdii	88.84	94.12	1	0	2	0	3	0	0	0
Kogia breviceps	Balaenoptera musculus	85.18	88.24	1	0	2	0	4	0	0	0
Lagenorhynchus albirostris	Balaenoptera musculus	85.26	88.24	1	0	2	0	5	0	0	0
Monodon monoceros	Lagenorhynchus albirostris	88.30	94.12	1	0	2	0	6	0	0	0
Phocoena phocoena	Monodon monoceros	87.83	94.12	1	0	2	0	7	0	0	0
Physeter catodon	Eschrichtius robustus	85.66	88.24	1	0	2	0	8	0	0	0
Platanista minor	Balaenoptera musculus	85.22	88.24	1	0	2	0	9	0	0	0
Bos taurus	Balaenoptera acutorostrata	80.27	82.35	1	0	2	1	0	0	0	0
Capra hircus	Bos taurus	86.37	88.24	1	0	2	1	1	0	0	0
Muntiacus crinifrons	Capra hircus	85.83	88.24	1	0	2	1	2	0	0	0
Muntiacus muntjak	Muntiacus crinifrons	94.99	94.12	1	0	2	1	2	1	0	0
Muntiacus reevesi	Muntiacus crinifrons	94.16	94.12	1	0	2	1	2	2	0	0
Ovis aries	Capra hircus	89.41	94.12	1	0	2	1	3	0	0	0
Hippopotamus amphibius	Balaenoptera musculus	80.92	88.24	1	0	2	2	0	0	0	0
Inia geoffrensis	Balaenoptera musculus	83.86	88.24	1	0	2	3	0	0	0	0
Lama pacos	Hippopotamus amphibius	80.01	94.12	1	0	2	4	0	0	0	0
Pontoporia blainvillei	Inia geoffrensis	83.52	94.12	1	0	2	5	0	0	0	0
Sus scrofa	Ovis aries	81.11	88.24	1	0	2	6	0	0	0	0
Jaculus jaculus	Ceratotherium simum	74.07	88.24	1	0	20	0	0	0	0	0
Lemur catta	Equus caballus	76.96	88.24	1	0	21	0	0	0	0	0
Lepus europaeus	Equus asinus	76.53	83.33	1	0	22	0	0	0	0	0
Oryctolagus cuniculus	Lepus europaeus	85.07	94.12	1	0	22	0	1	0	0	0
Macaca sylvanus	Gorilla gorilla	79.19	88.24	1	0	23	0	0	0	0	0
Papio hamadryas	Macaca sylvanus	85.24	94.12	1	0	23	0	1	0	0	0
Macroscelides proboscideus	Elephantulus sp VB001	78.62	94.12	1	0	24	0	0	0	0	0
Manis tetradactyla	Equus caballus	76.49	88.24	1	0	25	0	0	0	0	0
Microtus kikuchii	Ceratotherium simum	74.27	93.75	1	0	26	0	0	0	0	0
Mogera wogura	Episoriculus fumidus	79.26	88.24	1	0	27	0	0	0	0	0
Talpa europaea	Mogera wogura	85.78	94.12	1	0	27	0	1	0	0	0
Urotrichus talpoides	Talpa europaea	83.06	94.12	1	0	27	1	0	0	0	0
Mus musculus	Microtus kikuchii	78.22	93.75	1	0	28	0	0	0	0	0
Rattus norvegicus	Mus musculus	82.78	93.75	1	0	28	1	0	0	0	0

Nannospalax ehrenbergi	Mus musculus	75.75	88.24	1	0	29	0	0	0	0	0
Cavia porcellus	Capra hircus	73.76	88.24	1	0	3	0	0	0	0	0
Nycticebus coucang	Lemur catta	75.96	88.24	1	0	30	0	0	0	0	0
Ochotona collaris	Lepus europaeus	77.20	94.12	1	0	31	0	0	0	0	0
Ochotona princeps	Ochotona collaris	91.82	94.12	1	0	31	0	0	1	0	0
Ornithorhynchus anatinus	Isoodon macrourus	72.96	82.35	1	0	32	0	0	0	0	0
Tachyglossus aculeatus	Ornithorhynchus anatinus	81.81	94.12	1	0	32	1	0	0	0	0
Orycteropus afer	Chrysochloris asiatica	77.26	88.24	1	0	33	0	0	0	0	0
Procavia capensis	Dugong dugon	76.31	88.24	1	0	34	0	0	0	0	0
Pteropus dasymallus	Equus asinus	79.51	88.24	1	0	35	0	0	0	0	0
Pteropus scapulatus	Pteropus dasymallus	88.92	94.12	1	0	35	0	1	0	0	0
Sciurus vulgaris	Rhinoceros unicornis	76.35	88.24	1	0	36	0	0	0	0	0
Tamandua tetradactyla	Dasypus novemcinctus	77.24	88.24	1	0	37	0	0	0	0	0
Tarsius bancanus	Lemur catta	76.92	88.24	1	0	38	0	0	0	0	0
Thryonomys swinderianus	Sus scrofa	74.95	82.35	1	0	39	0	0	0	0	0
Cebus albifrons	Capra hircus	73.09	88.24	1	0	4	0	0	0	0	0
Tupaia belangeri	Sus scrofa	76.54	88.24	1	0	40	0	0	0	0	0
Chalinolobus tuberculatus	Ceratotherium simum	76.89	88.24	1	0	5	0	0	0	0	0
Chrysochloris asiatica	Acinonyx jubatus	76.25	88.24	1	0	6	0	0	0	0	0
Dasypus novemcinctus	Ceratotherium simum	76.83	88.24	1	0	7	0	0	0	0	0
Didelphis virginiana	Chrysochloris asiatica	73.58	82.35	1	0	8	0	0	0	0	0
Dugong dugon	Ceratotherium simum	76.79	88.24	1	0	9	0	0	0	0	0
Acipenser transmontanus	Acinonyx jubatus	69.36	82.35	1	1	0	0	0	0	0	0
Huso huso	Acipenser transmontanus	94.91	94.12	1	1	0	0	0	1	0	0
Polyodon spathula	Acipenser transmontanus	90.06	88.24	1	1	0	0	0	2	0	0
Scaphirhynchus cf albus	Huso huso	93.38	88.24	1	1	0	0	0	3	0	0
Alepocephalus tenebrosus	Acipenser transmontanus	77.43	88.24	1	1	1	0	0	0	0	0
Platytroctes apus	Alepocephalus tenebrosus	88.76	94.12	1	1	1	0	1	0	0	0
Chanos chanos	Alepocephalus tenebrosus	80.08	93.75	1	1	1	1	0	0	0	0
Arcos sp KU-149	Anoplogaster cornuta	75.15	88.24	1	1	10	0	0	0	0	0
Arctoscopus japonicus	Antigonia capros	78.67	88.24	1	1	11	0	0	0	0	0
Cottus reinii	Arctoscopus japonicus	82.35	94.12	1	1	11	1	0	0	0	0
Gasterosteus aculeatus	Cottus reinii	80.08	93.75	1	1	11	2	0	0	0	0
Hypoptychus dybowskii	Gasterosteus aculeatus	82.39	88.24	1	1	11	3	0	0	0	0
Lycodes toyamensis	Cottus reinii	81.92	94.12	1	1	11	4	0	0	0	0
Pholis crassispina	Lycodes toyamensis	86.24	94.12	1	1	11	4	1	0	0	0
Ateleopus japonicus	Anoplogaster cornuta	77.89	88.24	1	1	12	0	0	0	0	0
Ijimaia dofleini	Ateleopus japonicus	89.50	94.12	1	1	12	0	1	0	0	0
Aulopus japonicus	Anoplogaster cornuta	78.87	88.24	1	1	13	0	0	0	0	0
Bassozetus zenkevitchi	Auxis rochei	78.14	88.24	1	1	14	0	0	0	0	0
Lamprogrammus niger	Bassozetus zenkevitchi	80.78	94.12	1	1	14	1	0	0	0	0

Carapus bermudensis	Bassozetus zenkevitchi	72.10	82.35	1	1	15	0	0	0	0	0
Carassius auratus	Alepocephalus tenebrosus	78.15	88.24	1	1	16	0	0	0	0	0
Cyprinus carpio	Carassius auratus	89.43	94.12	1	1	16	0	1	0	0	0
Carpiodes carpio	Carassius auratus	81.63	88.24	1	1	16	1	0	0	0	0
Cobitis striata	Carpiodes carpio	80.90	94.12	1	1	16	2	0	0	0	0
Formosania lacustris	Cobitis striata	80.06	94.12	1	1	16	3	0	0	0	0
Lefua echigonia	Cobitis striata	81.52	94.12	1	1	16	4	0	0	0	0
Sarcocheilichthys variegatus micro	Cyprinus carpio	82.84	88.24	1	1	16	5	0	0	0	0
Caulophryne jordani	Auxis rochei	76.27	82.35	1	1	17	0	0	0	0	0
Cetostoma regani	Beryx splendens	78.70	94.12	1	1	18	0	0	0	0	0
Eutaeniophorus sp 033-Miya	Cetostoma regani	99.96	94.12	1	1	18	0	0	0	0	0
Chalceus macrolepidotus	Carassius auratus	79.19	88.24	1	1	19	0	0	0	0	0
Eigenmannia sp CBM-ZF-10620	Chalceus macrolepidotus	80.02	88.24	1	1	19	1	0	0	0	0
Allocyttus niger	Alepocephalus tenebrosus	75.71	88.24	1	1	2	0	0	0	0	0
Neocyttus rhomboidalis	Allocyttus niger	96.78	94.12	1	1	2	0	0	0	1	0
Zenion japonicum	Neocyttus rhomboidalis	85.25	94.12	1	1	2	0	1	0	0	0
Parazen pacificus	Neocyttus rhomboidalis	82.66	94.12	1	1	2	1	0	0	0	0
Zenopsis nebulosus	Neocyttus rhomboidalis	83.75	93.75	1	1	2	2	0	0	0	0
Zeus faber	Zenopsis nebulosus	84.22	94.12	1	1	2	3	0	0	0	0
Chauliodus sloani	Aulopus japonicus	73.18	72.22	1	1	20	0	0	0	0	0
Chaunax abei	Antigonia capros	77.94	94.12	1	1	21	0	0	0	0	0
Chaunax tosaensis	Chaunax abei	97.35	94.12	1	1	21	0	0	0	1	0
Melanocetus murrayi	Chaunax tosaensis	80.32	88.24	1	1	21	1	0	0	0	0
Chimaera monstrosa	Amblyraja radiata	72.12	78.95	1	1	22	0	0	0	0	0
Chlorophthalmus agassizi	Aulopus japonicus	77.72	87.50	1	1	23	0	0	0	0	0
Coelorinchus kishinouyei	Allocyttus niger	73.85	87.50	1	1	24	0	0	0	0	0
Cololabis saira	Auxis thazard	77.35	88.24	1	1	25	0	0	0	0	0
Conger myriaster	Anguilla japonica	79.08	73.68	1	1	26	0	0	0	0	0
Coregonus lavaretus	Alepocephalus tenebrosus	79.21	88.24	1	1	27	0	0	0	0	0
Oncorhynchus mykiss	Coregonus lavaretus	86.11	94.12	1	1	27	0	1	0	0	0
Oncorhynchus tshawytscha	Oncorhynchus mykiss	93.32	94.12	1	1	27	0	1	1	0	0
Salmo salar	Oncorhynchus tshawytscha	89.93	94.12	1	1	27	0	2	0	0	0
Salvelinus alpinus	Salmo salar	90.45	94.12	1	1	27	0	2	1	0	0
Salvelinus fontinalis	Salvelinus alpinus	95.37	94.12	1	1	27	0	2	1	1	0
Corydoras rabauti	Chalceus macrolepidotus	79.81	88.24	1	1	28	0	0	0	0	0
Crenimugil crenilabis	Auxis thazard	77.29	93.75	1	1	29	0	0	0	0	0
Mugil cephalus	Crenimugil crenilabis	95.64	94.12	1	1	29	0	0	0	1	0
Amblyraja radiata	Acipenser transmontanus	73.20	88.24	1	1	3	0	0	0	0	0
Dactyloptena peterseni	Auxis thazard	77.84	88.24	1	1	30	0	0	0	0	0
Dactyloptena tiltoni	Dactyloptena peterseni	87.71	94.12	1	1	30	0	1	0	0	0
Dallia pectoralis	Coregonus lavaretus	77.56	88.24	1	1	31	0	0	0	0	0

Esox lucius	Dallia pectoralis	80.42	94.12	1	1	31	1	0	0	0	0
Danacetichthys galathenus	Cetostoma regani	77.92	88.24	1	1	32	0	0	0	0	0
Danio rerio	Carassius auratus	78.17	94.12	1	1	33	0	0	0	0	0
Diaphus splendidus	Beryx splendens	77.45	87.50	1	1	34	0	0	0	0	0
Myctophum affine	Diaphus splendidus	80.09	93.75	1	1	34	1	0	0	0	0
Neoscopelus microchir	Diaphus splendidus	80.20	82.35	1	1	34	2	0	0	0	0
Diplophos taenia	Coregonus lavaretus	76.28	88.24	1	1	35	0	0	0	0	0
Elassoma evergladei	Antigonia capros	76.26	93.75	1	1	36	0	0	0	0	0
Eleotris acanthopoma	Auxis rochei	79.22	88.24	1	1	37	0	0	0	0	0
Rhyacichthys aspro	Eleotris acanthopoma	81.27	94.12	1	1	37	1	0	0	0	0
Engraulis japonicus	Alepocephalus tenebrosus	75.06	88.24	1	1	38	0	0	0	0	0
Erpetoichthys calabaricus	Carassius auratus	72.49	76.47	1	1	39	0	0	0	0	0
Polypterus ornatipinnis	Erpetoichthys calabaricus	80.86	94.12	1	1	39	1	0	0	0	0
Polypterus senegalus senegalus	Polypterus ornatipinnis	85.69	94.12	1	1	39	1	1	0	0	0
Amia calva	Acipenser transmontanus	78.34	93.75	1	1	4	0	0	0	0	0
Eurypharynx pelecanoides	Anguilla japonica	77.56	68.42	1	1	40	0	0	0	0	0
Exocoetus volitans	Cololabis saira	78.06	94.12	1	1	41	0	0	0	0	0
Fejervarya limnocharis	Coelorinchus kishinouyei	70.48	50.00	1	1	42	0	0	0	0	0
Gadus chalcogrammus	Anoplogaster cornuta	76.60	88.24	1	1	43	0	0	0	0	0
Gadus morhua	Gadus chalcogrammus	95.89	88.24	1	1	43	0	0	0	1	0
Lota lota	Gadus chalcogrammus	86.67	88.24	1	1	43	0	1	0	0	0
Galaxias maculatus	Coregonus lavaretus	76.35	88.24	1	1	44	0	0	0	0	0
Gambusia affinis	Auxis rochei	75.02	82.35	1	1	45	0	0	0	0	0
Glossanodon semifasciatus	Coregonus lavaretus	79.07	88.24	1	1	46	0	0	0	0	0
Lipolagus ochotensis	Glossanodon semifasciatus	81.16	88.24	1	1	46	1	0	0	0	0
Nansenia ardesiaca	Lipolagus ochotensis	84.88	94.12	1	1	46	2	0	0	0	0
Opisthoproctus soleatus	Nansenia ardesiaca	81.61	94.12	1	1	46	3	0	0	0	0
Gonorynchus greyi	Alepocephalus tenebrosus	75.92	88.24	1	1	47	0	0	0	0	0
Gymnothorax kidako	Anguilla japonica	76.76	88.24	1	1	48	0	0	0	0	0
Harpadon microchir	Aulopus japonicus	78.34	93.75	1	1	49	0	0	0	0	0
Saurida undosquamis	Harpadon microchir	83.13	93.75	1	1	49	1	0	0	0	0
Anguilla japonica	Acipenser transmontanus	77.15	88.24	1	1	5	0	0	0	0	0
Heterodontus francisci	Amblyraja radiata	78.28	88.24	1	1	50	0	0	0	0	0
Mustelus manazo	Heterodontus francisci	81.78	94.12	1	1	50	1	0	0	0	0
Scyliorhinus canicula	Mustelus manazo	83.03	88.24	1	1	50	2	0	0	0	0
Squalus acanthias	Heterodontus francisci	82.46	94.12	1	1	50	3	0	0	0	0
Hiodon alosoides	Anguilla japonica	78.54	88.24	1	1	51	0	0	0	0	0
Hypoatherina tsurugae	Emmelichthys struhsakeri	79.98	88.24	1	1	52	0	0	0	0	0
Ictalurus punctatus	Corydoras rabauti	79.60	88.24	1	1	53	0	0	0	0	0
Pseudobagrus tokiensis	Ictalurus punctatus	83.46	88.24	1	1	53	1	0	0	0	0
Indostomus paradoxus	Diplacanthopoma brachysoma	75.54	93.75	1	1	54	0	0	0	0	0

Kareius bicoloratus	Carangoides armatus	79.43	93.75	1	1	55	0	0	0	0	0
Paralichthys olivaceus	Kareius bicoloratus	82.57	88.24	1	1	55	1	0	0	0	0
Kryptolebias marmoratus	Antigonia capros	73.04	88.24	1	1	56	0	0	0	0	0
Lampris guttatus	Beryx splendens	75.46	93.75	1	1	57	0	0	0	0	0
Latimeria chalumnae	Huso huso	74.98	88.24	1	1	58	0	0	0	0	0
Lepidosiren paradoxa	Corydoras rabauti	70.41	82.35	1	1	59	0	0	0	0	0
Anoplogaster cornuta	Alepocephalus tenebrosus	77.85	88.24	1	1	6	0	0	0	0	0
Hoplostethus japonicus	Anoplogaster cornuta	87.46	94.12	1	1	6	0	1	0	0	0
Monocentris japonicus	Hoplostethus japonicus	86.97	94.12	1	1	6	0	2	0	0	0
Beryx decadactylus	Anoplogaster cornuta	81.45	94.12	1	1	6	1	0	0	0	0
Beryx splendens	Beryx decadactylus	92.98	94.12	1	1	6	1	0	1	0	0
Poromitra oscitans	Beryx splendens	85.90	88.24	1	1	6	1	1	0	0	0
Cataetyx rubrirostris	Anoplogaster cornuta	80.24	88.24	1	1	6	2	0	0	0	0
Diplacanthopoma brachysoma	Cataetyx rubrirostris	86.56	94.12	1	1	6	2	1	0	0	0
Myripristis berndti	Beryx splendens	81.69	88.24	1	1	6	3	0	0	0	0
Ostichthys japonicus	Myripristis berndti	86.32	94.12	1	1	6	3	1	0	0	0
Polymixia japonica	Hoplostethus japonicus	81.13	88.24	1	1	6	4	0	0	0	0
Polymixia lowei	Polymixia japonica	93.15	94.12	1	1	6	4	0	1	0	0
Rondeletia loricata	Beryx splendens	83.46	94.12	1	1	6	5	0	0	0	0
Sargocentron rubrum	Ostichthys japonicus	84.78	88.24	1	1	6	6	0	0	0	0
Scopelogadus mizolepis	Poromitra oscitans	84.40	76.47	1	1	6	7	0	0	0	0
Lepisosteus oculatus	Huso huso	78.27	82.35	1	1	60	0	0	0	0	0
Lophius americanus	Emmelichthys struhsakeri	78.75	94.12	1	1	61	0	0	0	0	0
Mastacembelus favus	Katsuwonus pelamis	79.49	88.24	1	1	62	0	0	0	0	0
Melanotaenia lacustris	Hypoatherina tsurugae	78.97	94.12	1	1	63	0	0	0	0	0
Monopterus albus	Katsuwonus pelamis	74.06	88.24	1	1	64	0	0	0	0	0
Neoceratodus forsteri	Huso huso	75.79	88.24	1	1	65	0	0	0	0	0
Notacanthus chemnitzi	Anguilla japonica	77.67	93.75	1	1	66	0	0	0	0	0
Oryzias latipes	Emmelichthys struhsakeri	75.91	88.24	1	1	67	0	0	0	0	0
Osteoglossum bicirrhosum	Hiodon alosoides	77.12	93.75	1	1	68	0	0	0	0	0
Pantodon buchholzi	Hiodon alosoides	77.07	93.75	1	1	69	0	0	0	0	0
Antigonia capros	Anoplogaster cornuta	79.60	88.24	1	1	7	0	0	0	0	0
Auxis rochei	Antigonia capros	80.07	94.12	1	1	7	1	0	0	0	0
Auxis thazard	Auxis rochei	95.33	94.12	1	1	7	1	0	0	1	0
Euthynnus alletteratus	Auxis rochei	90.92	94.12	1	1	7	1	0	1	0	0
Katsuwonus pelamis	Auxis rochei	92.12	94.12	1	1	7	1	0	2	0	0
Thunnus alalunga	Katsuwonus pelamis	89.77	94.12	1	1	7	1	1	0	0	0
Thunnus thynnus thynnus	Thunnus alalunga	97.25	88.24	1	1	7	1	1	0	1	0
Carangoides armatus	Auxis rochei	80.65	88.24	1	1	7	2	0	0	0	0
Caranx melampygus	Carangoides armatus	86.43	94.12	1	1	7	2	1	0	0	0
Trachurus japonicus	Caranx melampygus	86.92	88.24	1	1	7	2	2	0	0	0

Emmelichthys struhsakeri	Auxis rochei	83.10	88.24	1	1	7	3	0	0	0	0
Pterocaesio tile	Emmelichthys struhsakeri	85.37	94.12	1	1	7	3	1	0	0	0
Etheostoma radiosum	Emmelichthys struhsakeri	81.06	88.24	1	1	7	4	0	0	0	0
Helicolenus hilgendorfi	Emmelichthys struhsakeri	81.42	94.12	1	1	7	5	0	0	0	0
Pagrus auriga	Emmelichthys struhsakeri	80.64	88.24	1	1	7	6	0	0	0	0
Pagrus major	Pagrus auriga	89.12	88.24	1	1	7	6	1	0	0	0
Scalicus amiscus	Emmelichthys struhsakeri	81.98	94.12	1	1	7	7	0	0	0	0
Percopsis transmontana	Aphredoderus sayanus	77.95	93.75	1	1	70	0	0	0	0	0
Petroscirtes breviceps	Gadus morhua	73.24	76.47	1	1	71	0	0	0	0	0
Phenacogrammus interruptus	Chalceus macrolepidotus	78.74	94.12	1	1	72	0	0	0	0	0
Physiculus japonicus	Lota lota	77.78	70.59	1	1	73	0	0	0	0	0
Plecoglossus altivelis	Coregonus lavaretus	77.81	88.24	1	1	74	0	0	0	0	0
Salangichthys microdon	Plecoglossus altivelis	83.21	88.24	1	1	74	1	0	0	0	0
Protopterus dolloi	Lepidosiren paradoxa	71.77	88.24	1	1	75	0	0	0	0	0
Rana nigromaculata	Fejervarya limnocharis	72.62	66.67	1	1	76	0	0	0	0	0
Retropinna retropinna	Plecoglossus altivelis	79.17	94.12	1	1	77	0	0	0	0	0
Saccopharynx lavenbergi	Anguilla japonica	73.78	68.42	1	1	78	0	0	0	0	0
Salarias fasciatus	Katsuwonus pelamis	76.34	88.24	1	1	79	0	0	0	0	0
Aphredoderus sayanus	Anoplogaster cornuta	77.01	88.24	1	1	8	0	0	0	0	0
Sardinops melanostictus	Engraulis japonicus	76.91	88.24	1	1	80	0	0	0	0	0
Sigmops gracilis	Salmo salar	74.67	82.35	1	1	81	0	0	0	0	0
Stephanolepis cirrhifer	Pterocaesio tile	76.67	93.75	1	1	82	0	0	0	0	0
Sufflamen fraenatum	Pterocaesio tile	78.90	94.12	1	1	83	0	0	0	0	0
Takifugu rubripes	Pterocaesio tile	77.58	94.12	1	1	84	0	0	0	0	0
Trachipterus trachypterus	Ostichthys japonicus	74.46	93.75	1	1	85	0	0	0	0	0
Zu cristatus	Trachipterus trachypterus	80.24	93.75	1	1	85	1	0	0	0	0
Apteronotus albifrons	Alepocephalus tenebrosus	76.25	87.50	1	1	9	0	0	0	0	0
Daphnia pulex	Artemia franciscana	67.36	25.00	1	10	0	0	0	0	0	0
Dinodon semicarinatus	Alligator mississippiensis	67.84	23.53	1	11	0	0	0	0	0	0
Eptatretus burgeri	Corydoras rabauti	67.57	41.18	1	12	0	0	0	0	0	0
Myxine glutinosa	Eptatretus burgeri	77.79	73.68	1	12	1	0	0	0	0	0
Florometra serratissima	Drosophila yakuba	67.77	25.00	1	13	0	0	0	0	0	0
Katharina tunicata	Ceratitis capitata	68.83	25.00	1	14	0	0	0	0	0	0
Lampetra fluviatilis	Aphredoderus sayanus	68.91	62.50	1	15	0	0	0	0	0	0
Petromyzon marinus	Lampetra fluviatilis	87.85	93.75	1	15	0	0	1	0	0	0
Lithobius forficatus	Antheraea pernyi	67.23	31.25	1	16	0	0	0	0	0	0
Loligo bleekeri	Locusta migratoria	67.08	23.53	1	17	0	0	0	0	0	0
Paguru longicarpus	Anopheles gambiae	69.36	50.00	1	18	0	0	0	0	0	0
Penaeus monodon	Paguru longicarpus	70.83	56.25	1	18	1	0	0	0	0	0
Portunus trituberculatus	Paguru longicarpus	72.80	50.00	1	18	2	0	0	0	0	0
Panulirus japonicus	Paguru longicarpus	68.51	62.50	1	19	0	0	0	0	0	0

Alligator mississippiensis	Alepocephalus tenebrosus	68.74	52.94	1	2	0	0	0	0	0	0
Alligator sinensis	Alligator mississippiensis	81.63	94.12	1	2	0	1	0	0	0	0
Caiman crocodilus	Alligator mississippiensis	77.89	83.33	1	2	1	0	0	0	0	0
Patiria pectinifera	Paracentrotus lividus	66.53	56.25	1	20	0	0	0	0	0	0
Platynereis dumerilii	Anopheles quadrimaculatus A-mitochondrion.fna	66.30	25.00	1	21	0	0	0	0	0	0
Tetrodontophora bielanensis	Drosophila yakuba	68.97	56.25	1	22	0	0	0	0	0	0
Thrips imaginis	Drosophila melanogaster	68.14	25.00	1	23	0	0	0	0	0	0
Triatoma dimidiata	Chrysomya putoria	69.07	70.59	1	24	0	0	0	0	0	0
Triops cancriformis	Drosophila melanogaster	68.99	80.00	1	25	0	0	0	0	0	0
Typhlonectes natans	Katsuwonus pelamis	69.58	70.59	1	26	0	0	0	0	0	0
Varroa destructor	Bombyx mandarina	67.04	29.41	1	27	0	0	0	0	0	0
Andrias davidianus	Amia calva	68.77	88.24	1	3	0	0	0	0	0	0
Lyciasalamandra atifi	Andrias davidianus	72.61	88.24	1	3	1	0	0	0	0	0
Ranodon sibiricus	Andrias davidianus	76.25	88.24	1	3	2	0	0	0	0	0
Xenopus laevis	Ranodon sibiricus	72.11	77.78	1	3	3	0	0	0	0	0
Anomalopteryx didiformis	Anguilla japonica	69.87	70.59	1	4	0	0	0	0	0	0
Dinornis giganteus	Anomalopteryx didiformis	95.04	82.35	1	4	0	0	0	0	1	0
Emeus crassus	Anomalopteryx didiformis	97.46	82.35	1	4	0	0	0	0	2	0
Apteryx haastii	Anomalopteryx didiformis	84.12	82.35	1	4	0	1	0	0	0	0
Pterocnemia pennata	Dromaius novaehollandiae	83.69	82.35	1	4	0	10	0	0	0	0
Rhea americana	Pterocnemia pennata	93.54	94.12	1	4	0	10	0	1	0	0
Struthio camelus	Dromaius novaehollandiae	83.05	94.12	1	4	0	11	0	0	0	0
Tinamus major	Anomalopteryx didiformis	80.11	82.35	1	4	0	12	0	0	0	0
Arenaria interpres	Apteryx haastii	80.65	88.24	1	4	0	2	0	0	0	0
Buteo buteo	Arenaria interpres	82.42	73.68	1	4	0	3	0	0	0	0
Casuarius casuarius	Anomalopteryx didiformis	84.59	82.35	1	4	0	4	0	0	0	0
Dromaius novaehollandiae	Casuarius casuarius	88.98	94.12	1	4	0	4	1	0	0	0
Ciconia boyciana	Arenaria interpres	84.29	83.33	1	4	0	5	0	0	0	0
Ciconia ciconia	Ciconia boyciana	96.28	94.44	1	4	0	5	0	0	1	0
Eudyptula minor	Ciconia boyciana	85.27	83.33	1	4	0	5	1	0	0	0
Coturnix chinensis	Ciconia boyciana	80.07	88.24	1	4	0	6	0	0	0	0
Coturnix japonica	Coturnix chinensis	88.98	94.12	1	4	0	6	1	0	0	0
Gallus gallus	Coturnix japonica	85.92	88.24	1	4	0	6	2	0	0	0
Eudromia elegans	Anomalopteryx didiformis	80.02	77.78	1	4	0	7	0	0	0	0
Falco peregrinus	Ciconia ciconia	82.00	77.78	1	4	0	8	0	0	0	0
Haematopus ater	Ciconia boyciana	84.76	88.24	1	4	0	9	0	0	0	0
Anser albifrons	Anomalopteryx didiformis	79.39	88.24	1	4	1	0	0	0	0	0
Aythya americana	Anser albifrons	87.10	94.12	1	4	1	0	1	0	0	0
Chelonia mydas	Anomalopteryx didiformis	73.49	82.35	1	4	2	0	0	0	0	0
Chrysemys picta	Chelonia mydas	84.34	82.35	1	4	2	1	0	0	0	0
Corvus frugilegus	Ciconia boyciana	79.86	88.24	1	4	3	0	0	0	0	0

Vidua chalybeata	Corvus frugilegus	82.82	94.12	1	4	3	1	0	0	0	0
Dogania subplana	Chelonia mydas	79.19	88.24	1	4	4	0	0	0	0	0
Iguana iguana	Chelonia mydas	72.32	88.24	1	4	5	0	0	0	0	0
Pelomedusa subrufa	Chrysemys picta	73.20	76.47	1	4	6	0	0	0	0	0
Plestiodon egregius	Iguana iguana	73.50	83.33	1	4	7	0	0	0	0	0
Smithornis sharpei	Ciconia boyciana	79.75	77.78	1	4	8	0	0	0	0	0
Sphenodon punctatus	Chrysemys picta	70.18	60.00	1	4	9	0	0	0	0	0
Anopheles gambiae	Acinonyx jubatus	64.00	25.00	1	5	0	0	0	0	0	0
Anopheles quadrimaculatus A-mito	Anopheles gambiae	89.68	93.75	1	5	0	0	1	0	0	0
Antheraea pernyi	Anopheles gambiae	73.54	93.75	1	5	1	0	0	0	0	0
Bombyx mandarina	Antheraea pernyi	83.22	81.25	1	5	1	1	0	0	0	0
Bombyx mori	Bombyx mandarina	95.74	93.75	1	5	1	1	0	0	1	0
Ostrinia furnacalis	Bombyx mandarina	83.26	86.67	1	5	1	2	0	0	0	0
Ostrinia nubilalis	Ostrinia furnacalis	98.54	93.33	1	5	1	2	0	0	0	1
Artemia franciscana	Anopheles quadrimaculatus A-mitochondrion.fna	70.11	25.00	1	5	2	0	0	0	0	0
Bactrocera oleae	Anopheles gambiae	76.66	87.50	1	5	3	0	0	0	0	0
Ceratitis capitata	Bactrocera oleae	84.76	93.75	1	5	3	1	0	0	0	0
Chrysomya putoria	Ceratitis capitata	82.49	93.75	1	5	3	2	0	0	0	0
Cochliomyia hominivorax	Chrysomya putoria	91.28	87.50	1	5	3	2	0	1	0	0
Drosophila melanogaster	Chrysomya putoria	83.98	70.00	1	5	3	3	0	0	0	0
Drosophila yakuba	Drosophila melanogaster	92.52	93.75	1	5	3	3	0	1	0	0
Crioceris duodecimpunctata	Ceratitis capitata	71.87	87.50	1	5	4	0	0	0	0	0
Lepidopsocid sp-RS-2001	Ceratitis capitata	71.95	64.71	1	5	5	0	0	0	0	0
Locusta migratoria	Chrysomya putoria	72.83	81.25	1	5	6	0	0	0	0	0
Pyrocoelia rufa	Bombyx mandarina	72.06	61.11	1	5	7	0	0	0	0	0
Tribolium castaneum	Crioceris duodecimpunctata	70.80	81.25	1	5	8	0	0	0	0	0
Apis mellifera ligustica	Antheraea pernyi	67.66	58.82	1	6	0	0	0	0	0	0
Melipona bicolor	Apis mellifera ligustica	76.50	60.00	1	6	1	0	0	0	0	0
Arbacia lixula	Andrias davidianus	67.80	25.00	1	7	0	0	0	0	0	0
Paracentrotus lividus	Arbacia lixula	72.84	93.75	1	7	1	0	0	0	0	0
Strongylocentrotus purpuratus	Paracentrotus lividus	74.83	93.75	1	7	2	0	0	0	0	0
Balanoglossus carnosus	Amia calva	66.23	37.50	1	8	0	0	0	0	0	0
Carios capensis	Anopheles gambiae	66.21	40.00	1	9	0	0	0	0	0	0
Ornithodoros moubata	Carios capensis	70.97	93.33	1	9	1	0	0	0	0	0
Branchiostoma belcheri	Antheraea pernyi	69.30	20.00	10	0	0	0	0	0	0	0
Branchiostoma lanceolatum	Branchiostoma belcheri	75.33	93.33	10	0	1	0	0	0	0	0
Cafeteria roenbergensis	Antheraea pernyi	68.78	4.65	11	0	0	0	0	0	0	0
Candida glabrata	Bombyx mandarina	60.91	5.00	12	0	0	0	0	0	0	0
Pichia canadensis	Candida glabrata	71.52	21.43	12	0	1	0	0	0	0	0
Saccharomyces castellii	Candida glabrata	76.72	26.92	12	0	2	0	0	0	0	0
Saccharomyces servazzii	Saccharomyces castellii	76.38	22.58	12	0	3	0	0	0	0	0

Monosiga brevicollis	Candida glabrata	61.33	22.37	12	1	0	0	0	0	0	0
Cepaea nemoralis	Albinaria caerulea	70.41	14.29	13	0	0	0	0	0	0	0
Chaetosphaeridium globosum	Beta vulgaris subsp vulgaris	77.29	7.14	14	0	0	0	0	0	0	0
Malawimonas jakobiformis	Chaetosphaeridium globosum	70.40	21.28	14	0	1	0	0	0	0	0
Phytophthora infestans	Malawimonas jakobiformis	72.46	21.05	14	0	2	0	0	0	0	0
Chondrus crispus	Chaetosphaeridium globosum	65.63	30.77	14	1	0	0	0	0	0	0
Porphyra purpurea	Chondrus crispus	70.28	27.03	14	1	1	0	0	0	0	0
Chara vulgaris	Beta vulgaris subsp vulgaris	76.33	5.97	15	0	0	0	0	0	0	0
Chlamydomonas eugametos	Acropora tenuis	66.01	4.35	16	0	0	0	0	0	0	0
Chlamydomonas reinhardtii	Chlamydomonas eugametos	73.21	6.25	17	0	0	0	0	0	0	0
Chrysodidymus synuroideus	Cafeteria roenbergensis	70.68	11.76	18	0	0	0	0	0	0	0
Ochromonas danica	Chrysodidymus synuroideus	71.78	36.59	18	0	1	0	0	0	0	0
Cyanidioschyzon merolae	Chrysodidymus synuroideus	67.54	21.88	18	1	0	0	0	0	0	0
Laminaria digitata	Chrysodidymus synuroideus	68.81	21.05	18	2	0	0	0	0	0	0
Pylaiella littoralis	Laminaria digitata	77.53	39.66	18	2	1	0	0	0	0	0
Ciona intestinalis	Antheraea pernyi	64.90	20.00	19	0	0	0	0	0	0	0
Ciona savignyi	Ciona intestinalis	76.43	66.67	19	0	1	0	0	0	0	0
Acropora tenuis	Acinonyx jubatus	69.03	5.56	2	0	0	0	0	0	0	0
Metridium senile	Acropora tenuis	74.84	50.00	2	0	1	0	0	0	0	0
Crassostrea gigas	Andrias davidianus	63.36	5.56	20	0	0	0	0	0	0	0
Cryptococcus neoformans var grub	Chrysomya putoria	66.07	4.00	21	0	0	0	0	0	0	0
Dictyostelium discoideum	Chara vulgaris	68.36	5.45	22	0	0	0	0	0	0	0
Echinococcus multilocularis	Chondrus crispus	63.70	7.14	23	0	0	0	0	0	0	0
Hymenolepis diminuta	Echinococcus multilocularis	71.75	71.43	23	0	1	0	0	0	0	0
Taenia asiatica	Echinococcus multilocularis	76.26	85.71	23	0	2	0	0	0	0	0
Taenia solium	Taenia asiatica	84.53	92.86	23	0	2	1	0	0	0	0
Taenia crassiceps	Taenia asiatica	77.58	92.86	23	0	3	0	0	0	0	0
Fasciola hepatica	Echinococcus multilocularis	64.67	46.67	23	1	0	0	0	0	0	0
Paragonimus westermani	Fasciola hepatica	66.12	60.00	23	2	0	0	0	0	0	0
Schistosoma japonicum	Fasciola hepatica	65.95	21.43	23	3	0	0	0	0	0	0
Schistosoma mekongi	Schistosoma japonicum	77.43	92.86	23	3	1	0	0	0	0	0
Schistosoma mansoni	Schistosoma japonicum	67.74	46.67	23	4	0	0	0	0	0	0
Emiliania huxleyi	Artemia franciscana	69.09	3.45	24	0	0	0	0	0	0	0
Haemaphysalis flava	Didelphis virginiana	67.64	20.00	25	0	0	0	0	0	0	0
Rhipicephalus sanguineus	Haemaphysalis flava	73.35	93.33	25	0	1	0	0	0	0	0
Ixodes hexagonus	Haemaphysalis flava	67.96	80.00	25	1	0	0	0	0	0	0
Ixodes holocyclus	Ixodes hexagonus	72.29	86.67	25	1	1	0	0	0	0	0
Ixodes persulcatus	Ixodes hexagonus	77.36	93.33	25	1	2	0	0	0	0	0
Halocynthia roretzi	Ciona intestinalis	65.16	20.00	26	0	0	0	0	0	0	0
Harpochytrium sp-JEL105	Chrysomya putoria	68.99	4.17	27	0	0	0	0	0	0	0
Harpochytrium sp-JEL94	Harpochytrium sp-JEL105	91.43	40.00	27	0	0	0	0	1	0	0

Heterodoxus macropus	Ceratitis capitata	74.68	6.67	28	0	0	0	0	0	0	0
Hyaloraphidium curvatum	Harpochytrium sp-JEL94	70.90	16.67	29	0	0	0	0	0	0	0
Albinaria caerulea	Acropora tenuis	70.65	7.14	3	0	0	0	0	0	0	0
Pupa strigosa	Albinaria caerulea	66.17	21.43	3	1	0	0	0	0	0	0
Hypocrea jecorina	Candida parapsilosis	65.44	9.52	30	0	0	0	0	0	0	0
Lecanicillium muscarium	Hypocrea jecorina	80.52	40.00	30	0	0	1	0	0	0	0
Penicillium marneffei	Hypocrea jecorina	74.52	31.43	30	0	1	0	0	0	0	0
Lampsilis ornata	Florometra serratissima	75.37	6.25	31	0	0	0	0	0	0	0
Laqueus rubellus	Florometra serratissima	71.55	7.14	32	0	0	0	0	0	0	0
Terebratalia transversa	Laqueus rubellus	67.11	40.00	32	1	0	0	0	0	0	0
Leishmania tarentolae kinetoplast.	Apis mellifera ligustica	63.80	4.76	33	0	0	0	0	0	0	0
Limulus polyphemus	Haemaphysalis flava	69.04	20.00	34	0	0	0	0	0	0	0
Lumbricus terrestris	Drosophila melanogaster	66.26	20.00	35	0	0	0	0	0	0	0
Marchantia polymorpha	Beta vulgaris subsp vulgaris	85.19	1.09	36	0	0	0	0	0	0	0
Monoblepharella sp JEL15-mitocho	Harpochytrium sp-JEL105	88.25	6.67	37	0	0	0	0	0	0	0
Naegleria gruberi	Chaetosphaeridium globosum	68.97	6.12	38	0	0	0	0	0	0	0
Narceus annularus	Chelonia mydas	70.21	20.00	39	0	0	0	0	0	0	0
Allomyces macrogynus	Acropora tenuis	60.65	1.75	4	0	0	0	0	0	0	0
Ophiopholis aculeata	Chlorophthalmus agassizi	72.48	5.88	40	0	0	0	0	0	0	0
Paramecium aurelia	Chrysodidymus synuroideus	67.95	2.50	41	0	0	0	0	0	0	0
Pedinomonas minor	Cyanidioschyzon merolae	71.33	4.00	42	0	0	0	0	0	0	0
Physarum polycephalum	Drosophila melanogaster	73.59	1.61	43	0	0	0	0	0	0	0
Pisaster ochraceus	Huso huso	69.90	20.00	44	0	0	0	0	0	0	0
Plasmodium falciparum	Lecanicillium muscarium	64.36	16.67	45	0	0	0	0	0	0	0
Plasmodium reichenowi	Plasmodium falciparum	97.39	83.33	45	0	0	0	0	0	1	0
Podospora anserina	Penicillium marneffei	76.76	6.06	46	0	0	0	0	0	0	0
Prototheca wickerhamii	Chaetosphaeridium globosum	75.55	10.91	47	0	0	0	0	0	0	0
Reclinomonas americana	Malawimonas jakobiformis	73.06	16.18	48	0	0	0	0	0	0	0
Rhizophydium sp-136	Harpochytrium sp-JEL94	62.39	1.47	49	0	0	0	0	0	0	0
Ancylostoma duodenale	Acanthamoeba castellanii	67.52	7.14	5	0	0	0	0	0	0	0
Necator americanus	Ancylostoma duodenale	83.51	92.86	5	0	0	1	0	0	0	0
Ascaris suum	Ancylostoma duodenale	74.21	73.33	5	0	1	0	0	0	0	0
Caenorhabditis elegans	Ancylostoma duodenale	76.31	85.71	5	0	2	0	0	0	0	0
Cooperia oncophora	Ancylostoma duodenale	79.16	85.71	5	0	3	0	0	0	0	0
Strongyloides stercoralis	Ascaris suum	73.94	64.29	5	0	4	0	0	0	0	0
Brugia malayi	Ascaris suum	65.98	50.00	5	1	0	0	0	0	0	0
Dirofilaria immitis	Brugia malayi	82.40	85.71	5	1	0	1	0	0	0	0
Onchocerca volvulus	Dirofilaria immitis	84.03	92.86	5	1	0	2	0	0	0	0
Rhodomonas salina	Naegleria gruberi	68.05	4.17	50	0	0	0	0	0	0	0
Roboastra europaea	Albinaria caerulea	66.72	20.00	51	0	0	0	0	0	0	0
Saccharomyces cerevisiae-S288c	Saccharomyces castellii	72.51	9.41	52	0	0	0	0	0	0	0

Scenedesmus obliquus	Emiliania huxleyi	66.63	2.38	53	0	0	0	0	0	0	0
Schizophyllum commune	Locusta migratoria	69.99	2.04	54	0	0	0	0	0	0	0
Schizosaccharomyces japonicus	Candida glabrata	66.14	2.53	55	0	0	0	0	0	0	0
Schizosaccharomyces octosporus	Schizosaccharomyces japonicus	66.42	4.55	56	0	0	0	0	0	0	0
Schizosaccharomyces pombe	Schizosaccharomyces octosporus	77.61	15.00	57	0	0	0	0	0	0	0
Spizellomyces punctatus	Phytophthora infestans	64.47	1.61	58	0	0	0	0	0	0	0
Terebratulina retusa	Rattus norvegicus strain BNSsNHsdMCW	72.88	6.25	59	0	0	0	0	0	0	0
Arabidopsis thaliana	Alligator sinensis	67.33	0.28	6	0	0	0	0	0	0	0
Tetrahymena pyriformis	Paramecium aurelia	65.60	10.64	60	0	0	0	0	0	0	0
Tetrahymena thermophila	Tetrahymena pyriformis	80.28	89.36	60	0	0	1	0	0	0	0
Thyropygus sp DVL-2001	Erpetoichthys calabaricus	70.66	20.00	61	0	0	0	0	0	0	0
Tigriopus japonicus	Portunus trituberculatus	64.06	20.00	62	0	0	0	0	0	0	0
Trichinella spiralis	Locusta migratoria	70.40	5.88	63	0	0	0	0	0	0	0
Vargula hilgendorfii	Bactrocera oleae	70.25	6.25	64	0	0	0	0	0	0	0
Venerupis philippinarum	Florometra serratissima	68.95	4.35	65	0	0	0	0	0	0	0
Yarrowia lipolytica	Saccharomyces servazzii	67.71	2.13	66	0	0	0	0	0	0	0
Balaena mysticetus	Arctocephalus forsteri	80.70	5.88	7	0	0	0	0	0	0	0
Beta vulgaris subsp vulgaris	Arabidopsis thaliana	91.78	11.33	8	0	0	0	0	0	0	0
Branchiostoma floridae mitochond	Anopheles gambiae	72.85	6.67	9	0	0	0	0	0	0	0

**Table S4**. Human mitochondrial genomes				**Code**										
**query**	**subject (most similar genome to query genome considering only genomes in rows above the query genome)**	**ANIb %**	**% of aligned fragments**	**M 99.9%**	**N 99.91%**	**O 99.92%**	**P 99.93%**	**Q 99.94%**	**R 99.95%**	**S 99.96%**	**T 99.97%**	**U 99.98%**	**V 99.99%**	**W 99.999%**
AF346982	na	na	na	0	0	0	0	0	0	0	0	0	0	0
EF177410	AF346982	99.9875	94.12	0	0	0	0	0	0	0	0	0	1	0
JF965448	EF177410	99.9875	94.12	0	0	0	0	0	0	0	0	0	10	0
JQ798110	EF177410	99.98125	94.12	0	0	0	0	0	0	0	0	0	11	0
JQ798115	EF177410	99.9875	94.12	0	0	0	0	0	0	0	0	0	12	0
JQ798116	EF177410	99.98125	94.12	0	0	0	0	0	0	0	0	0	13	0
JQ798118	EF177410	99.98125	94.12	0	0	0	0	0	0	0	0	0	14	0
EU258890	EF177410	99.98125	94.12	0	0	0	0	0	0	0	0	0	2	0
JQ798113	EU258890	100	94.12	0	0	0	0	0	0	0	0	0	2	0
EU703624	EF177410	99.98125	94.12	0	0	0	0	0	0	0	0	0	3	0
FJ178379	EU258890	99.98125	94.12	0	0	0	0	0	0	0	0	0	4	0
GU565218	EF177410	99.98188	94.12	0	0	0	0	0	0	0	0	0	5	0
JF831421	EF177410	99.98125	94.12	0	0	0	0	0	0	0	0	0	6	0
JF893457	EU258890	99.9875	94.12	0	0	0	0	0	0	0	0	0	7	0
JF921152	AF346982	99.9875	94.12	0	0	0	0	0	0	0	0	0	8	0
JF937679	EF177410	99.98125	94.12	0	0	0	0	0	0	0	0	0	9	0
EF660960	EF177410	99.975	94.12	0	0	0	0	0	0	0	0	1	0	0
HQ912960	EF660960	99.98125	94.12	0	0	0	0	0	0	0	0	1	1	0
JF937088	EF660960	99.98125	94.12	0	0	0	0	0	0	0	0	1	2	0
JQ798136	EF660960	99.98125	94.12	0	0	0	0	0	0	0	0	1	3	0
EU597536	EF177410	99.975	94.12	0	0	0	0	0	0	0	0	2	0	0
JQ798119	EF177410	99.975	94.12	0	0	0	0	0	0	0	0	3	0	0
JQ798137	HQ912960	99.975	94.12	0	0	0	0	0	0	0	0	4	0	0
FJ238094	EF660960	99.96312	94.12	0	0	0	0	0	0	0	1	0	0	0
JF944823	FJ238094	99.98125	94.12	0	0	0	0	0	0	0	1	0	1	0
GQ304741	EF660960	99.96312	94.12	0	0	0	0	0	0	0	2	0	0	0
HM852862	AF346982	99.96875	94.12	0	0	0	0	0	0	0	3	0	0	0
JF831146	EU258890	99.96875	94.12	0	0	0	0	0	0	0	4	0	0	0
JF903810	JF831146	99.975	94.12	0	0	0	0	0	0	0	4	1	0	0

JN037469	EF177410	99.9625	94.12	0	0	0	0	0	0	0	5	0	0	0
JN202724	EF177410	99.9625	94.12	0	0	0	0	0	0	0	6	0	0	0
JQ798129	EF660960	99.9625	94.12	0	0	0	0	0	0	0	7	0	0	0
JQ798134	EF660960	99.9625	94.12	0	0	0	0	0	0	0	8	0	0	0
AF381985	AF346982	99.95062	94.12	0	0	0	0	0	0	1	0	0	0	0
JQ798114	EF177410	99.95688	94.12	0	0	0	0	0	0	10	0	0	0	0
JQ798117	AF346982	99.95688	94.12	0	0	0	0	0	0	11	0	0	0	0
JQ798135	EF660960	99.95688	94.12	0	0	0	0	0	0	12	0	0	0	0
JQ798138	AF346982	99.95688	94.12	0	0	0	0	0	0	13	0	0	0	0
EU700086	AF346982	99.95688	94.12	0	0	0	0	0	0	2	0	0	0	0
GU944474	EU258890	99.95062	94.12	0	0	0	0	0	0	3	0	0	0	0
HM852818	EF177410	99.95688	94.12	0	0	0	0	0	0	4	0	0	0	0
HM852881	EF660960	99.95062	94.12	0	0	0	0	0	0	5	0	0	0	0
JQ798131	HM852881	99.96875	94.12	0	0	0	0	0	0	5	1	0	0	0
JF707633	AF346982	99.95062	94.12	0	0	0	0	0	0	6	0	0	0	0
JQ798090	EF177410	99.95688	94.12	0	0	0	0	0	0	7	0	0	0	0
JQ798108	EF660960	99.95625	94.12	0	0	0	0	0	0	8	0	0	0	0
JQ798111	AF346982	99.95062	94.12	0	0	0	0	0	0	9	0	0	0	0
JQ798112	JQ798111	100	94.12	0	0	0	0	0	0	9	0	0	0	0
AY714029	AF346982	99.94438	94.12	0	0	0	0	0	1	0	0	0	0	0
JQ798130	EF660960	99.95	94.12	0	0	0	0	0	10	0	0	0	0	0
JQ798132	AF346982	99.94438	94.12	0	0	0	0	0	11	0	0	0	0	0
JQ798133	EF660960	99.95	94.12	0	0	0	0	0	12	0	0	0	0	0
EF060363	AY714029	99.94375	94.12	0	0	0	0	0	2	0	0	0	0	0
EF177442	EF177410	99.95	94.12	0	0	0	0	0	3	0	0	0	0	0
HQ877824	EF177442	99.975	94.12	0	0	0	0	0	3	0	0	1	0	0
EF556186	EF177410	99.94375	94.12	0	0	0	0	0	4	0	0	0	0	0
EU935442	EF556186	99.98125	94.12	0	0	0	0	0	4	0	0	0	1	0
EU935446	EU935442	100	94.12	0	0	0	0	0	4	0	0	0	1	0
EU935448	EU935442	100	94.12	0	0	0	0	0	4	0	0	0	1	0
JQ798127	EU935442	99.99375	94.12	0	0	0	0	0	4	0	0	0	1	1
JN086657	EU935442	99.9875	94.12	0	0	0	0	0	4	0	0	0	2	0
EU007862	EF556186	99.96312	94.12	0	0	0	0	0	4	0	1	0	0	0
JQ798126	JN086657	99.9625	94.12	0	0	0	0	0	4	0	2	0	0	0
EF556188	EF177410	99.945	94.12	0	0	0	0	0	5	0	0	0	0	0
GU048747	EF660960	99.94438	94.12	0	0	0	0	0	6	0	0	0	0	0
JQ798098	GU048747	99.95625	94.12	0	0	0	0	0	6	1	0	0	0	0
HM625693	EF177410	99.94375	94.12	0	0	0	0	0	7	0	0	0	0	0
JQ798139	HM625693	99.95625	94.12	0	0	0	0	0	7	1	0	0	0	0
JN030346	EU258890	99.94438	94.12	0	0	0	0	0	8	0	0	0	0	0
JQ798091	GU048747	99.95	94.12	0	0	0	0	0	9	0	0	0	0	0

AY195767	AF346982	99.93875	94.12	0	0	0	0	1	0	0	0	0	0	0
AY495267	AY195767	99.96875	94.12	0	0	0	0	1	0	0	1	0	0	0
HM852840	AY495277	99.96875	94.12	0	0	0	0	1	0	0	10	0	0	0
HQ287875	AY495273	99.9625	94.12	0	0	0	0	1	0	0	11	0	0	0
HQ287876	HQ287875	99.9875	94.12	0	0	0	0	1	0	0	11	0	1	0
HQ384203	AY495273	99.9625	94.12	0	0	0	0	1	0	0	12	0	0	0
JN037468	AY495273	99.96312	94.12	0	0	0	0	1	0	0	13	0	0	0
JN232198	JN037468	100	94.12	0	0	0	0	1	0	0	13	0	0	0
JN603188	AY495273	99.9625	94.12	0	0	0	0	1	0	0	14	0	0	0
JQ798064	EF660982	99.9625	94.12	0	0	0	0	1	0	0	15	0	0	0
JQ798065	JQ798064	100	94.12	0	0	0	0	1	0	0	15	0	0	0
JQ798069	GU123018	99.96875	94.12	0	0	0	0	1	0	0	16	0	0	0
JQ798078	JN004135	99.96875	94.12	0	0	0	0	1	0	0	17	0	0	0
JQ798088	AY495273	99.96312	94.12	0	0	0	0	1	0	0	18	0	0	0
JQ798089	AY495273	99.9625	94.12	0	0	0	0	1	0	0	19	0	0	0
AY495268	AY195767	99.96875	94.12	0	0	0	0	1	0	0	2	0	0	0
AY495269	AY495268	99.98188	94.12	0	0	0	0	1	0	0	2	0	1	0
AY495273	AY495269	99.99375	94.12	0	0	0	0	1	0	0	2	0	1	1
JN004272	AY495273	100	94.12	0	0	0	0	1	0	0	2	0	1	1
JN032303	AY495273	100	94.12	0	0	0	0	1	0	0	2	0	1	1
JN106403	AY495273	100	94.12	0	0	0	0	1	0	0	2	0	1	1
JN419195	AY495273	100	94.12	0	0	0	0	1	0	0	2	0	1	1
AY495283	AY495273	99.99375	94.12	0	0	0	0	1	0	0	2	0	1	2
HM055613	AY495273	99.99375	94.12	0	0	0	0	1	0	0	2	0	1	3
AY495299	AY495277	99.9875	94.12	0	0	0	0	1	0	0	2	0	10	0
AY495305	AY495299	99.98125	94.12	0	0	0	0	1	0	0	2	0	11	0
AY714016	AY495273	99.9875	94.12	0	0	0	0	1	0	0	2	0	12	0
EF660978	AY495299	99.98125	94.12	0	0	0	0	1	0	0	2	0	13	0
EU007870	AY495273	99.9875	94.12	0	0	0	0	1	0	0	2	0	14	0
EU683970	AY495299	99.9875	94.12	0	0	0	0	1	0	0	2	0	15	0
FJ460542	AY495277	99.9875	94.12	0	0	0	0	1	0	0	2	0	16	0
FJ460555	FJ460542	100	94.12	0	0	0	0	1	0	0	2	0	16	0
GU122998	AY495277	99.9875	94.12	0	0	0	0	1	0	0	2	0	17	0
GU123010	AY495273	99.9875	94.12	0	0	0	0	1	0	0	2	0	18	0
GU123025	AY495273	99.9875	94.12	0	0	0	0	1	0	0	2	0	19	0
AY495270	AY495269	99.98125	94.12	0	0	0	0	1	0	0	2	0	2	0
AY495277	AY495270	99.99375	94.12	0	0	0	0	1	0	0	2	0	2	1
AY495279	AY495277	100	94.12	0	0	0	0	1	0	0	2	0	2	1
EU747356	AY495277	99.99375	94.12	0	0	0	0	1	0	0	2	0	2	2
JQ798086	EU747356	100	94.12	0	0	0	0	1	0	0	2	0	2	2
FJ384436	AY495277	99.99375	94.12	0	0	0	0	1	0	0	2	0	2	3

JN004135	AY495277	99.99375	94.12	0	0	0	0	1	0	0	2	0	2	4
JN024625	AY495277	99.99375	94.12	0	0	0	0	1	0	0	2	0	2	5
GU183768	AY495299	99.98125	94.12	0	0	0	0	1	0	0	2	0	20	0
HM101563	AY495273	99.98125	94.12	0	0	0	0	1	0	0	2	0	21	0
HM852879	AY495277	99.98125	94.12	0	0	0	0	1	0	0	2	0	22	0
HQ231912	AY495277	99.98125	94.12	0	0	0	0	1	0	0	2	0	23	0
HQ399469	AY495273	99.9875	94.12	0	0	0	0	1	0	0	2	0	24	0
JF830105	AY495273	99.98125	94.12	0	0	0	0	1	0	0	2	0	25	0
JF929911	EU683970	99.9875	94.12	0	0	0	0	1	0	0	2	0	26	0
JF939816	EU683970	99.9875	94.12	0	0	0	0	1	0	0	2	0	27	0
JF946696	AY495273	99.98125	94.12	0	0	0	0	1	0	0	2	0	28	0
JF957699	AY495273	99.98125	94.12	0	0	0	0	1	0	0	2	0	29	0
AY495271	AY495270	99.98125	94.12	0	0	0	0	1	0	0	2	0	3	0
JF968593	AY495273	99.9875	94.12	0	0	0	0	1	0	0	2	0	30	0
JF980711	AY495273	99.98125	94.12	0	0	0	0	1	0	0	2	0	31	0
JN024623	AY495275	99.98125	94.12	0	0	0	0	1	0	0	2	0	32	0
JN038393	AY495273	99.9875	94.12	0	0	0	0	1	0	0	2	0	33	0
JN106183	AY495273	99.9875	94.12	0	0	0	0	1	0	0	2	0	34	0
JN315800	AY495273	99.9875	94.12	0	0	0	0	1	0	0	2	0	35	0
JQ798073	AY495273	99.98125	94.12	0	0	0	0	1	0	0	2	0	36	0
JQ798077	AY495273	99.98125	94.12	0	0	0	0	1	0	0	2	0	37	0
JQ798081	AY495273	99.9875	94.12	0	0	0	0	1	0	0	2	0	38	0
JQ798083	AY495273	99.9875	94.12	0	0	0	0	1	0	0	2	0	39	0
AY495274	AY495270	99.9875	94.12	0	0	0	0	1	0	0	2	0	4	0
JQ798084	AY495277	99.98125	94.12	0	0	0	0	1	0	0	2	0	40	0
JQ798085	JF968593	99.98125	94.12	0	0	0	0	1	0	0	2	0	41	0
AY495275	AY495273	99.9875	94.12	0	0	0	0	1	0	0	2	0	5	0
JF937112	AY495275	99.99375	94.12	0	0	0	0	1	0	0	2	0	5	1
JF965447	JF937112	100	94.12	0	0	0	0	1	0	0	2	0	5	1
JN043363	JF937112	100	94.12	0	0	0	0	1	0	0	2	0	5	1
AY495278	AY495273	99.9875	94.12	0	0	0	0	1	0	0	2	0	6	0
AY495280	AY495273	99.98125	94.12	0	0	0	0	1	0	0	2	0	7	0
AY495281	AY495277	99.9875	94.12	0	0	0	0	1	0	0	2	0	8	0
AY495285	AY495273	99.9875	94.12	0	0	0	0	1	0	0	2	0	9	0
AY495272	AY495269	99.975	94.12	0	0	0	0	1	0	0	2	1	0	0
AY495286	AY495272	99.9875	94.12	0	0	0	0	1	0	0	2	1	1	0
EF177444	AY495272	99.9875	94.12	0	0	0	0	1	0	0	2	1	2	0
JF833039	AY495286	99.975	94.12	0	0	0	0	1	0	0	2	10	0	0
JF836084	AY495277	99.975	94.12	0	0	0	0	1	0	0	2	11	0	0
JF937089	JF836084	99.9875	94.12	0	0	0	0	1	0	0	2	11	1	0
JF837334	AY495273	99.975	94.12	0	0	0	0	1	0	0	2	12	0	0

JF900491	EF660982	99.975	94.12	0	0	0	0	1	0	0	2	13	0	0
JF929201	AY495273	99.975	94.12	0	0	0	0	1	0	0	2	14	0	0
JF958132	AY495283	99.975	94.12	0	0	0	0	1	0	0	2	15	0	0
JN032298	EF660978	99.975	94.12	0	0	0	0	1	0	0	2	16	0	0
JN035224	AY495272	99.975	94.12	0	0	0	0	1	0	0	2	17	0	0
JN084060	EF660978	99.975	94.12	0	0	0	0	1	0	0	2	18	0	0
JN126047	AY495277	99.975	94.12	0	0	0	0	1	0	0	2	19	0	0
AY495287	AY495277	99.97562	94.12	0	0	0	0	1	0	0	2	2	0	0
JN419194	AY495277	99.97562	94.12	0	0	0	0	1	0	0	2	20	0	0
JQ798070	JF957699	99.975	94.12	0	0	0	0	1	0	0	2	21	0	0
JQ798071	AY495277	99.975	94.12	0	0	0	0	1	0	0	2	22	0	0
JQ798072	AY495287	99.97562	94.12	0	0	0	0	1	0	0	2	23	0	0
JQ798080	AY495277	99.975	94.12	0	0	0	0	1	0	0	2	24	0	0
JQ798087	JF957699	99.975	94.12	0	0	0	0	1	0	0	2	25	0	0
EF660928	AY495277	99.975	94.12	0	0	0	0	1	0	0	2	3	0	0
EF660982	AY495273	99.975	94.12	0	0	0	0	1	0	0	2	4	0	0
EU494628	EF660982	99.9875	94.12	0	0	0	0	1	0	0	2	4	1	0
JF904739	EF660982	99.9875	94.12	0	0	0	0	1	0	0	2	4	2	0
JQ798066	EF660982	99.9875	94.12	0	0	0	0	1	0	0	2	4	3	0
JQ798067	EF660982	99.9875	94.12	0	0	0	0	1	0	0	2	4	4	0
JQ798068	EF660982	99.98125	94.12	0	0	0	0	1	0	0	2	4	5	0
EU007872	EF660982	99.975	94.12	0	0	0	0	1	0	0	2	5	0	0
EU744542	AY495277	99.975	94.12	0	0	0	0	1	0	0	2	6	0	0
GQ304744	AY495277	99.975	94.12	0	0	0	0	1	0	0	2	7	0	0
HQ638221	AY495277	99.975	94.12	0	0	0	0	1	0	0	2	8	0	0
HQ840646	AY495273	99.975	94.12	0	0	0	0	1	0	0	2	9	0	0
AY495276	AY495270	99.96875	94.12	0	0	0	0	1	0	0	3	0	0	0
EF660922	AY495277	99.96875	94.12	0	0	0	0	1	0	0	4	0	0	0
GU123018	AY495273	99.96875	94.12	0	0	0	0	1	0	0	5	0	0	0
GU170817	AY495277	99.96875	94.12	0	0	0	0	1	0	0	6	0	0	0
HM122274	AY495273	99.96875	94.12	0	0	0	0	1	0	0	7	0	0	0
HM583750	AY495273	99.9625	94.12	0	0	0	0	1	0	0	8	0	0	0
HM852802	EF660982	99.9625	94.12	0	0	0	0	1	0	0	9	0	0	0
AY495282	AY495273	99.95625	94.12	0	0	0	0	1	0	1	0	0	0	0
AY495284	AY495282	99.98125	94.12	0	0	0	0	1	0	1	0	0	1	0
FJ573258	AY495284	99.98125	94.12	0	0	0	0	1	0	1	0	0	2	0
JF891418	AY495284	99.9625	94.12	0	0	0	0	1	0	1	1	0	0	0
JQ798075	AY495277	99.95688	94.12	0	0	0	0	1	0	10	0	0	0	0
JQ798082	AY495277	99.95625	94.12	0	0	0	0	1	0	11	0	0	0	0
AY495298	AY495273	99.95625	94.12	0	0	0	0	1	0	2	0	0	0	0
AY495301	AY495298	99.99375	94.12	0	0	0	0	1	0	2	0	0	0	1

JN383991	AY495301	100	94.12	0	0	0	0	1	0	2	0	0	0	1
AY495304	AY495298	99.99375	94.12	0	0	0	0	1	0	2	0	0	0	2
AY714022	AY495301	99.99375	94.12	0	0	0	0	1	0	2	0	0	0	3
HQ704899	AY495301	99.99375	94.12	0	0	0	0	1	0	2	0	0	0	4
JN120787	HQ704899	100	94.12	0	0	0	0	1	0	2	0	0	0	4
JN630630	HQ704899	100	94.12	0	0	0	0	1	0	2	0	0	0	4
AY495302	AY495301	99.9875	94.12	0	0	0	0	1	0	2	0	0	1	0
FJ656215	AY495301	99.98125	94.12	0	0	0	0	1	0	2	0	0	2	0
GU553285	AY495301	99.98125	94.12	0	0	0	0	1	0	2	0	0	3	0
JF958082	AY495298	99.98125	94.12	0	0	0	0	1	0	2	0	0	4	0
JN012468	HQ704899	99.9875	94.12	0	0	0	0	1	0	2	0	0	5	0
JQ798052	AY495301	99.9875	94.12	0	0	0	0	1	0	2	0	0	6	0
JQ798058	AY495301	99.98125	94.12	0	0	0	0	1	0	2	0	0	7	0
AY495300	AY495298	99.975	94.12	0	0	0	0	1	0	2	0	1	0	0
AY495303	AY495300	99.9875	94.12	0	0	0	0	1	0	2	0	1	1	0
JF833041	AY495298	99.975	94.12	0	0	0	0	1	0	2	0	2	0	0
JQ798053	AY495298	99.97562	94.12	0	0	0	0	1	0	2	0	3	0	0
FJ348180	AY495301	99.96312	94.12	0	0	0	0	1	0	2	1	0	0	0
FJ348191	FJ348180	100	94.12	0	0	0	0	1	0	2	1	0	0	0
FJ348186	FJ348180	99.99375	94.12	0	0	0	0	1	0	2	1	0	0	1
FJ348181	FJ348180	99.9875	94.12	0	0	0	0	1	0	2	1	0	1	0
HM625704	AY495298	99.9625	94.12	0	0	0	0	1	0	2	2	0	0	0
JF927949	AY495298	99.9625	94.12	0	0	0	0	1	0	2	3	0	0	0
JQ798054	AY495301	99.96875	94.12	0	0	0	0	1	0	2	4	0	0	0
DQ523649	AY495277	99.95062	94.12	0	0	0	0	1	0	3	0	0	0	0
DQ523651	DQ523649	100	94.12	0	0	0	0	1	0	3	0	0	0	0
DQ523660	DQ523649	100	94.12	0	0	0	0	1	0	3	0	0	0	0
EF177439	AY495273	99.95625	94.12	0	0	0	0	1	0	4	0	0	0	0
EF660963	DQ523649	99.95688	94.12	0	0	0	0	1	0	5	0	0	0	0
JQ798062	EF660963	99.9875	94.12	0	0	0	0	1	0	5	0	0	1	0
JQ798063	JQ798062	99.98125	94.12	0	0	0	0	1	0	5	0	0	2	0
JQ798060	EF660963	99.975	94.12	0	0	0	0	1	0	5	0	1	0	0
JQ798059	EF660963	99.96312	94.12	0	0	0	0	1	0	5	1	0	0	0
JQ798061	EF660963	99.9625	94.12	0	0	0	0	1	0	5	2	0	0	0
EU492455	EF660982	99.95625	94.12	0	0	0	0	1	0	6	0	0	0	0
EU926622	AY495277	99.95688	94.12	0	0	0	0	1	0	7	0	0	0	0
HM852808	AY495301	99.95625	94.12	0	0	0	0	1	0	8	0	0	0	0
HQ696459	AY495273	99.95688	94.12	0	0	0	0	1	0	9	0	0	0	0
FJ348208	AY495273	99.94438	94.12	0	0	0	0	1	1	0	0	0	0	0
FJ348211	FJ348208	100	94.12	0	0	0	0	1	1	0	0	0	0	0
GU123001	AY495301	99.95	94.12	0	0	0	0	1	2	0	0	0	0	0

HM852781	AY495301	99.94438	94.12	0	0	0	0	1	3	0	0	0	0	0
HM852813	AY495277	99.94438	94.12	0	0	0	0	1	4	0	0	0	0	0
JF930649	AY495298	99.95	94.12	0	0	0	0	1	5	0	0	0	0	0
JN024624	JF930649	99.9625	94.12	0	0	0	0	1	5	0	1	0	0	0
JN048933	JN024624	99.96875	94.12	0	0	0	0	1	5	0	2	0	0	0
JQ798055	JF930649	99.95	94.12	0	0	0	0	1	6	0	0	0	0	0
JQ798056	AY495298	99.94375	94.12	0	0	0	0	1	7	0	0	0	0	0
JQ798057	JQ798056	99.9625	94.12	0	0	0	0	1	7	0	1	0	0	0
JQ798076	GU123010	99.94438	94.12	0	0	0	0	1	8	0	0	0	0	0
AY714037	AY495273	99.93188	94.12	0	0	0	0	2	0	0	0	0	0	0
JQ798104	AY714037	99.95	94.12	0	0	0	0	2	1	0	0	0	0	0
EF660941	EF177410	99.9325	94.12	0	0	0	0	3	0	0	0	0	0	0
JN202494	EF660941	99.9625	94.12	0	0	0	0	3	0	0	1	0	0	0
HM765471	EF660941	99.95625	94.12	0	0	0	0	3	0	1	0	0	0	0
JQ798102	EF660941	99.94375	94.12	0	0	0	0	3	1	0	0	0	0	0
JQ798103	JQ798102	99.94375	94.12	0	0	0	0	3	2	0	0	0	0	0
EU682394	AY495272	99.93312	94.12	0	0	0	0	4	0	0	0	0	0	0
JF979034	EU682394	100	94.12	0	0	0	0	4	0	0	0	0	0	0
JF975728	EU682394	99.9875	94.12	0	0	0	0	4	0	0	0	0	1	0
HM625705	AY495298	99.9375	94.12	0	0	0	0	5	0	0	0	0	0	0
JQ798074	AY495277	99.93812	94.12	0	0	0	0	6	0	0	0	0	0	0
JQ798079	AY495272	99.93125	94.12	0	0	0	0	7	0	0	0	0	0	0
JQ798097	GU048747	99.9375	94.12	0	0	0	0	8	0	0	0	0	0	0
JQ798128	EF177410	99.93812	94.12	0	0	0	0	9	0	0	0	0	0	0
AF382006	AF381985	99.92625	94.12	0	0	0	1	0	0	0	0	0	0	0
AY495289	AF382006	100	94.12	0	0	0	1	0	0	0	0	0	0	0
GU122980	AF382006	100	94.12	0	0	0	1	0	0	0	0	0	0	0
HQ167734	AF382006	100	94.12	0	0	0	1	0	0	0	0	0	0	0
JQ797977	AF382006	100	94.12	0	0	0	1	0	0	0	0	0	0	0
JQ797978	AF382006	100	94.12	0	0	0	1	0	0	0	0	0	0	0
JQ797979	AF382006	100	94.12	0	0	0	1	0	0	0	0	0	0	0
JQ797980	AF382006	100	94.12	0	0	0	1	0	0	0	0	0	0	0
JQ797981	AF382006	100	94.12	0	0	0	1	0	0	0	0	0	0	0
AY495288	AF382006	99.99375	94.12	0	0	0	1	0	0	0	0	0	0	1
JN089342	AF382006	99.99375	94.12	0	0	0	1	0	0	0	0	0	0	10
JQ797982	AF382006	99.99375	94.12	0	0	0	1	0	0	0	0	0	0	11
JQ797987	AF382006	99.99375	94.12	0	0	0	1	0	0	0	0	0	0	12
JQ797988	AF382006	99.99375	94.12	0	0	0	1	0	0	0	0	0	0	13
JQ797989	AF382006	99.99375	94.12	0	0	0	1	0	0	0	0	0	0	14
JQ798002	AF382006	99.99375	94.12	0	0	0	1	0	0	0	0	0	0	15
AY495293	AF382006	99.99375	94.12	0	0	0	1	0	0	0	0	0	0	2

AY495297	AF382006	99.99375	94.12	0	0	0	1	0	0	0	0	0	0	3
AY714015	AF382006	99.99375	94.12	0	0	0	1	0	0	0	0	0	0	4
FJ348197	AF382006	99.99375	94.12	0	0	0	1	0	0	0	0	0	0	5
GU365880	AF382006	99.99375	94.12	0	0	0	1	0	0	0	0	0	0	6
JF833038	AF382006	99.99375	94.12	0	0	0	1	0	0	0	0	0	0	7
JF905569	AF382006	99.99375	94.12	0	0	0	1	0	0	0	0	0	0	8
JF930640	AF382006	99.99375	94.12	0	0	0	1	0	0	0	0	0	0	9
AY495291	AF382006	99.9875	94.12	0	0	0	1	0	0	0	0	0	1	0
GQ304742	AF382006	99.9875	94.12	0	0	0	1	0	0	0	0	0	10	0
GU944473	AF382006	99.9875	94.12	0	0	0	1	0	0	0	0	0	11	0
HM184912	AF382006	99.9875	94.12	0	0	0	1	0	0	0	0	0	12	0
JF830257	AF382006	99.9875	94.12	0	0	0	1	0	0	0	0	0	13	0
JF830642	AF382006	99.98125	94.12	0	0	0	1	0	0	0	0	0	14	0
JF833040	AF382006	99.9875	94.12	0	0	0	1	0	0	0	0	0	15	0
JF836809	AF382006	99.9875	94.12	0	0	0	1	0	0	0	0	0	16	0
JF837819	AF382006	99.9875	94.12	0	0	0	1	0	0	0	0	0	17	0
JF905566	AF382006	99.98125	94.12	0	0	0	1	0	0	0	0	0	18	0
JF905570	AF382006	99.98125	94.12	0	0	0	1	0	0	0	0	0	19	0
AY495292	AF382006	99.9875	94.12	0	0	0	1	0	0	0	0	0	2	0
JF926125	JF905570	99.9875	94.12	0	0	0	1	0	0	0	0	0	20	0
JF929200	AF382006	99.9875	94.12	0	0	0	1	0	0	0	0	0	21	0
JF979131	AF382006	99.9875	94.12	0	0	0	1	0	0	0	0	0	22	0
JQ797983	JQ797982	99.98125	94.12	0	0	0	1	0	0	0	0	0	23	0
JQ797984	AF382006	99.9875	94.12	0	0	0	1	0	0	0	0	0	24	0
JQ797985	AF382006	99.98125	94.12	0	0	0	1	0	0	0	0	0	25	0
JQ797990	AF382006	99.9875	94.12	0	0	0	1	0	0	0	0	0	26	0
JQ797996	AF382006	99.98125	94.12	0	0	0	1	0	0	0	0	0	27	0
JQ797997	AF382006	99.98125	94.12	0	0	0	1	0	0	0	0	0	28	0
JQ797999	AF382006	99.9875	94.12	0	0	0	1	0	0	0	0	0	29	0
AY495294	AF382006	99.98125	94.12	0	0	0	1	0	0	0	0	0	3	0
EU882063	AY495294	100	94.12	0	0	0	1	0	0	0	0	0	3	0
JQ798000	AF382006	99.98125	94.12	0	0	0	1	0	0	0	0	0	30	0
JQ798001	AF382006	99.98125	94.12	0	0	0	1	0	0	0	0	0	31	0
JQ798003	JF979131	99.9875	94.12	0	0	0	1	0	0	0	0	0	32	0
JQ798004	JF979131	99.9875	94.12	0	0	0	1	0	0	0	0	0	33	0
AY495295	AY495293	99.9875	94.12	0	0	0	1	0	0	0	0	0	4	0
AY495296	AF382006	99.98125	94.12	0	0	0	1	0	0	0	0	0	5	0
JF832384	AY495296	99.99375	94.12	0	0	0	1	0	0	0	0	0	5	1
EF177406	AF382006	99.98125	94.12	0	0	0	1	0	0	0	0	0	6	0
EF645646	AY495296	99.98125	94.12	0	0	0	1	0	0	0	0	0	7	0
EU367994	AF382006	99.98125	94.12	0	0	0	1	0	0	0	0	0	8	0

EU597578	AF382006	99.9875	94.12	0	0	0	1	0	0	0	0	0	9	0
AY495290	AF382006	99.975	94.12	0	0	0	1	0	0	0	0	1	0	0
DQ358975	AY495291	99.975	94.12	0	0	0	1	0	0	0	0	2	0	0
JQ797991	AF382006	99.975	94.12	0	0	0	1	0	0	0	0	3	0	0
JQ797998	AF382006	99.975	94.12	0	0	0	1	0	0	0	0	4	0	0
JQ798007	EU367994	99.975	94.12	0	0	0	1	0	0	0	0	5	0	0
JQ798020	JQ798007	99.97562	94.12	0	0	0	1	0	0	0	0	6	0	0
AY714036	AF382006	99.96875	94.12	0	0	0	1	0	0	0	1	0	0	0
JQ797994	AY714036	99.98125	94.12	0	0	0	1	0	0	0	1	0	1	0
JQ797995	JQ797994	99.9875	94.12	0	0	0	1	0	0	0	1	0	2	0
JQ797993	AY714036	99.975	94.12	0	0	0	1	0	0	0	1	1	0	0
JQ798008	JQ798007	99.96938	94.12	0	0	0	1	0	0	0	10	0	0	0
JQ798009	JQ798008	99.98125	94.12	0	0	0	1	0	0	0	10	0	1	0
JQ798012	JQ798007	99.96312	94.12	0	0	0	1	0	0	0	11	0	0	0
JQ798015	AF382006	99.9625	94.12	0	0	0	1	0	0	0	12	0	0	0
JQ798016	JQ798007	99.96875	94.12	0	0	0	1	0	0	0	13	0	0	0
JQ798019	JQ797986	99.96312	94.12	0	0	0	1	0	0	0	14	0	0	0
JQ798028	JQ798007	99.9625	94.12	0	0	0	1	0	0	0	15	0	0	0
JQ798038	JQ798037	99.9625	94.12	0	0	0	1	0	0	0	16	0	0	0
JQ798049	JQ798046	99.96875	94.12	0	0	0	1	0	0	0	17	0	0	0
EF177409	AF382006	99.96875	94.12	0	0	0	1	0	0	0	2	0	0	0
EU979542	AF382006	99.9625	94.12	0	0	0	1	0	0	0	3	0	0	0
FJ480957	AF382006	99.9625	94.12	0	0	0	1	0	0	0	4	0	0	0
JQ798006	FJ480957	99.9875	94.12	0	0	0	1	0	0	0	4	0	1	0
FJ878777	EF177409	99.96875	94.12	0	0	0	1	0	0	0	5	0	0	0
JQ798046	FJ878777	99.99375	94.12	0	0	0	1	0	0	0	5	0	0	1
JQ798037	FJ878777	99.98125	94.12	0	0	0	1	0	0	0	5	0	1	0
JQ798043	FJ878777	99.9875	94.12	0	0	0	1	0	0	0	5	0	2	0
JQ798044	JQ798043	99.9875	94.12	0	0	0	1	0	0	0	5	0	3	0
JQ798045	JQ798044	100	94.12	0	0	0	1	0	0	0	5	0	3	0
JQ798047	JQ798046	99.98125	94.12	0	0	0	1	0	0	0	5	0	4	0
JQ798048	JQ798046	99.9875	94.12	0	0	0	1	0	0	0	5	0	5	0
JQ798050	JQ798046	99.98125	94.12	0	0	0	1	0	0	0	5	0	6	0
JQ798051	JQ798046	99.98125	94.12	0	0	0	1	0	0	0	5	0	7	0
JQ798039	FJ878777	99.975	94.12	0	0	0	1	0	0	0	5	1	0	0
JQ798040	JQ798039	99.98188	94.12	0	0	0	1	0	0	0	5	1	1	0
JQ798042	FJ878777	99.975	94.12	0	0	0	1	0	0	0	5	2	0	0
HQ384202	AF382006	99.96875	94.12	0	0	0	1	0	0	0	6	0	0	0
JN021256	AY495293	99.96312	94.12	0	0	0	1	0	0	0	7	0	0	0
JN127414	JN021256	100	94.12	0	0	0	1	0	0	0	7	0	0	0
JQ797986	AF382006	99.96875	94.12	0	0	0	1	0	0	0	8	0	0	0

JQ797992	AF382006	99.9625	94.12	0	0	0	1	0	0	0	9	0	0	0
EF661000	AF382006	99.95625	94.12	0	0	0	1	0	0	1	0	0	0	0
JQ798024	EF177409	99.95625	94.12	0	0	0	1	0	0	10	0	0	0	0
JQ798029	JQ798007	99.95062	94.12	0	0	0	1	0	0	11	0	0	0	0
JQ798031	AY495296	99.95062	94.12	0	0	0	1	0	0	12	0	0	0	0
JQ798033	EF177409	99.95625	94.12	0	0	0	1	0	0	13	0	0	0	0
JQ798034	JQ798033	99.9875	94.12	0	0	0	1	0	0	13	0	0	1	0
EU007876	AF382006	99.95625	94.12	0	0	0	1	0	0	2	0	0	0	0
EU369395	EF177409	99.95625	94.12	0	0	0	1	0	0	3	0	0	0	0
JN104727	EU369395	99.975	94.12	0	0	0	1	0	0	3	0	1	0	0
EU935435	EF177409	99.95625	94.12	0	0	0	1	0	0	4	0	0	0	0
EU935441	EU935435	100	94.12	0	0	0	1	0	0	4	0	0	0	0
EU935447	EU935435	100	94.12	0	0	0	1	0	0	4	0	0	0	0
EU935444	EU935435	99.99375	94.12	0	0	0	1	0	0	4	0	0	0	1
EU935452	EU935444	100	94.12	0	0	0	1	0	0	4	0	0	0	1
JQ798026	EU935435	99.975	94.12	0	0	0	1	0	0	4	0	1	0	0
JQ798027	EU935435	99.96938	94.12	0	0	0	1	0	0	4	1	0	0	0
JQ797975	FJ878777	99.95625	94.12	0	0	0	1	0	0	5	0	0	0	0
JQ797976	AF382006	99.95625	94.12	0	0	0	1	0	0	6	0	0	0	0
JQ798014	JQ798007	99.95688	94.12	0	0	0	1	0	0	7	0	0	0	0
JQ798018	EF177409	99.95625	94.12	0	0	0	1	0	0	8	0	0	0	0
JQ798022	AY495296	99.95688	94.12	0	0	0	1	0	0	9	0	0	0	0
EF177441	AF382006	99.94375	94.12	0	0	0	1	0	1	0	0	0	0	0
JQ798010	EF177441	99.975	94.12	0	0	0	1	0	1	0	0	1	0	0
EF660961	EF177441	99.9625	94.12	0	0	0	1	0	1	0	1	0	0	0
JQ798011	EF660961	99.975	94.12	0	0	0	1	0	1	0	1	1	0	0
FJ348220	EF660961	99.96875	94.12	0	0	0	1	0	1	0	2	0	0	0
JQ798036	JQ798034	99.94375	94.12	0	0	0	1	0	10	0	0	0	0	0
JF937680	EF177409	99.94438	94.12	0	0	0	1	0	2	0	0	0	0	0
JN083377	AF382006	99.94438	94.12	0	0	0	1	0	3	0	0	0	0	0
JQ798005	AF382006	99.95	94.12	0	0	0	1	0	4	0	0	0	0	0
JQ798017	FJ878777	99.94375	94.12	0	0	0	1	0	5	0	0	0	0	0
JQ798023	FJ878777	99.95	94.12	0	0	0	1	0	6	0	0	0	0	0
JQ798030	EF177409	99.95	94.12	0	0	0	1	0	7	0	0	0	0	0
JQ798032	EF177409	99.95	94.12	0	0	0	1	0	8	0	0	0	0	0
JQ798035	JF929200	99.945	94.12	0	0	0	1	0	9	0	0	0	0	0
HM852798	DQ358975	99.9375	94.12	0	0	0	1	1	0	0	0	0	0	0
JF831941	AY495293	99.9375	94.12	0	0	0	1	2	0	0	0	0	0	0
JQ798041	JQ798040	99.93938	94.12	0	0	0	1	3	0	0	0	0	0	0
EU935439	AF382006	99.92562	94.12	0	0	0	2	0	0	0	0	0	0	0
EU935455	EU935439	99.9875	94.12	0	0	0	2	0	0	0	0	0	1	0

JQ798021	EU935439	99.9875	94.12	0	0	0	2	0	0	0	0	0	2	0
HM852766	EU744542	99.92625	94.12	0	0	0	3	0	0	0	0	0	0	0
HM852810	HM852808	99.92625	94.12	0	0	0	4	0	0	0	0	0	0	0
HM852899	EF660960	99.92625	94.12	0	0	0	5	0	0	0	0	0	0	0
JQ798109	HM852899	99.95688	94.12	0	0	0	5	0	0	1	0	0	0	0
JQ798025	JF929200	99.92562	94.12	0	0	0	6	0	0	0	0	0	0	0
AY195745	AF346982	99.92	94.12	0	0	1	0	0	0	0	0	0	0	0
HQ917079	AY195745	99.975	94.12	0	0	1	0	0	0	0	0	1	0	0
JF340114	HQ917079	99.93875	94.12	0	0	1	0	1	0	0	0	0	0	0
GU123028	GU123010	99.92	94.12	0	0	2	0	0	0	0	0	0	0	0
HQ287877	GU123028	99.93812	94.12	0	0	2	0	1	0	0	0	0	0	0
GU932663	GU123028	99.91375	94.12	0	0	3	0	0	0	0	0	0	0	0
JF905568	GU932663	100	94.12	0	0	3	0	0	0	0	0	0	0	0
JF906520	GU932663	100	94.12	0	0	3	0	0	0	0	0	0	0	0
JF926842	GU932663	100	94.12	0	0	3	0	0	0	0	0	0	0	0
JN203494	GU932663	99.99375	94.12	0	0	3	0	0	0	0	0	0	0	1
JQ798123	GU932663	99.99375	94.12	0	0	3	0	0	0	0	0	0	0	2
JF940522	GU932663	99.98125	94.12	0	0	3	0	0	0	0	0	0	1	0
JQ798122	GU932663	99.9875	94.12	0	0	3	0	0	0	0	0	0	2	0
JQ798121	GU932663	99.97562	94.12	0	0	3	0	0	0	0	0	1	0	0
JQ798120	GU932663	99.95625	94.12	0	0	3	0	0	0	1	0	0	0	0
HQ286590	GU932663	99.92562	94.12	0	0	3	1	0	0	0	0	0	0	0
JQ798125	HQ286590	99.975	94.12	0	0	3	1	0	0	0	0	1	0	0
JQ798124	HQ286590	99.95688	94.12	0	0	3	1	0	0	1	0	0	0	0
JF960209	HQ286590	99.93875	94.12	0	0	3	1	1	0	0	0	0	0	0
JN084792	JF960209	99.94375	94.12	0	0	3	1	1	1	0	0	0	0	0
JN034044	HQ286590	99.93812	94.12	0	0	3	1	2	0	0	0	0	0	0
HM765473	EF660960	99.91375	94.12	0	0	4	0	0	0	0	0	0	0	0
JQ798105	HM765473	99.94438	94.12	0	0	4	0	0	1	0	0	0	0	0
JQ798106	JQ798105	99.975	94.12	0	0	4	0	0	1	0	0	1	0	0
JQ798107	JQ798106	100	94.12	0	0	4	0	0	1	0	0	1	0	0
HM852775	AF382006	99.92	94.12	0	0	5	0	0	0	0	0	0	0	0
JQ798013	HM852775	99.95	94.12	0	0	5	0	0	1	0	0	0	0	0
HM852796	EF177410	99.91438	94.12	0	0	6	0	0	0	0	0	0	0	0
JQ798101	EF177410	99.91313	94.12	0	0	7	0	0	0	0	0	0	0	0
DQ437577	AY714037	99.90125	94.12	0	1	0	0	0	0	0	0	0	0	0
DQ523629	AF346982	99.90125	94.12	0	2	0	0	0	0	0	0	0	0	0
DQ523633	DQ523629	100	94.12	0	2	0	0	0	0	0	0	0	0	0
DQ523667	DQ523629	99.99375	94.12	0	2	0	0	0	0	0	0	0	0	1
JQ798095	DQ523629	99.98125	94.12	0	2	0	0	0	0	0	0	0	1	0
JQ798096	JQ798095	99.975	94.12	0	2	0	0	0	0	0	0	1	0	0

JF833037	DQ523629	99.93812	94.12	0	2	0	0	1	0	0	0	0	0	0
JF892552	JF833037	99.95625	94.12	0	2	0	0	1	0	1	0	0	0	0
JF951723	JF892552	99.975	94.12	0	2	0	0	1	0	1	0	1	0	0
JN252308	JF951723	99.9875	94.12	0	2	0	0	1	0	1	0	1	1	0
JN580589	JF951723	99.9875	94.12	0	2	0	0	1	0	1	0	1	2	0
JQ798092	JN580589	99.99375	94.12	0	2	0	0	1	0	1	0	1	2	1
JQ798093	JF951723	99.9875	94.12	0	2	0	0	1	0	1	0	1	3	0
JQ798094	JF951723	99.9875	94.12	0	2	0	0	1	0	1	0	1	4	0
JQ798099	EF660941	99.90688	94.12	0	3	0	0	0	0	0	0	0	0	0
JQ798100	JQ798099	99.94438	94.12	0	3	0	0	0	1	0	0	0	0	0
AF346983	AF346982	99.82062	94.12	1	0	0	0	0	0	0	0	0	0	0
AY495200	AF346983	100	94.12	1	0	0	0	0	0	0	0	0	0	0
GU123008	AF346983	100	94.12	1	0	0	0	0	0	0	0	0	0	0
AY495197	AF346983	99.99375	94.12	1	0	0	0	0	0	0	0	0	0	1
AY195774	AF346983	99.98125	94.12	1	0	0	0	0	0	0	0	0	1	0
AY495225	AY495221	99.9875	94.12	1	0	0	0	0	0	0	0	0	10	0
EF459669	AY495212	99.98125	94.12	1	0	0	0	0	0	0	0	0	11	0
EF583178	EF459669	100	94.12	1	0	0	0	0	0	0	0	0	11	0
EU547187	EF459669	100	94.12	1	0	0	0	0	0	0	0	0	11	0
JQ797849	EF459669	99.99375	94.12	1	0	0	0	0	0	0	0	0	11	1
JQ797853	JQ797849	100	94.12	1	0	0	0	0	0	0	0	0	11	1
JQ797850	JQ797849	99.99375	94.12	1	0	0	0	0	0	0	0	0	11	2
EF660952	AY495221	99.98125	94.12	1	0	0	0	0	0	0	0	0	12	0
EU915476	AY495197	99.9875	94.12	1	0	0	0	0	0	0	0	0	13	0
FJ499471	AY495221	99.98125	94.12	1	0	0	0	0	0	0	0	0	14	0
FJ538285	AY495197	99.9875	94.12	1	0	0	0	0	0	0	0	0	15	0
GU808335	AF346983	99.98125	94.12	1	0	0	0	0	0	0	0	0	16	0
HQ384195	AF346983	99.98125	94.12	1	0	0	0	0	0	0	0	0	17	0
JN663354	AY495218	99.9875	94.12	1	0	0	0	0	0	0	0	0	18	0
JQ797799	AY495221	99.98125	94.12	1	0	0	0	0	0	0	0	0	19	0
AY495203	AY495197	99.98125	94.12	1	0	0	0	0	0	0	0	0	2	0
JQ797801	AY495221	99.98125	94.12	1	0	0	0	0	0	0	0	0	20	0
JQ797804	AY495221	99.98125	94.12	1	0	0	0	0	0	0	0	0	21	0
JQ797806	AY495221	99.98125	94.12	1	0	0	0	0	0	0	0	0	22	0
JQ797809	AY495218	99.98125	94.12	1	0	0	0	0	0	0	0	0	23	0
JQ797815	JQ797809	99.98125	94.12	1	0	0	0	0	0	0	0	0	24	0
JQ797822	JQ797818	99.98125	94.12	1	0	0	0	0	0	0	0	0	25	0
JQ797841	AY195774	99.98125	94.12	1	0	0	0	0	0	0	0	0	26	0
JQ797852	JQ797849	99.9875	94.12	1	0	0	0	0	0	0	0	0	27	0
JQ797869	JQ797818	99.9875	94.12	1	0	0	0	0	0	0	0	0	28	0
AY495206	AF346983	99.98125	94.12	1	0	0	0	0	0	0	0	0	3	0

AY495209	AY495206	99.98125	94.12	1	0	0	0	0	0	0	0	0	4	0
AY495212	AY495203	99.98125	94.12	1	0	0	0	0	0	0	0	0	5	0
AY495218	AY495203	99.9875	94.12	1	0	0	0	0	0	0	0	0	6	0
AY495230	AY495218	100	94.12	1	0	0	0	0	0	0	0	0	6	0
AY495221	AY495218	99.99375	94.12	1	0	0	0	0	0	0	0	0	6	1
AY495228	AY495221	100	94.12	1	0	0	0	0	0	0	0	0	6	1
HM590710	AY495221	100	94.12	1	0	0	0	0	0	0	0	0	6	1
HM803933	AY495221	100	94.12	1	0	0	0	0	0	0	0	0	6	1
HM856585	AY495221	100	94.12	1	0	0	0	0	0	0	0	0	6	1
JQ797816	AY495221	99.99375	94.12	1	0	0	0	0	0	0	0	0	6	2
JQ797818	AY495218	99.99375	94.12	1	0	0	0	0	0	0	0	0	6	3
AY495219	AY495218	99.98125	94.12	1	0	0	0	0	0	0	0	0	7	0
GU123042	AY495219	99.99375	94.12	1	0	0	0	0	0	0	0	0	7	1
AY495223	AY495221	99.98125	94.12	1	0	0	0	0	0	0	0	0	8	0
AY495224	AY495219	99.9875	94.12	1	0	0	0	0	0	0	0	0	9	0
AY495201	AY495197	99.975	94.12	1	0	0	0	0	0	0	0	1	0	0
AY495207	AY495201	99.9875	94.12	1	0	0	0	0	0	0	0	1	1	0
EF660962	AY495201	99.98125	94.12	1	0	0	0	0	0	0	0	1	2	0
JQ797826	EF660962	99.98125	94.12	1	0	0	0	0	0	0	0	1	3	0
JQ797828	JQ797826	99.98125	94.12	1	0	0	0	0	0	0	0	1	4	0
JQ797831	JQ797828	99.98125	94.12	1	0	0	0	0	0	0	0	1	5	0
JQ797832	JQ797828	99.9875	94.12	1	0	0	0	0	0	0	0	1	6	0
JQ797834	JQ797831	99.98125	94.12	1	0	0	0	0	0	0	0	1	7	0
DQ358973	AY495221	99.97562	94.12	1	0	0	0	0	0	0	0	10	0	0
DQ787109	AY495218	99.975	94.12	1	0	0	0	0	0	0	0	11	0	0
EU155191	AY495221	99.975	94.12	1	0	0	0	0	0	0	0	12	0	0
EU284668	AY495218	99.97562	94.12	1	0	0	0	0	0	0	0	13	0	0
FJ348222	EU284668	99.99375	94.12	1	0	0	0	0	0	0	0	13	0	1
FJ348153	AY495206	99.97562	94.12	1	0	0	0	0	0	0	0	14	0	0
FJ348154	FJ348153	99.99375	94.12	1	0	0	0	0	0	0	0	14	0	1
FJ348155	FJ348154	100	94.12	1	0	0	0	0	0	0	0	14	0	1
JQ797862	FJ348153	99.98125	94.12	1	0	0	0	0	0	0	0	14	1	0
HM016082	AF346983	99.975	94.12	1	0	0	0	0	0	0	0	15	0	0
HM776018	AY495203	99.975	94.12	1	0	0	0	0	0	0	0	16	0	0
HQ154118	AY495221	99.975	94.12	1	0	0	0	0	0	0	0	17	0	0
JN635300	AY495212	99.975	94.12	1	0	0	0	0	0	0	0	18	0	0
JQ797802	AY495218	99.975	94.12	1	0	0	0	0	0	0	0	19	0	0
AY495205	AF346983	99.975	94.12	1	0	0	0	0	0	0	0	2	0	0
JQ797803	AY495221	99.975	94.12	1	0	0	0	0	0	0	0	20	0	0
JQ797805	AY495221	99.975	94.12	1	0	0	0	0	0	0	0	21	0	0
JQ797808	AY495221	99.975	94.12	1	0	0	0	0	0	0	0	22	0	0

JQ797817	AY495218	99.97562	94.12	1	0	0	0	0	0	0	0	23	0	0
JQ797830	JQ797828	99.975	94.12	1	0	0	0	0	0	0	0	24	0	0
JQ797833	JQ797831	99.975	94.12	1	0	0	0	0	0	0	0	25	0	0
JQ797840	AY495197	99.975	94.12	1	0	0	0	0	0	0	0	26	0	0
JQ797847	EF459669	99.97562	94.12	1	0	0	0	0	0	0	0	27	0	0
JQ797848	JQ797847	99.99375	94.12	1	0	0	0	0	0	0	0	27	0	1
JQ797854	JQ797848	99.99375	94.12	1	0	0	0	0	0	0	0	27	0	2
JQ797875	JQ797848	99.99375	94.12	1	0	0	0	0	0	0	0	27	0	3
JQ797851	JQ797848	99.9875	94.12	1	0	0	0	0	0	0	0	27	1	0
JQ797881	JQ797816	99.975	94.12	1	0	0	0	0	0	0	0	28	0	0
AY495211	AY495201	99.975	94.12	1	0	0	0	0	0	0	0	3	0	0
HM560728	AY495211	100	94.12	1	0	0	0	0	0	0	0	3	0	0
JQ797824	AY495211	99.98188	94.12	1	0	0	0	0	0	0	0	3	1	0
JQ797825	JQ797824	99.9875	94.12	1	0	0	0	0	0	0	0	3	2	0
AY495213	AY495211	99.975	94.12	1	0	0	0	0	0	0	0	4	0	0
AY495217	AY495213	100	94.12	1	0	0	0	0	0	0	0	4	0	0
AY495220	AY495218	99.975	94.12	1	0	0	0	0	0	0	0	5	0	0
AY495222	AY495203	99.975	94.12	1	0	0	0	0	0	0	0	6	0	0
AY495226	AY495221	99.975	94.12	1	0	0	0	0	0	0	0	7	0	0
AY495229	AY495218	99.975	94.12	1	0	0	0	0	0	0	0	8	0	0
AY665667	AF346983	99.975	94.12	1	0	0	0	0	0	0	0	9	0	0
AY495196	AF346983	99.9625	94.12	1	0	0	0	0	0	0	1	0	0	0
FJ190383	AY495219	99.9625	94.12	1	0	0	0	0	0	0	10	0	0	0
FJ445407	EF660962	99.96875	94.12	1	0	0	0	0	0	0	11	0	0	0
HM627754	FJ445407	99.99375	94.12	1	0	0	0	0	0	0	11	0	0	1
HM026752	FJ445407	99.98125	94.12	1	0	0	0	0	0	0	11	0	1	0
HQ696458	FJ445407	99.9875	94.12	1	0	0	0	0	0	0	11	0	2	0
FJ499472	AY495221	99.96938	94.12	1	0	0	0	0	0	0	12	0	0	0
FJ603099	EF660962	99.96875	94.12	1	0	0	0	0	0	0	13	0	0	0
GQ304743	AY495201	99.9625	94.12	1	0	0	0	0	0	0	14	0	0	0
GU592032	AY495206	99.9625	94.12	1	0	0	0	0	0	0	15	0	0	0
GU592047	GU592032	99.98125	94.12	1	0	0	0	0	0	0	15	0	1	0
HM765470	GU592047	99.975	94.12	1	0	0	0	0	0	0	15	1	0	0
GU906781	AY495203	99.96875	94.12	1	0	0	0	0	0	0	16	0	0	0
JQ797864	GU906781	99.98125	94.12	1	0	0	0	0	0	0	16	0	1	0
JQ797867	JQ797864	99.98125	94.12	1	0	0	0	0	0	0	16	0	2	0
JQ797865	JQ797864	99.97562	94.12	1	0	0	0	0	0	0	16	1	0	0
HM159445	AY495212	99.96312	94.12	1	0	0	0	0	0	0	17	0	0	0
HM775495	AY495203	99.96875	94.12	1	0	0	0	0	0	0	18	0	0	0
HQ260985	AY495221	99.96875	94.12	1	0	0	0	0	0	0	19	0	0	0
AY495199	AF346983	99.96875	94.12	1	0	0	0	0	0	0	2	0	0	0

HQ287874	AY495199	99.9625	94.12	1	0	0	0	0	0	0	20	0	0	0
HQ709108	AY495201	99.96312	94.12	1	0	0	0	0	0	0	21	0	0	0
HQ709168	AY495201	99.96875	94.12	1	0	0	0	0	0	0	22	0	0	0
JF703252	AY495201	99.96875	94.12	1	0	0	0	0	0	0	23	0	0	0
JN635302	AY495212	99.96938	94.12	1	0	0	0	0	0	0	24	0	0	0
JQ797800	AY495218	99.9625	94.12	1	0	0	0	0	0	0	25	0	0	0
JQ797811	AY495221	99.96938	94.12	1	0	0	0	0	0	0	26	0	0	0
JQ797812	JQ797811	99.9875	94.12	1	0	0	0	0	0	0	26	0	1	0
JQ797813	JQ797812	100	94.12	1	0	0	0	0	0	0	26	0	1	0
JQ797814	JQ797812	99.98125	94.12	1	0	0	0	0	0	0	26	0	2	0
JQ797819	FJ190383	99.96938	94.12	1	0	0	0	0	0	0	27	0	0	0
JQ797823	AY495206	99.96875	94.12	1	0	0	0	0	0	0	28	0	0	0
JQ797827	AY495201	99.96875	94.12	1	0	0	0	0	0	0	29	0	0	0
AY495214	AY495201	99.96875	94.12	1	0	0	0	0	0	0	3	0	0	0
JQ797829	HQ709108	99.96938	94.12	1	0	0	0	0	0	0	30	0	0	0
JQ797835	JQ797816	99.96875	94.12	1	0	0	0	0	0	0	31	0	0	0
JQ797836	JQ797816	99.9625	94.12	1	0	0	0	0	0	0	32	0	0	0
JQ797837	DQ523659	99.9625	94.12	1	0	0	0	0	0	0	33	0	0	0
JQ797838	JQ797837	99.99375	94.12	1	0	0	0	0	0	0	33	0	0	1
JQ797839	AY495197	99.96875	94.12	1	0	0	0	0	0	0	34	0	0	0
JQ797843	AY495212	99.96875	94.12	1	0	0	0	0	0	0	35	0	0	0
JQ797844	JN635302	99.9625	94.12	1	0	0	0	0	0	0	36	0	0	0
JQ797845	JN635300	99.96938	94.12	1	0	0	0	0	0	0	37	0	0	0
JQ797863	GU906781	99.96312	94.12	1	0	0	0	0	0	0	38	0	0	0
JQ797868	JQ797863	99.99375	94.12	1	0	0	0	0	0	0	38	0	0	1
JQ797866	JQ797864	99.96312	94.12	1	0	0	0	0	0	0	39	0	0	0
AY495215	AY495201	99.9625	94.12	1	0	0	0	0	0	0	4	0	0	0
JQ797870	JQ797816	99.96875	94.12	1	0	0	0	0	0	0	40	0	0	0
JQ797882	JQ797818	99.9625	94.12	1	0	0	0	0	0	0	41	0	0	0
JQ797884	HM775495	99.96938	94.12	1	0	0	0	0	0	0	42	0	0	0
AY495227	AY495218	99.9625	94.12	1	0	0	0	0	0	0	5	0	0	0
DQ523659	AY495206	99.9625	94.12	1	0	0	0	0	0	0	6	0	0	0
EF660915	AY495212	99.96875	94.12	1	0	0	0	0	0	0	7	0	0	0
EU007859	AY495212	99.96938	94.12	1	0	0	0	0	0	0	8	0	0	0
JQ797842	EU007859	99.98125	94.12	1	0	0	0	0	0	0	8	0	1	0
JQ797871	JQ797842	99.98125	94.12	1	0	0	0	0	0	0	8	0	2	0
EU915479	AY495218	99.96875	94.12	1	0	0	0	0	0	0	9	0	0	0
AY195754	AF346983	99.95625	94.12	1	0	0	0	0	0	1	0	0	0	0
HM625690	AY195754	99.98125	94.12	1	0	0	0	0	0	1	0	0	1	0
JQ797798	HM625690	99.98125	94.12	1	0	0	0	0	0	1	0	0	2	0
EF177431	AY195754	99.975	94.12	1	0	0	0	0	0	1	0	1	0	0

HQ384194	HM625690	99.975	94.12	1	0	0	0	0	0	1	0	2	0	0
JN635301	AY195754	99.975	94.12	1	0	0	0	0	0	1	0	3	0	0
AY495198	AY195754	99.96875	94.12	1	0	0	0	0	0	1	1	0	0	0
AY495208	AY495198	99.9875	94.12	1	0	0	0	0	0	1	1	0	1	0
JQ797795	EF177431	99.9625	94.12	1	0	0	0	0	0	1	10	0	0	0
JQ797796	EF177431	99.96938	94.12	1	0	0	0	0	0	1	11	0	0	0
AY495202	AY495198	99.9625	94.12	1	0	0	0	0	0	1	2	0	0	0
AY495210	AY495198	99.9625	94.12	1	0	0	0	0	0	1	3	0	0	0
AY495216	AY495210	100	94.12	1	0	0	0	0	0	1	3	0	0	0
FJ447985	AY495210	99.99375	94.12	1	0	0	0	0	0	1	3	0	0	1
FJ348177	AY495198	99.96875	94.12	1	0	0	0	0	0	1	4	0	0	0
FJ348216	AY495198	99.9625	94.12	1	0	0	0	0	0	1	5	0	0	0
JQ797787	JN635301	99.9625	94.12	1	0	0	0	0	0	1	6	0	0	0
JQ797792	HQ384194	99.96875	94.12	1	0	0	0	0	0	1	7	0	0	0
JQ797793	JN635301	99.96875	94.12	1	0	0	0	0	0	1	8	0	0	0
JQ797794	EF177431	99.9625	94.12	1	0	0	0	0	0	1	9	0	0	0
HM852774	AY495221	99.95625	94.12	1	0	0	0	0	0	10	0	0	0	0
JN635299	GU906781	99.95688	94.12	1	0	0	0	0	0	11	0	0	0	0
JQ797788	HQ384194	99.95625	94.12	1	0	0	0	0	0	12	0	0	0	0
JQ797797	JN635301	99.95688	94.12	1	0	0	0	0	0	13	0	0	0	0
JQ797810	JQ797809	99.95625	94.12	1	0	0	0	0	0	14	0	0	0	0
JQ797861	EU284668	99.95688	94.12	1	0	0	0	0	0	15	0	0	0	0
JQ797872	AY495212	99.95625	94.12	1	0	0	0	0	0	16	0	0	0	0
JQ797873	JQ797872	99.95688	94.12	1	0	0	0	0	0	17	0	0	0	0
JQ797874	JQ797816	99.95125	94.12	1	0	0	0	0	0	18	0	0	0	0
JQ797876	JQ797818	99.95062	94.12	1	0	0	0	0	0	19	0	0	0	0
AY714034	AY495208	99.95062	94.12	1	0	0	0	0	0	2	0	0	0	0
DQ358974	AY495221	99.95688	94.12	1	0	0	0	0	0	3	0	0	0	0
EF177422	AY495221	99.95688	94.12	1	0	0	0	0	0	4	0	0	0	0
EF452293	AY714034	99.95062	94.12	1	0	0	0	0	0	5	0	0	0	0
EU073970	AY495209	99.95625	94.12	1	0	0	0	0	0	6	0	0	0	0
EU573192	AY495218	99.95688	94.12	1	0	0	0	0	0	7	0	0	0	0
FJ348202	EU573192	99.97562	94.12	1	0	0	0	0	0	7	0	1	0	0
GU592019	FJ348202	99.9875	94.12	1	0	0	0	0	0	7	0	1	1	0
GU592034	GU592019	99.99375	94.12	1	0	0	0	0	0	7	0	1	1	1
HQ336424	GU592019	99.99375	94.12	1	0	0	0	0	0	7	0	1	1	2
GU949564	FJ348202	99.98125	94.12	1	0	0	0	0	0	7	0	1	2	0
FJ449571	EU573192	99.97562	94.12	1	0	0	0	0	0	7	0	2	0	0
JQ797807	FJ449571	99.9875	94.12	1	0	0	0	0	0	7	0	2	1	0
GQ304746	FJ449571	99.9625	94.12	1	0	0	0	0	0	7	1	0	0	0
EU597522	EF660962	99.95625	94.12	1	0	0	0	0	0	8	0	0	0	0

FJ502349	AY714034	99.95125	94.12	1	0	0	0	0	0	9	0	0	0	0
AY495195	AF346983	99.95	94.12	1	0	0	0	0	1	0	0	0	0	0
JQ797820	JQ797818	99.94438	94.12	1	0	0	0	0	10	0	0	0	0	0
JQ797821	JQ797820	99.94375	94.12	1	0	0	0	0	11	0	0	0	0	0
JQ797846	JQ797816	99.945	94.12	1	0	0	0	0	12	0	0	0	0	0
JQ797880	GU123042	99.94375	94.12	1	0	0	0	0	13	0	0	0	0	0
JQ797885	JQ797849	99.94438	94.12	1	0	0	0	0	14	0	0	0	0	0
JQ797896	EU597552	99.94438	94.12	1	0	0	0	0	15	0	0	0	0	0
JQ797897	JQ797896	99.97562	94.12	1	0	0	0	0	15	0	0	1	0	0
JQ797898	JQ797897	100	94.12	1	0	0	0	0	15	0	0	1	0	0
AY495204	AY495203	99.94375	94.12	1	0	0	0	0	2	0	0	0	0	0
HQ287873	AY495204	99.96875	94.12	1	0	0	0	0	2	0	1	0	0	0
HQ907957	HQ287873	99.97562	94.12	1	0	0	0	0	2	0	1	1	0	0
EF177420	AY195754	99.95	94.12	1	0	0	0	0	3	0	0	0	0	0
EU007880	EF459669	99.94438	94.12	1	0	0	0	0	4	0	0	0	0	0
JQ797895	EU007880	99.9875	94.12	1	0	0	0	0	4	0	0	0	1	0
JQ797894	EU007880	99.9625	94.12	1	0	0	0	0	4	0	1	0	0	0
EU597552	EU007880	99.95625	94.12	1	0	0	0	0	4	1	0	0	0	0
GU122987	EU007880	99.95062	94.12	1	0	0	0	0	4	2	0	0	0	0
JQ797899	EU597552	99.95625	94.12	1	0	0	0	0	4	3	0	0	0	0
EU151466	AY495206	99.94438	94.12	1	0	0	0	0	5	0	0	0	0	0
GU123016	AY495206	99.94375	94.12	1	0	0	0	0	6	0	0	0	0	0
HQ325739	EU007880	99.94375	94.12	1	0	0	0	0	7	0	0	0	0	0
JQ797789	AY714034	99.94375	94.12	1	0	0	0	0	8	0	0	0	0	0
JQ797790	JQ797789	99.98125	94.12	1	0	0	0	0	8	0	0	0	1	0
JQ797791	AY714034	99.94438	94.12	1	0	0	0	0	9	0	0	0	0	0
AF381987	AF346983	99.93812	94.12	1	0	0	0	1	0	0	0	0	0	0
EF556169	AF381987	99.98125	94.12	1	0	0	0	1	0	0	0	0	1	0
JN648827	EF556169	100	94.12	1	0	0	0	1	0	0	0	0	1	0
DQ282488	AF381987	99.975	94.12	1	0	0	0	1	0	0	0	1	0	0
DQ282489	DQ282488	100	94.12	1	0	0	0	1	0	0	0	1	0	0
DQ282490	DQ282488	100	94.12	1	0	0	0	1	0	0	0	1	0	0
DQ282492	DQ282488	100	94.12	1	0	0	0	1	0	0	0	1	0	0
GU390312	DQ282488	100	94.12	1	0	0	0	1	0	0	0	1	0	0
JQ797772	DQ282488	99.9875	94.12	1	0	0	0	1	0	0	0	1	1	0
EF660985	EF556169	99.96875	94.12	1	0	0	0	1	0	0	1	0	0	0
HM852838	EF556169	99.96312	94.12	1	0	0	0	1	0	0	2	0	0	0
JQ797773	EF660985	99.96875	94.12	1	0	0	0	1	0	0	3	0	0	0
JQ797774	JQ797773	99.975	94.12	1	0	0	0	1	0	0	3	1	0	0
DQ282491	AF381987	99.95625	94.12	1	0	0	0	1	0	1	0	0	0	0
JQ797782	DQ282491	99.9625	94.12	1	0	0	0	1	0	1	1	0	0	0

EF583175	DQ282491	99.95688	94.12	1	0	0	0	1	0	2	0	0	0	0
EF583177	EF583175	99.9875	94.12	1	0	0	0	1	0	2	0	0	1	0
HM594676	EF583175	99.9875	94.12	1	0	0	0	1	0	2	0	0	2	0
HM852835	EF556169	99.95625	94.12	1	0	0	0	1	0	3	0	0	0	0
JQ797771	EF556169	99.95688	94.12	1	0	0	0	1	0	4	0	0	0	0
JQ797775	EF583175	99.95062	94.12	1	0	0	0	1	0	5	0	0	0	0
JQ797779	HM852835	99.95062	94.12	1	0	0	0	1	0	6	0	0	0	0
JQ797781	DQ282491	99.95062	94.12	1	0	0	0	1	0	7	0	0	0	0
JQ797784	DQ282491	99.95688	94.12	1	0	0	0	1	0	8	0	0	0	0
JQ797768	JQ797767	99.93188	94.12	1	0	0	0	10	0	0	0	0	0	0
JQ797776	EF556169	99.93125	94.12	1	0	0	0	11	0	0	0	0	0	0
JQ797777	JQ797772	99.93125	94.12	1	0	0	0	12	0	0	0	0	0	0
JQ797778	JQ797777	99.975	94.12	1	0	0	0	12	0	0	0	1	0	0
JQ797780	DQ282491	99.93875	94.12	1	0	0	0	13	0	0	0	0	0	0
JQ797783	EF556169	99.9325	94.12	1	0	0	0	14	0	0	0	0	0	0
JQ797786	JQ797773	99.93125	94.12	1	0	0	0	15	0	0	0	0	0	0
JQ797877	AY495206	99.93812	94.12	1	0	0	0	16	0	0	0	0	0	0
JQ797878	JQ797877	99.95625	94.12	1	0	0	0	16	0	1	0	0	0	0
JQ797879	JQ797816	99.93875	94.12	1	0	0	0	17	0	0	0	0	0	0
JQ797893	HQ325739	99.93812	94.12	1	0	0	0	18	0	0	0	0	0	0
JQ797900	HM453206	99.93875	94.12	1	0	0	0	19	0	0	0	0	0	0
AY714033	AF381987	99.93812	94.12	1	0	0	0	2	0	0	0	0	0	0
JQ797901	JQ797895	99.93812	94.12	1	0	0	0	20	0	0	0	0	0	0
EF397558	AY714033	99.93188	94.12	1	0	0	0	3	0	0	0	0	0	0
FJ624455	EF397558	100	94.12	1	0	0	0	3	0	0	0	0	0	0
JF929909	EF397558	99.95688	94.12	1	0	0	0	3	0	1	0	0	0	0
JF939049	JF929909	99.95688	94.12	1	0	0	0	3	0	2	0	0	0	0
JQ797767	JF939049	99.975	94.12	1	0	0	0	3	0	2	0	1	0	0
JQ797769	JF929909	99.95688	94.12	1	0	0	0	3	0	3	0	0	0	0
EF397562	EF397558	99.95	94.12	1	0	0	0	3	1	0	0	0	0	0
EF556155	EF397562	99.9625	94.12	1	0	0	0	3	1	0	1	0	0	0
JQ797766	EF397562	99.9625	94.12	1	0	0	0	3	1	0	2	0	0	0
FJ348161	EU151466	99.93812	94.12	1	0	0	0	4	0	0	0	0	0	0
FJ348162	FJ348161	99.98188	94.12	1	0	0	0	4	0	0	0	0	1	0
FJ348163	FJ348162	100	94.12	1	0	0	0	4	0	0	0	0	1	0
FJ348164	FJ348162	100	94.12	1	0	0	0	4	0	0	0	0	1	0
FJ348165	FJ348162	100	94.12	1	0	0	0	4	0	0	0	0	1	0
GQ304740	FJ348162	100	94.12	1	0	0	0	4	0	0	0	0	1	0
HM171294	FJ348162	99.98188	94.12	1	0	0	0	4	0	0	0	0	2	0
HQ876599	FJ348162	99.975	94.12	1	0	0	0	4	0	0	0	1	0	0
JF812166	FJ348162	99.975	94.12	1	0	0	0	4	0	0	0	2	0	0

JQ797855	FJ348162	99.975	94.12	1	0	0	0	4	0	0	0	3	0	0
JQ797857	FJ348162	99.975	94.12	1	0	0	0	4	0	0	0	4	0	0
JQ797858	JQ797857	99.98125	94.12	1	0	0	0	4	0	0	0	4	1	0
JQ797859	JQ797857	99.975	94.12	1	0	0	0	4	0	0	0	5	0	0
JQ797860	JQ797855	99.975	94.12	1	0	0	0	4	0	0	0	6	0	0
HM627319	FJ348162	99.96938	94.12	1	0	0	0	4	0	0	1	0	0	0
JQ797856	FJ348162	99.9625	94.12	1	0	0	0	4	0	0	2	0	0	0
HM453206	EU007880	99.9375	94.12	1	0	0	0	5	0	0	0	0	0	0
HM852827	EF556169	99.93188	94.12	1	0	0	0	6	0	0	0	0	0	0
HM992836	HM852827	99.95062	94.12	1	0	0	0	6	0	1	0	0	0	0
HQ637485	HM992836	99.99375	94.12	1	0	0	0	6	0	1	0	0	0	1
HM852874	AY495195	99.93812	94.12	1	0	0	0	7	0	0	0	0	0	0
HQ914447	EF459669	99.9325	94.12	1	0	0	0	8	0	0	0	0	0	0
JQ797765	EF397558	99.93125	94.12	1	0	0	0	9	0	0	0	0	0	0
AY495231	AF381987	99.92562	94.12	1	0	0	1	0	0	0	0	0	0	0
AY495232	AY495231	99.96875	94.12	1	0	0	1	0	0	0	1	0	0	0
AY495233	AY495232	99.9875	94.12	1	0	0	1	0	0	0	1	0	1	0
AY495237	AY495233	99.99375	94.12	1	0	0	1	0	0	0	1	0	1	1
AY714035	AY495233	99.99375	94.12	1	0	0	1	0	0	0	1	0	1	2
AY495234	AY495233	99.9875	94.12	1	0	0	1	0	0	0	1	0	2	0
AY495235	AY495233	99.9875	94.12	1	0	0	1	0	0	0	1	0	3	0
HM856621	AY714035	99.98125	94.12	1	0	0	1	0	0	0	1	0	4	0
JQ797761	AY714035	99.98125	94.12	1	0	0	1	0	0	0	1	0	5	0
AY495236	AY495233	99.975	94.12	1	0	0	1	0	0	0	1	1	0	0
EF660916	AY714035	99.97562	94.12	1	0	0	1	0	0	0	1	2	0	0
FJ213450	AY714035	99.975	94.12	1	0	0	1	0	0	0	1	3	0	0
HQ286325	FJ213450	99.99375	94.12	1	0	0	1	0	0	0	1	3	0	1
HM600785	FJ213450	99.975	94.12	1	0	0	1	0	0	0	1	4	0	0
JN635298	AY714035	99.975	94.12	1	0	0	1	0	0	0	1	5	0	0
AY495238	AY495233	99.96875	94.12	1	0	0	1	0	0	0	2	0	0	0
FJ178380	AY714035	99.96875	94.12	1	0	0	1	0	0	0	3	0	0	0
HQ543056	AY495231	99.96875	94.12	1	0	0	1	0	0	0	4	0	0	0
JF286633	AY714035	99.96938	94.12	1	0	0	1	0	0	0	5	0	0	0
JQ797760	AY495238	99.96875	94.12	1	0	0	1	0	0	0	6	0	0	0
EU915478	AY714035	99.95688	94.12	1	0	0	1	0	0	1	0	0	0	0
JQ797763	AY714035	99.94375	94.12	1	0	0	1	0	1	0	0	0	0	0
JQ797764	JQ797763	99.95625	94.12	1	0	0	1	0	1	1	0	0	0	0
HM852784	EF397558	99.925	94.12	1	0	0	2	0	0	0	0	0	0	0
HM852793	EU007859	99.92562	94.12	1	0	0	3	0	0	0	0	0	0	0
HM852829	EU007880	99.92625	94.12	1	0	0	4	0	0	0	0	0	0	0
HQ166708	AY495210	99.92625	94.12	1	0	0	5	0	0	0	0	0	0	0

HQ839858	AY495211	99.92625	94.12	1	0	0	6	0	0	0	0	0	0	0
JQ797770	AY714033	99.92562	94.12	1	0	0	7	0	0	0	0	0	0	0
AF382001	AF346983	99.91375	94.12	1	0	1	0	0	0	0	0	0	0	0
JQ797892	AF382001	99.94438	94.12	1	0	1	0	0	1	0	0	0	0	0
EF184636	AF382001	99.93812	94.12	1	0	1	0	1	0	0	0	0	0	0
JQ797888	EF184636	99.9875	94.12	1	0	1	0	1	0	0	0	0	1	0
JQ797889	JQ797888	99.99375	94.12	1	0	1	0	1	0	0	0	0	1	1
JQ797890	JQ797888	99.98125	94.12	1	0	1	0	1	0	0	0	0	2	0
JQ797891	JQ797890	99.9875	94.12	1	0	1	0	1	0	0	0	0	3	0
JQ797887	EF184636	99.95062	94.12	1	0	1	0	1	0	1	0	0	0	0
JF292900	EF184636	99.94438	94.12	1	0	1	0	1	1	0	0	0	0	0
JQ797886	EF184636	99.94438	94.12	1	0	1	0	1	2	0	0	0	0	0
HM852780	AF382001	99.93188	94.12	1	0	1	0	2	0	0	0	0	0	0
DQ341088	AF382001	99.91938	94.12	1	0	2	0	0	0	0	0	0	0	0
HM852779	AY714035	99.91375	94.12	1	0	3	0	0	0	0	0	0	0	0
JQ797762	HM852779	99.91312	94.12	1	0	4	0	0	0	0	0	0	0	0
JQ797785	JQ797772	99.91938	94.12	1	0	5	0	0	0	0	0	0	0	0
JQ797883	JQ797818	99.92	94.12	1	0	6	0	0	0	0	0	0	0	0
JQ797902	EU007880	99.9075	94.12	1	1	0	0	0	0	0	0	0	0	0
AY195778	AF382001	99.8775	94.12	2	0	0	0	0	0	0	0	0	0	0
JQ797941	AY195778	99.9625	94.12	2	0	0	0	0	0	0	1	0	0	0
DQ523653	AY195778	99.94375	94.12	2	0	0	0	0	1	0	0	0	0	0
DQ523671	DQ523653	99.9625	94.12	2	0	0	0	0	1	0	1	0	0	0
JQ797940	EU597520	99.96875	94.12	2	0	0	0	0	1	0	10	0	0	0
JQ797945	JQ797937	99.96375	94.12	2	0	0	0	0	1	0	11	0	0	0
JQ797946	JQ797945	99.9875	94.12	2	0	0	0	0	1	0	11	0	1	0
JQ797947	EU597520	99.96875	94.12	2	0	0	0	0	1	0	12	0	0	0
JQ797948	EU597520	99.96312	94.12	2	0	0	0	0	1	0	13	0	0	0
JQ797949	EU597520	99.9625	94.12	2	0	0	0	0	1	0	14	0	0	0
JQ797953	JQ797946	99.96312	94.12	2	0	0	0	0	1	0	15	0	0	0
JQ797961	EU597520	99.9625	94.12	2	0	0	0	0	1	0	16	0	0	0
JQ797963	JQ797961	99.9625	94.12	2	0	0	0	0	1	0	17	0	0	0
JQ797966	JQ797939	99.9625	94.12	2	0	0	0	0	1	0	18	0	0	0
JQ797967	JQ797966	100	94.12	2	0	0	0	0	1	0	18	0	0	0
JQ797968	JQ797939	99.96938	94.12	2	0	0	0	0	1	0	19	0	0	0
EU597520	DQ523671	99.96938	94.12	2	0	0	0	0	1	0	2	0	0	0
EU673448	EU597520	99.975	94.12	2	0	0	0	0	1	0	2	1	0	0
EU862198	EU673448	99.9625	94.12	2	0	0	0	0	1	0	3	0	0	0
FJ380056	EU673448	99.9625	94.12	2	0	0	0	0	1	0	4	0	0	0
FJ445408	FJ380056	99.99375	94.12	2	0	0	0	0	1	0	4	0	0	1
GQ249257	EU673448	99.96875	94.12	2	0	0	0	0	1	0	5	0	0	0

JF915700	EU673448	99.9625	94.12	2	0	0	0	0	1	0	6	0	0	0
JF938916	EU673448	99.96875	94.12	2	0	0	0	0	1	0	7	0	0	0
JN635305	JF938916	99.975	94.12	2	0	0	0	0	1	0	7	1	0	0
JQ797937	JN635305	99.98125	94.12	2	0	0	0	0	1	0	7	1	1	0
JN635304	EU597520	99.96875	94.12	2	0	0	0	0	1	0	8	0	0	0
JN635306	JN635304	99.9875	94.12	2	0	0	0	0	1	0	8	0	1	0
JQ797939	JN635304	99.98125	94.12	2	0	0	0	0	1	0	8	0	2	0
JQ797938	JN635305	99.96938	94.12	2	0	0	0	0	1	0	9	0	0	0
FJ445409	EU673448	99.95625	94.12	2	0	0	0	0	1	1	0	0	0	0
JQ797936	JF938916	99.95062	94.12	2	0	0	0	0	1	2	0	0	0	0
JQ797943	EU597520	99.95625	94.12	2	0	0	0	0	1	3	0	0	0	0
JQ797944	JQ797943	99.9875	94.12	2	0	0	0	0	1	3	0	0	1	0
JQ797950	EU673448	99.95625	94.12	2	0	0	0	0	1	4	0	0	0	0
JQ797951	JQ797950	99.99375	94.12	2	0	0	0	0	1	4	0	0	0	1
JQ797952	JQ797951	99.95688	94.12	2	0	0	0	0	1	5	0	0	0	0
JQ797960	JQ797939	99.95062	94.12	2	0	0	0	0	1	6	0	0	0	0
FJ213765	EU673448	99.95	94.12	2	0	0	0	0	2	0	0	0	0	0
HQ889848	FJ213765	100	94.12	2	0	0	0	0	2	0	0	0	0	0
JQ797972	FJ213765	99.98125	94.12	2	0	0	0	0	2	0	0	0	1	0
JQ797973	JQ797972	99.96875	94.12	2	0	0	0	0	2	0	1	0	0	0
JQ797942	JQ797939	99.95	94.12	2	0	0	0	0	3	0	0	0	0	0
JQ797954	EU597520	99.94375	94.12	2	0	0	0	0	4	0	0	0	0	0
JQ797955	JQ797954	100	94.12	2	0	0	0	0	4	0	0	0	0	0
JQ797956	JQ797954	99.99375	94.12	2	0	0	0	0	4	0	0	0	0	1
JQ797957	JQ797939	99.94438	94.12	2	0	0	0	0	5	0	0	0	0	0
JQ797958	JQ797957	99.9875	94.12	2	0	0	0	0	5	0	0	0	1	0
JQ797959	JQ797958	100	94.12	2	0	0	0	0	5	0	0	0	1	0
JQ797962	JQ797939	99.94438	94.12	2	0	0	0	0	6	0	0	0	0	0
JQ797974	JQ797972	99.95	94.12	2	0	0	0	0	7	0	0	0	0	0
EF556152	AY195778	99.93188	94.12	2	0	0	0	1	0	0	0	0	0	0
HQ727682	EF556152	99.96875	94.12	2	0	0	0	1	0	0	1	0	0	0
JQ797969	HQ727682	99.98125	94.12	2	0	0	0	1	0	0	1	0	1	0
JQ797970	JQ797969	100	94.12	2	0	0	0	1	0	0	1	0	1	0
JQ797964	EU597520	99.9325	94.12	2	0	0	0	2	0	0	0	0	0	0
JQ797965	JQ797939	99.93875	94.12	2	0	0	0	3	0	0	0	0	0	0
JQ797971	JQ797939	99.93812	94.12	2	0	0	0	4	0	0	0	0	0	0
DQ341090	AY195778	99.92562	94.12	2	0	0	1	0	0	0	0	0	0	0
EU807741	EU673448	99.91375	94.12	2	0	1	0	0	0	0	0	0	0	0
DQ341089	AY495212	99.8275	94.12	3	0	0	0	0	0	0	0	0	0	0
DQ523640	DQ341089	99.895	94.12	4	0	0	0	0	0	0	0	0	0	0
EU721734	DQ523640	99.94438	94.12	4	0	0	0	0	1	0	0	0	0	0

JF343122	EU721734	99.99375	94.12	4	0	0	0	0	1	0	0	0	0	1
FJ194438	EU721734	99.98125	94.12	4	0	0	0	0	1	0	0	0	1	0
FJ348157	FJ194438	99.99375	94.12	4	0	0	0	0	1	0	0	0	1	1
HQ104930	FJ348157	100	94.12	4	0	0	0	0	1	0	0	0	1	1
GU903270	FJ348157	99.99375	94.12	4	0	0	0	0	1	0	0	0	1	2
HM485691	GU903270	100	94.12	4	0	0	0	0	1	0	0	0	1	2
HQ436101	FJ348157	99.99375	94.12	4	0	0	0	0	1	0	0	0	1	3
GU123013	FJ348157	99.9875	94.12	4	0	0	0	0	1	0	0	0	2	0
HQ384196	FJ348157	99.9875	94.12	4	0	0	0	0	1	0	0	0	3	0
HQ424014	FJ348157	99.98125	94.12	4	0	0	0	0	1	0	0	0	4	0
JN635303	FJ348157	99.98125	94.12	4	0	0	0	0	1	0	0	0	5	0
JQ797907	FJ348157	99.98125	94.12	4	0	0	0	0	1	0	0	0	6	0
JQ797903	GU903270	99.96875	94.12	4	0	0	0	0	1	0	1	0	0	0
JQ797911	JQ797903	99.98125	94.12	4	0	0	0	0	1	0	1	0	1	0
JQ797912	JQ797911	99.975	94.12	4	0	0	0	0	1	0	1	1	0	0
JQ797913	JQ797911	99.975	94.12	4	0	0	0	0	1	0	1	2	0	0
JQ797905	JQ797903	99.96875	94.12	4	0	0	0	0	1	0	2	0	0	0
JQ797908	JQ797903	99.96875	94.12	4	0	0	0	0	1	0	3	0	0	0
JQ797910	GU903270	99.9625	94.12	4	0	0	0	0	1	0	4	0	0	0
FJ605154	FJ348157	99.95688	94.12	4	0	0	0	0	1	1	0	0	0	0
HQ699438	GU903270	99.9575	94.12	4	0	0	0	0	1	2	0	0	0	0
JQ797906	JQ797905	99.95688	94.12	4	0	0	0	0	1	3	0	0	0	0
JQ797909	JQ797908	99.94438	94.12	4	0	0	0	0	2	0	0	0	0	0
EU931680	EU721734	99.93875	94.12	4	0	0	0	1	0	0	0	0	0	0
JQ797904	JQ797903	99.93812	94.12	4	0	0	0	2	0	0	0	0	0	0
JQ797914	JQ797911	99.9375	94.12	4	0	0	0	3	0	0	0	0	0	0
EF556160	EF177410	99.895	94.12	5	0	0	0	0	0	0	0	0	0	0
EF556164	EF556160	99.975	94.12	5	0	0	0	0	0	0	0	1	0	0
EF660967	EF459669	99.84062	94.12	6	0	0	0	0	0	0	0	0	0	0
FJ460543	EF660967	99.90062	94.12	6	1	0	0	0	0	0	0	0	0	0
JQ797925	FJ460543	99.98125	94.12	6	1	0	0	0	0	0	0	0	1	0
JQ797927	FJ460543	99.95062	94.12	6	1	0	0	0	0	1	0	0	0	0
JQ797924	FJ460543	99.94438	94.12	6	1	0	0	0	1	0	0	0	0	0
JQ797930	JQ797924	99.95625	94.12	6	1	0	0	0	1	1	0	0	0	0
JQ797931	JQ797930	99.9625	94.12	6	1	0	0	0	1	1	1	0	0	0
JQ797932	JQ797931	99.99375	94.12	6	1	0	0	0	1	1	1	0	0	1
JQ797933	JQ797931	99.98125	94.12	6	1	0	0	0	1	1	1	0	1	0
JQ797926	JQ797925	99.94438	94.12	6	1	0	0	0	2	0	0	0	0	0
JQ797928	FJ460543	99.93812	94.12	6	1	0	0	1	0	0	0	0	0	0
JQ797929	JQ797928	99.99375	94.12	6	1	0	0	1	0	0	0	0	0	1
GU065327	FJ460543	99.92625	94.12	6	1	0	1	0	0	0	0	0	0	0

JQ797923	GU065327	99.9625	94.12	6	1	0	1	0	0	0	1	0	0	0
JQ797915	FJ460543	99.92062	94.12	6	1	0	2	0	0	0	0	0	0	0
JQ797916	JQ797915	99.93812	94.12	6	1	0	2	1	0	0	0	0	0	0
JQ797917	JQ797915	99.9325	94.12	6	1	0	2	2	0	0	0	0	0	0
JQ797918	JQ797917	99.9875	94.12	6	1	0	2	2	0	0	0	0	1	0
JQ797920	JQ797917	99.96875	94.12	6	1	0	2	2	0	0	1	0	0	0
JQ797919	JQ797917	99.95688	94.12	6	1	0	2	2	0	1	0	0	0	0
JQ797921	JQ797920	99.95062	94.12	6	1	0	2	2	0	2	0	0	0	0
JQ797922	JQ797920	99.94438	94.12	6	1	0	2	2	1	0	0	0	0	0
FJ460559	FJ460543	99.91313	94.12	6	1	1	0	0	0	0	0	0	0	0
JQ797934	FJ460559	99.9625	94.12	6	1	1	0	0	0	0	1	0	0	0
JQ797935	FJ460559	99.95688	94.12	6	1	1	0	0	0	1	0	0	0	0

**Table S5**. Foot and Mouth Disease Virus					Code											
**query**	**country**	**subject (most similar genome to query genome considering only genomes in rows above the query genome)**	**ANIb %**	**% of aligned fragments**	**C 80%**	**E 90%**	**F 95%**	**G 98%**	**H 99%**	**I 99.5%**	**J 99.6%**	**K 99.7%**	**L 99.8%**	**M 99.9%**	**R 99.95%**	**X 99.9999%**
DQ404158	UK	na	na	na	0	0	0	0	0	0	0	0	0	0	0	0
DQ404159	UK	DQ404158	99.76625	88.88889	0	0	0	0	0	0	0	0	1	0	0	0
DQ404160	UK	DQ404159	99.85125	88.88889	0	0	0	0	0	0	0	0	1	1	0	0
DQ404161	UK	DQ404159	99.645	88.88889	0	0	0	0	0	0	0	1	0	0	0	0
DQ404162	UK	DQ404161	99.3025	88.88889	0	0	0	0	0	1	0	0	0	0	0	0
DQ404163	UK	DQ404162	99.23125	88.88889	0	0	0	0	0	2	0	0	0	0	0	0
DQ404164	UK	DQ404162	99.40125	88.88889	0	0	0	0	0	3	0	0	0	0	0	0
DQ404165	UK	DQ404164	99.535	88.88889	0	0	0	0	0	3	1	0	0	0	0	0
DQ404166	UK	DQ404165	99.975	88.88889	0	0	0	0	0	3	1	0	0	0	0	1
DQ404167	UK	DQ404166	99.90125	88.88889	0	0	0	0	0	3	1	0	0	0	1	0
DQ404168	UK	DQ404166	99.70625	88.88889	0	0	0	0	0	3	1	0	2	0	0	0
DQ404169	UK	DQ404166	99.75625	88.88889	0	0	0	0	0	3	1	0	3	0	0	0
DQ404170	UK	DQ404166	99.85375	88.88889	0	0	0	0	0	3	1	0	0	1	0	0
DQ404171	UK	DQ404170	99.815	88.88889	0	0	0	0	0	3	1	0	0	2	0	0
DQ404172	UK	DQ404171	99.8775	88.88889	0	0	0	0	0	3	1	0	0	3	0	0
DQ404173	UK	DQ404172	99.9625	88.88889	0	0	0	0	0	3	1	0	0	3	0	1
DQ404174	UK	DQ404173	99.95	88.88889	0	0	0	0	0	3	1	0	0	3	1	0
DQ404175	UK	DQ404173	99.9625	88.88889	0	0	0	0	0	3	1	0	0	3	0	2
DQ404176	UK	DQ404175	99.9375	88.88889	0	0	0	0	0	3	1	0	0	3	2	0
DQ404177	UK	DQ404176	99.95125	88.88889	0	0	0	0	0	3	1	0	0	3	2	1
DQ404178	UK	DQ404177	99.96375	88.88889	0	0	0	0	0	3	1	0	0	3	2	2
DQ404179	UK	DQ404178	99.9875	88.88889	0	0	0	0	0	3	1	0	0	3	2	3
DQ404180	UK	DQ404176	99.9375	88.88889	0	0	0	0	0	3	1	0	0	3	3	0
HQ832576	India	DQ404169	86.47875	88.88889	0	1	0	0	0	0	0	0	0	0	0	0
HQ832577	India	HQ832576	91.6975	88.88889	0	1	1	0	0	0	0	0	0	0	0	0
HQ832578	India	HQ832576	91.055	88.88889	0	1	2	0	0	0	0	0	0	0	0	0
HQ832579	India	HQ832578	98.725	88.88889	0	1	2	0	1	0	0	0	0	0	0	0
HQ832580	India	HQ832579	98.615	88.88889	0	1	2	0	2	0	0	0	0	0	0	0
HQ832581	India	HQ832579	98.335	75	0	1	2	0	3	0	0	0	0	0	0	0
HQ832582	India	HQ832579	97.805	88.88889	0	1	2	1	0	0	0	0	0	0	0	0
HQ832583	India	HQ832579	98.73625	88.88889	0	1	2	0	4	0	0	0	0	0	0	0
HQ832584	India	HQ832579	91.2	75	0	1	3	0	0	0	0	0	0	0	0	0
HQ832585	India	HQ832584	91.98714	77.77778	0	1	4	0	0	0	0	0	0	0	0	0
HQ832586	India	HQ832585	92.36875	88.88889	0	1	5	0	0	0	0	0	0	0	0	0
HQ832587	India	HQ832581	91.77857	77.77778	0	1	6	0	0	0	0	0	0	0	0	0

HQ832588	India	HQ832584	92.44429	77.77778	0	1	7	0	0	0	0	0	0	0	0	0
HQ832589	India	HQ832588	94.68429	87.5	0	1	8	0	0	0	0	0	0	0	0	0
HQ832590	India	HQ832581	93.68571	77.77778	0	1	9	0	0	0	0	0	0	0	0	0
HQ832591	India	HQ832590	97.7575	88.88889	0	1	9	1	0	0	0	0	0	0	0	0
HQ832592	India	HQ832590	95.48	87.5	0	1	9	2	0	0	0	0	0	0	0	0
